# Uptake of Ethyl Xanthate to Metal Organic Frameworks

**DOI:** 10.1021/acsomega.3c04539

**Published:** 2023-09-14

**Authors:** Riikka Kuosmanen, Elina Sievänen, Manu Lahtinen

**Affiliations:** Department of Chemistry, University of Jyvaskyla, P.O. Box 35, Jyvaskyla 40014, Finland

## Abstract

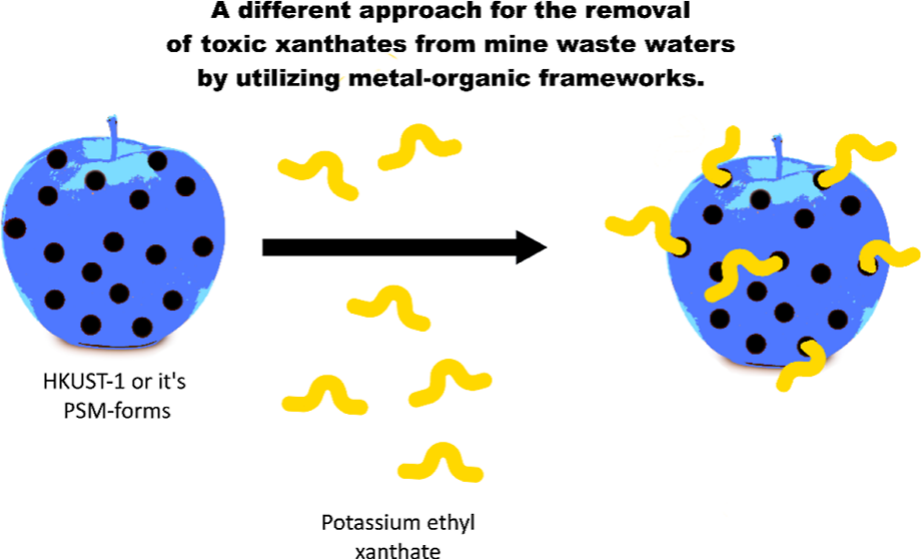

As the mining industry spreads to new areas in the arctic regions, the need for re-useable efficient methods for mine chemicals’ recycling increases. Especially in the case of xanthates, which are used as collectors for many metals from ore. Xanthates are very toxic to aquatic life either directly or indirectly and cause potentially severe health problems to humans after long-term exposure. In the present work, potassium ethyl xanthate (KEX) was observed to coordinate into metal organic frameworks (MOFs). HKUST-1 and its post-synthetically modified forms were observed to behave most effectively of the studied MOFs at low concentrations of KEX. Differences in the uptake of KEX were detected regarding the synthesis method in the case of MIL-100(Fe) synthetized by solvothermal and mechanochemical methods. Other studied MOFs, UiO-66 and MIL-100(Al)/MIL-96(Al), were not observed to be effective in KEX uptake.

## Introduction

Metal organic frameworks (MOFs) have increasingly intrigued scientists in different fields of science for the past few decades.^1−3^ A myriad of applications in different fields of science are being developed: hybrid^4^ and composite^3,5,6^ materials, catalysis,^6−11^ gas adsorption and storage,^12−14^ water harvesting from air,^15^ various (opto)electronic devices,^16,17^ medical use (such as drug transport),^18,19^ and water purification,^6,8,20−26^ to name a few. In water purification and environmental remediation, MOFs have been studied especially in the absorption of divergent organic molecules^21−24,26^ and heavy metals.^20,22−26^ To the best of our knowledge, thus far MOFs have not been utilized in the purification of mine waste waters.

**Figure 1:**
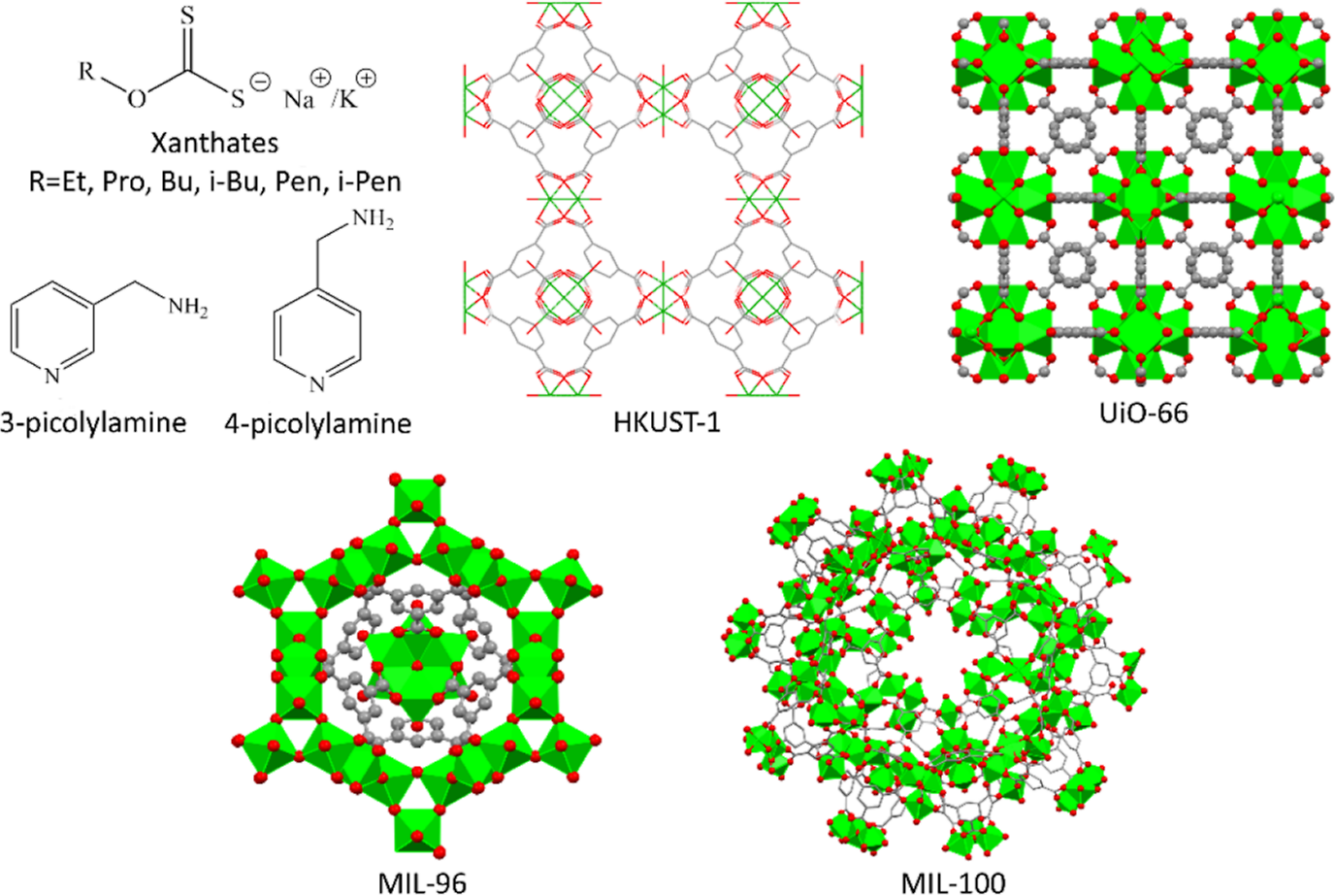
Typical xanthates, amines used in the post-synthetical modification of HKUST-1, and partial crystal structure views of the MOFs studied.

Xanthates (Figure 1) are used in flotation processes in the collection of Zn, Cu, Au, Fe, Ni etc.^27−30^ Although xanthates are widely used due to the cost-effectiveness, xanthates are one of the major problems in the mining industry around the world, even more so in arctic areas.^31^ The high toxicity of xanthates to algae and different bacteria in water ecosystems means that even a very small amount of xanthates is lethal (less than 1 mg/L). In fish, xanthates accumulate heavy metals, which in turn are transferred through food chains even further to predators and humans. When humans are exposed to xanthates in the long-term neurological problems and chronic liver damage can occur.^32^ Xanthates also produce highly toxic compounds, such as CS_2_ among others, as they decompose in wastewater ponds and natural waters.^28,32−36^ As the number of mines is due to increase in Finland, as well as in other arctic areas, there is a need for methods effectively collecting xanthates from wastewater to prevent environmental and health issues.

Worldwide the consumption of xanthates is predicted to grow to nearly 372 million tons per year by 2025.^37^ This combined with the fact that only half of the xanthates are consumed during the flotation process^38^ speaks for the need for efficient and re-useable collection methods for xanthates. To date, many methods for decreasing the xanthate levels in wastewater have been developed.^36^ These methods can be divided roughly into two categories: destructive methods and collection materials. In destructive methods, xanthates are cleaved to smaller molecules by acid decomposition or either conventional or advanced chemical oxidation. Advanced chemical oxidation includes ozone oxidation, as well as Fenton and photocatalytical methods.^39−43^ Materials that collect the xanthate from wastewaters are usually based on zeolite-type materials.^37,44−46^ These materials produce a great amount of waste, as they can be used only once. For sulfate anions, there are already some re-useable materials under development, based on resins^47^ and modified silica gels.^47,48^ Even biological methods^38,49−51^ are available for xanthates, utilizing divergent bacteria and algae, but these methods cannot be applied during cold winters of the arctic regions.

Based on the above-mentioned observations, the objective of this study was to take the first steps toward the re-useable method for uptake of xanthates. In the current study, uptake of potassium ethyl xanthate (KEX, Figure 1) was studied by utilizing known, relatively effortlessly prepared, and large pore size possessing MOFs. A large pore size was considered to be relevant, as some of the xanthates used in the mining industry are quite large in size. The MOFs selected were HKUST-1, MIL-100(Fe), MIL-100(Al), and UiO-66 (Figure 1). In the case of HKUST-1, two post-synthetically modified forms with 3-picolylamine (3-PA) or 4-picolylamine (4-PA) (Figure 1) were studied. MIL-100(Fe) was synthetized by two different methods: solvothermal and mechanochemical syntheses. Regarding Al-containing MOF MIL-100, the synthesis produced two different MOFs depending on the cooling rate. MIL-100(Al) was obtained by rapid cooling and approximately 1:1 MIL-100(Al):MIL-96(Al) (Figure 1) with passive cooling to room temperature. However, the divergent product composition did not affect the obtained results; thus, their behavior in measurements was uniform.

## Results and Discussion

### Experimental Section

Detailed descriptions of the syntheses can be found in the Supporting Information.

### Powder X-ray Diffraction

**Figure 2:**
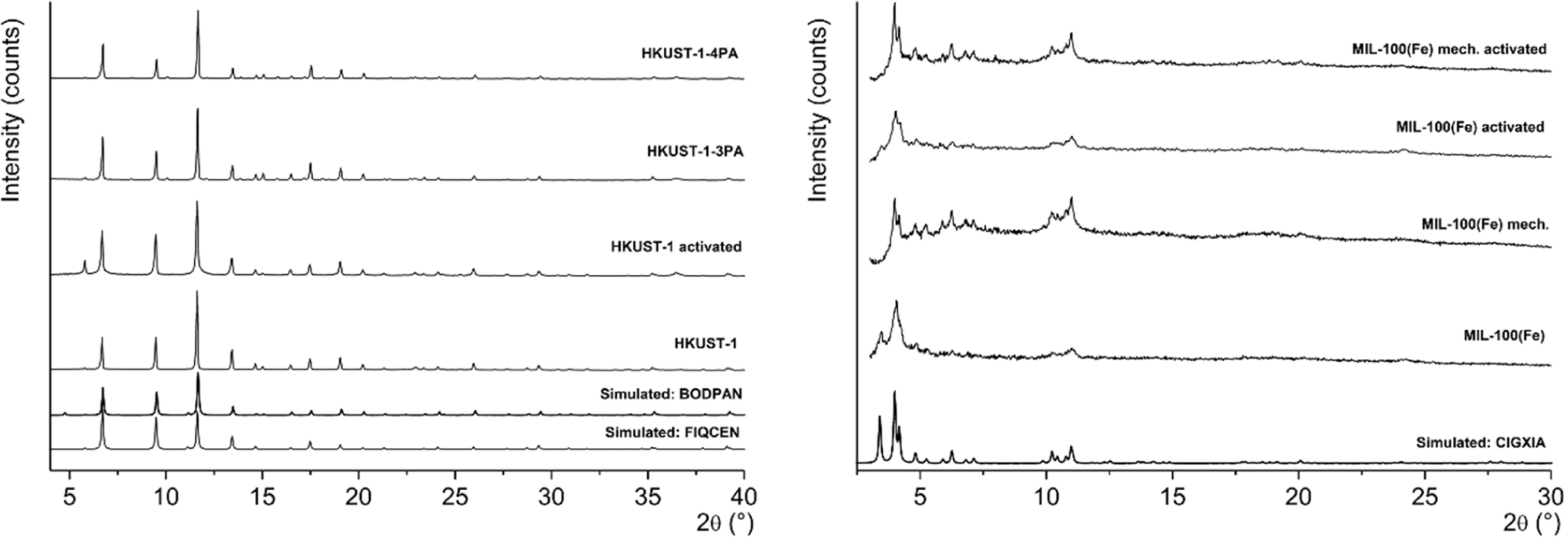
Powder X-ray diffraction (PXRD patterns of HKUST-1 and MIL-100(Fe) MOFs.

**Figure 3:**
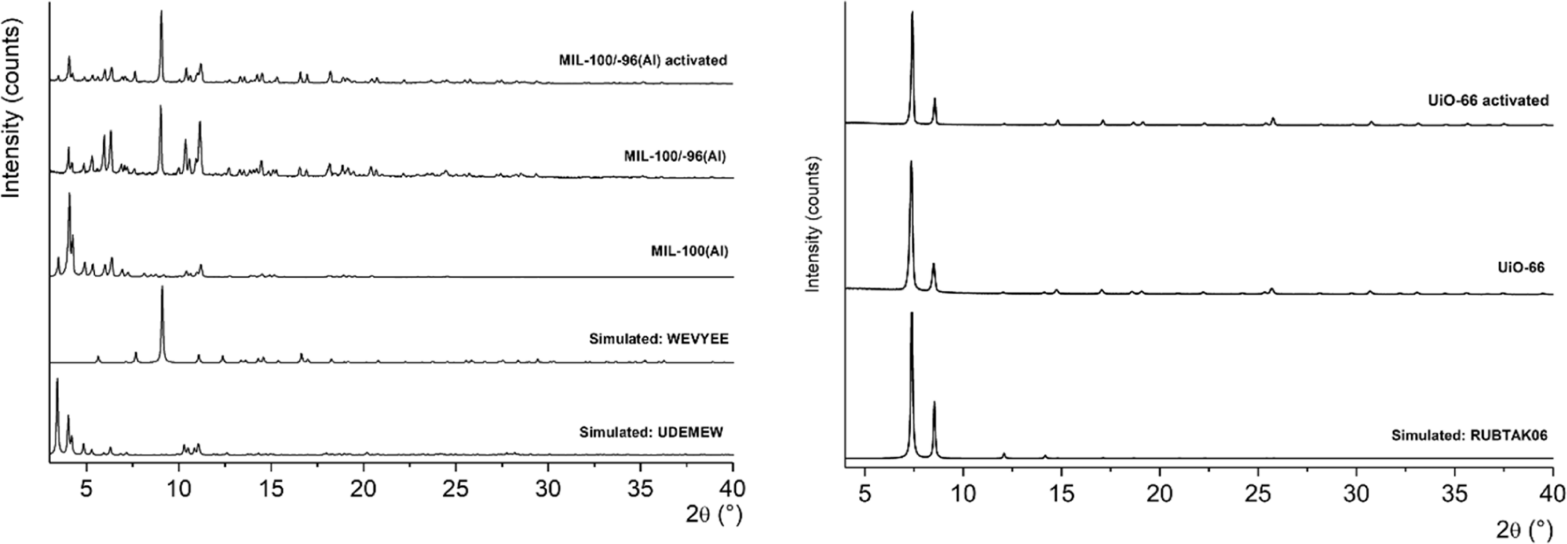
PXRD patterns of MIL-100(Al), MIL-100/-96(Al) and UiO-66 MOFs.

Powder X-ray diffraction (PXRD) measurements were made for pristine (dried in air) and activated (by vacuuming) MOFs to ascertain their structural correspondence to the structural forms reported in the literature. The analyses were made using the Pawley whole pattern fitting method to index the known unit cells, retrieved from Cambridge structural database (CSD),^52^ to the experimental PXRD patterns. The Pawley fit plots and the crystallographic data are shown in the Supporting Information (Figures S1–S13, and Tables S1–S5), whereas the visual comparisons of the PXRD patterns are shown in Figures 2 and 3. As shown in Figure 2 and the Pawley fits (Figures S1, S2 and Table S1), the PXRD patterns of pristine and activated HKUST-1 bulk powders match fully to their simulated correspondence that is generated using the FIQCEN reference structure.^53^ Similarly, the post-synthetically modified HKUST-1’s amine containing 3-PA and 4-PA products match to the pattern generated from the BODPAN structure that is a slightly distorted structure modification reported for HKUST-1 (Figure 2, Figures S3 and S4 and Table S1 in Supporting Information).^54^ This in turn, along with the changed color of bulk powder, indicates that the network structure is amine containing, as its presence causes some degree of crystallographic symmetry disorder in the observed structure. The PXRD patterns of differently prepared MOF-100(Fe) bulk powders (Figures 2, S5–S8 and Table S2 in the Supporting Information), including the solvothermally and mechanochemically prepared variants (both pristine and activated) match well with the reference structure CIGXIA.^62^ Also, based on the measured PXRD patterns, the mechanochemical process generally produces a more crystalline phase than the one produced by the solvothermal reaction, also containing smaller amounts of iron oxide impurities.

For MIL-100 Al-variants, generally two types of PXRD patterns were obtained depending on the preparation conditions of the bulk powders (Figures 3, S9–S11 and Tables S3 and S4 in the Supporting Information). With fast cooling of solvothermal reaction, the crystalline phase corresponding to the isostructural MIL-100(Cr) structure (UDEMEW) formed.^55^ Whereas slower cooling rates ended up to a mixture of MIL-100(Al) and MIL-96(Al) phases, the latter of which is identifiable by the reference structure WEVYEE.^56^ The same crystalline phases were identified also on activated bulk powders. The fourth MOF type, UiO-66, was identified by the reference RUBTAK06, which matched both to pristine and activated bulk powders (Figures 3, S12, S13 and Table S5 in the Supporting Information).^57^

### ^1^H NMR Titrations

^1^H NMR has been utilized in the study of MOFs previously, for example, in the case of HKUST-1.^58,59^ In the studies reported in refs (58) and (59), HKUST-1 was dissolved in deuterated sulfuric acid. In the current study, deuterated water was used for two reasons: to preserve the integrity of the structure of the MOFs and to simulate the end-use of the systems in wastewater purification. In addition, as the resulting system is heterogenous, the original idea was to follow the disappearance of water-soluble potassium ethyl xanthate (KEX) after additions to the suspension of MOF in D_2_O. However, signals arising from the MOFs as well as the small molecules (EtOH or DMF) inside the pristine MOFs were observed in the recorded ^1^H NMR spectra. Because of the heterogenous nature of the titrated MOF samples, solid-state ^13^C NMR, Fourier transform infrared (FTIR) spectroscopy and PXRD were used to verify the results obtained in the titrations. In the case of 1:1 MOF/KEX samples, no changes in the structure of the MOFs were detected based on the PXRD and FTIR data. In Table S6, the ratios of coordinated and free KEX during each titration are presented regarding the highest values of coordinated KEX.

^1^H NMR titrations were performed without and with internal standards and for pristine and activated MOFs. Benzene was used as an internal standard as its signal did not overlap with any signals originating from MOFs or KEX. Intriguingly, very divergent results were obtained in the presence of benzene and with activated MOFs.

In the case of HKUST-1 and its modified forms, striking color changes were seen. The turquoise HKUST-1 changed color to green and eventually yellow during the titration. The activated HKUST-1 changed color from dark violet to bright green immediately after the first addition of KEX. The bright green color was observed after each addition of KEX, until after 1:0.8 (HKUST-1/KEX), a small amount of fluffy olive-green solid substance was observed. However, the amount of this fluffy solid did not increase during the titration until 1:2.8 as the color of this solid started to change to yellow. The post-synthetically modified forms of HKUST-1, with 3-picolylamine (3-PA) and 4-picolylamine (4-PA) experienced color changes as well. HKUST-1 with 3-PA changed from greenish turquoise to yellow. Instead, bluish turquoise HKUST-1 with 4-PA changed to olive green. In addition, in the case mechanochemically synthetized MIL-100(Fe) color change from orange red to dark brown was observed. Other studied MOFs did not experience changes in color.

**Figure 4:**
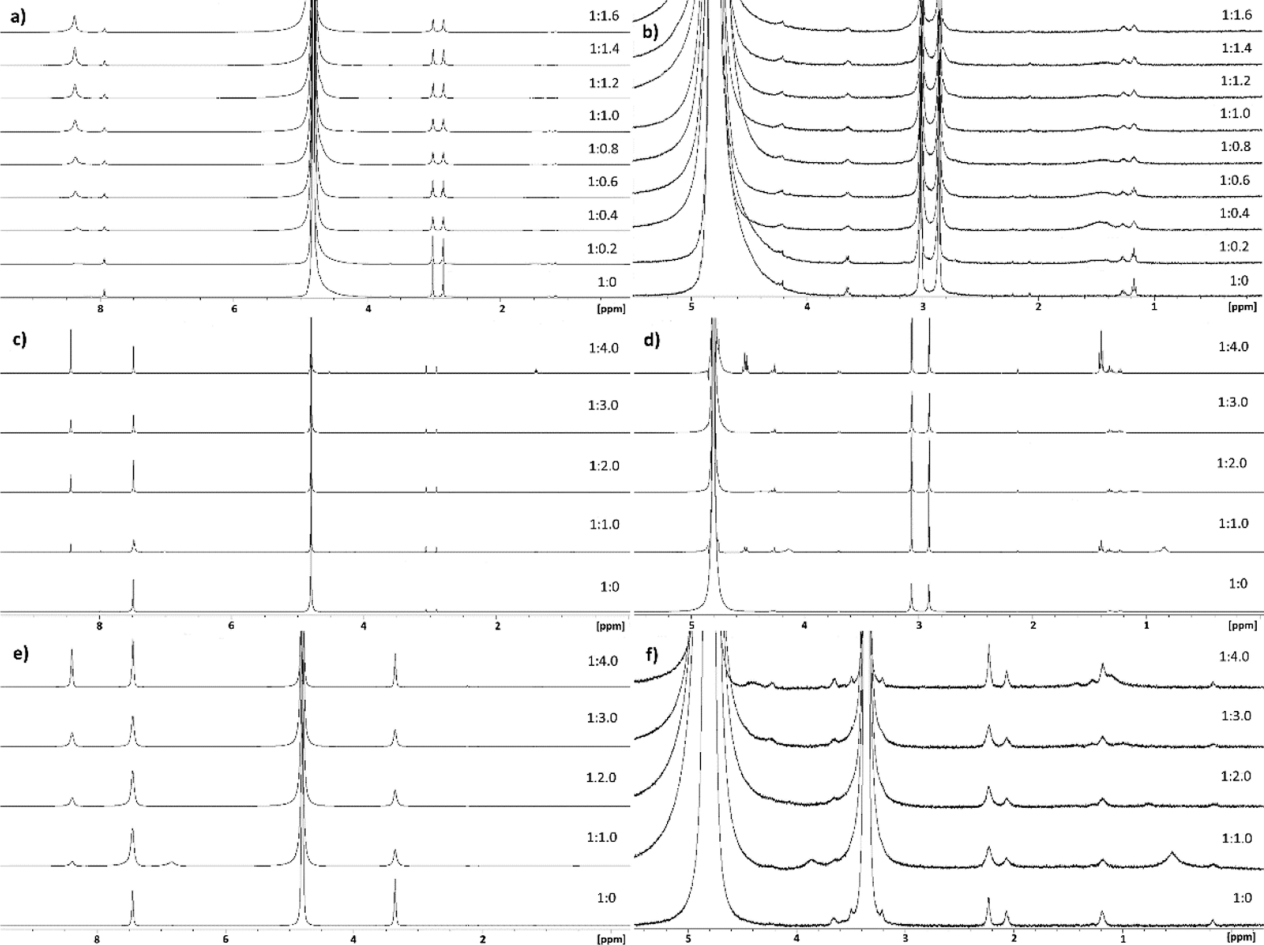
Titrations of HKUST-1. Titration of pristine HKUST-1 without internal standard (a) and aliphatic region (b). Titration of pristine HKUST-1 with internal standard (c) and aliphatic region (d). Titration of activated HKUST-1 with internal standard (e) and aliphatic region (f).

During the titration of pristine HKUST-1 without an internal standard, no signals arising from KEX were observed (Figure 4a,b). In the aromatic region two signals were seen. A signal at 7.93 ppm arising from free trimesic acid (H3btc) inside HKUST-1 was observed. This signal shifts to upfield as the titration progresses. The other aromatic signal at 8.40 ppm is observed after the first addition of KEX and is most likely due to the copper complexes of H3btc from collapsed MOFs. When comparing the spectra of the current study to the previous studies in deuterated sulfuric acid,^58,59^ the shift at 8.4 ppm can be due to the formation of a copper complex of H3btc, which results in less shielded H_b_ protons (protons of the benzene ring). Thus, the shift is observed more downfield than the H_b_ signal of free H3btc inside the MOF. In the aliphatic region (Figure 4b), only signals of impurities of the starting material H3btc are observed in addition to the EtOH inside the MOF (two singlets near 3 ppm). The consistency of the HKUST-1 changed from a powder to flaky but did not change color during the titration.

However, a small amount of free KEX was seen in spectra throughout the titration of pristine HKUST-1 with an internal standard (Figure 4c,d). The quartet arising from CH_2_ protons of the free KEX at 4.52 ppm is seen after 1:0.4 (MOF/KEX). This signal varies in intensity and sometimes disappears (Figure 4d). Similarly, the signal of CH_3_ protons of the free KEX at 1.40 ppm appears at 1:0.4 (MOF/KEX). This signal is not observed from 1:1.6 to 1:3.4 (MOF/KEX). The signals of coordinated KEX are interesting, as they disappear as well. The signal from CH_2_ protons at 3.90 ppm grows until 1:1.2 (MOF/KEX) and is not observed after 1:1.6 (MOF/KEX). In a similar manner, the signal from CH_3_ protons at 0.59 ppm grows until 1:1.2 (MOF/KEX) after which it varies in intensity, finally disappearing at 1:2.8 (MOF/KEX). Both signals arising from coordinated KEX do not possess a fine structure, only blunt singlets are seen as the signals move to downfield. In addition, aliphatic signals arising from impurities of the starting materials as well as EtOH inside the MOF are observed. The ratio of coordinated and free KEX is 7:1 at its best at 1:1.2 (MOF/KEX) when comparing the integrals of the CH_2_ signals of KEX.

The signal due to the aromatic protons of the free ligand (H3btc) was observed to slowly increasing and moving slightly upfield during the titration (Figure 4c) at 7.98 ppm. The other aromatic signal observed at 8.42 ppm moves slightly downfield, increasing steadily until after 1:2.8 (MOF/KEX), when it starts to grow rapidly. This signal can be due to the H_b_ protons of H3btc complexes with copper from the collapsed MOF. The third aromatic signal at 6.87 ppm varies in intensity greatly and is not observed after 1:2.8 (MOF/KEX). This signal may arise from the coordination of KEX to the copper centers of the MOF. The ratio of MOF/KEX 1:2.8 was significant also regarding the color changes observed during the titration: at this point, the color changes to yellow. The observations made from NMR signals as well as the color change occurring simultaneously can indicate the collapse of the MOF toward the end of the titration. The yellow orange color^60^ may indicate the formation of copper xanthate, which rose on top of the solution, and thus no signals are observed.

In the case of activated HKUST-1 with an internal standard (Figure 4e,f), two aromatic signals were observed. The signal indicating the KEX coordinating to the copper nodes of the MOF at 6.91 ppm and increased rapidly until it started to decrease after 1:0.6 (MOF/KEX). This signal is seen throughout the titration, which indicates that the MOF does not collapse completely after the xanthate is coordinated to the copper nodes of MOFs. The other aromatic signal at 8.36 ppm is due to the H3btc complexes with copper from the collapsed MOF. This signal moves downfield and increases somewhat steadily during the titration. After 1:2.4 (MOF/KEX), the signal in question starts to increase slightly more. This observation is quite consistent with the visual observation of color change. Thus, the color of the NMR sample started to turn to yellow at 1:2.8 (MOF/KEX) due to formation of copper xanthate.

In the aliphatic region of the spectrum (Figure 4f), a signal arising from water inside the MOF at 3.35 ppm is observed. Intriguingly, the CH_2_ signal of coordinated KEX was seen during the titration from 1:0.2 to 1:1.2 (MOF/KEX) at 3.84 ppm. This signal is the strongest at 1:0.6, similarly as the aromatic signal at 6.91 ppm due to KEX coordination. Interestingly, at 1:3.8 (MOF/KEX), the CH_2_ signal appears again: two weak signals arising from CH_2_ protons of both free and coordinated KEX are observed. Instead, the CH_3_ signal of coordinated KEX (at 0.53 ppm) is seen through whole titration, moving to downfield and varying in intensity. As no free KEX is observed before the end of the titration, the activated HKUST-1 appears to be even more effective in capturing KEX from water than the pristine form of HKUST-1 in small concentrations of KEX. This observation could be expected because the pores of activated MOFs are free from additional ligand and solvent molecules usually trapped in the pores after synthesis.

The post-synthetically modified (PSM) forms of HKUST-1 showed interesting differences and were measured only as activated. PSM was performed with 3-PA or 4-PA (Figure 1), which led to different behaviors during the titrations. Once again, different results were observed without the internal standard than with it.

In the case of PSM performed with 3-PA, the color changed immediately from greenish turquoise to bright green after the first addition of KEX during the titration without an internal standard. At 1:1.0 (MOF/KEX), the MOF started to turn yellow. Intriguingly, from 1:0.6 to 1:0.8 (MOF/KEX), signals arising from 3-PA were observed (Figures S14 and S15) in the aromatic region. Additionally, signals from CH_3_ and CH_2_ protons of coordinated KEX were seen (0.90 and 3.39 ppm, respectively, see Figure S15). An aromatic signal from H3btc complexes with copper due to the collapsing of the MOF (at 8.43 ppm) increases steadily but decreases significantly at 1:2.0.

The addition of internal standard to HKUST-1 modified with 3-PA affecting the results. The color of the MOF started to turn yellow much later (at 1:2.0 MOF/KEX) and the whole MOF was yellow at 1:4.0 (MOF/KEX). One of the aromatic signals of 3-PA (Figures S16–S18) were observed at 8.51 ppm after the first addition of KEX until the end of the titration at 1:5.0 (MOF/KEX). The rest of the aromatic signals (at 8.55 and 7.91 ppm) and signals from CH_2_ protons (at 3.98 ppm) arising from 3-PA were observed at 1:0.6 (MOF/KEX). When comparing the integrals of the signals originating from 3-PA, the biggest difference between consequent measurements were just before color changes were seen (from 1:1.6 to 1:1.8 MOF/KEX), as is the case of activated HKUST-1 with regard to the H3btc signal from the collapsed MOF. An aromatic signal at 6.89 ppm (from H3btc, affected by the KEX inside the MOF) shifts to downfield as it gradually decreases and finally merges with the signal of benzene protons at 1:2.0 (coincides also with the visually observed color change). At 8.05 ppm, a signal from H_b_ of H3btc coordinated in the MOF network was seen; unfortunately, it overlaps with a signal arising from 3-PA until 1:2.4 (MOF/KEX). After this, the H_b_ signal of H3btc is observed again as the signal from 3-PA moves downfield. The signal of free H_b_ protons of H3btc at 8.43 ppm increases steadily until the titration reaches the point from 1:1.6 to 1:1.8 (MOF/KEX), where the biggest differences in the values of integrals are observed, as in the case of signals from protons of 3-PA.

Furthermore, in the case of HKUST-1 modified with 3-PA and internal standard, signals of coordinated KEX were observed from the beginning of the titration but signals from free KEX were seen from 1:0.6 onward (Figure S18). The ratio of the integrals of CH_2_ protons of coordinated (at 4.51 ppm) and free (at 4.24 ppm) KEX was approximately 3:1 until titration had progressed to the 1:1.0 (MOF/KEX) point. After that the ratio was 1:1 until 1:1.6 (MOF/KEX). From this point forward, the ratio was in favor of free KEX. The signal of CH_2_ protons of coordinated KEX moved downfield gradually and overlaps with the signal of free KEX, thus making the analysis challenging. The corresponding signals of CH_3_ protons showed similar trends regarding the ratio of integral values (at 1.39 and 0.61 ppm for coordinated and free signals, respectively).

HKUST-1 post-synthetically modified with 4-PA behaved in different manners during the titration (Figures S19–S21). Without an internal standard, the color of the MOF changed gradually from blueish turquoise to olive-green, no yellow substance was observed. The H_b_ proton signal from H3btc coordinated into the MOF’s structure was very broad and low. This signal also disappeared after the first addition of KEX. However, the H_b_ signal of free H3btc from the collapsed MOF was observed throughout the titration, increasing gradually. Additionally, the signals arising from the 4-PA were observed after 1:0.6 (MOF/KEX). The signals from KEX, free or coordinated, were virtually impossible to analyze due to their small size and blending into the baseline. From these, results can be concluded that 4-PA affects favorably to the uptake of KEX, as the signals from KEX and color change indicate that the MOF network does not collapse to the same extent than with 3-PA and HKUST-1 itself. Although the H_b_ signal of free H3btc increases steadily during the titration, the MOF network can be deduced not to have collapsed entirely during the process due to the absence of yellow color.

With an internal standard, the HKUST-1 with 4-PA showed different behaviors (Figures S22–S24). Signals due to the 4-PA (both aromatic and aliphatic at 8.57 and 4.31 ppm, respectively) were observed for the first time at 1:3.8 (MOF/KEX), considerably later than in the case of 3-PA and with 4-PA without standards. At 8.40 ppm, a signal arising from H_b_ protons of H3btc is observed before and after the addition of KEX. This signal had varying integral values, and no clear trends were observed. The signals of CH_2_ protons of free and coordinated KEX were seen through the whole titration (at 4.51 and 3.70 ppm, respectively). Both CH_3_ and CH_2_ signals of free and coordinated KEX were most clear at the 1:4.2 (MOF/KEX) stage of the titration. Before this, the signals of coordinated KEX were stronger than the free KEX (regarding the CH_2_ signals), indicating that this modified MOF uptakes effectively KEX at low concentrations. The ratio of the coordinated and free KEX was the highest 2:1 at 1:3.6 (MOF/KEX). In other points of titration, the integration of signals was challenging due to blending into the baseline.

The better stability of HKUST-1 PSM with 4-PA is further supported by the color changes of the system: after the first addition of KEX, the color changed to olive green and after 1:1.2 (MOF/KEX) color started to change to ochre-olive-green (not bright yellow as in the case of HKUST-1). Because the 4-PA’s signals were observed at the end of the titration, it is plausible that the MOF stays quite intact, even though the signal of free H3btc increases and color changes are seen. Both picolylamines utilized in the PSM are highly soluble to water. Thus, signals due to the release of picolylamine should be observed in the case of 4-PA also in the beginning of the titration. It is also possible that the amines used might experience solvent exchange with D_2_O, but that was not observed in this case. Thus, it can be concluded 4-PA enhanced the stability of the HKUST-1 significantly.

Pristine MIL-100(Fe) without an internal standard showed a poor uptake of KEX (Figures S25 and S26). In addition to DMF inside the MOF (two signals near 3 ppm and a signal at 8 ppm), clear signals arising from free KEX were observed (CH_2_ at 4.47 ppm and CH_3_ at 1.34 ppm). The signals from coordinated KEX were very small, CH_2_ at 3.65 ppm and CH_3_ at 1.19 ppm. In the aromatic region, H_b_ proton signals of free H3btc inside the MOF at 8.47 ppm were seen. With an internal standard, the titration of MIL-100(Fe) produced very broad signals (Figures S27–S29). However, characteristic signals from DMF were observed. Only one signal arising from KEX was seen at 1.39 ppm (CH_3_ protons). This signal did not grow in proportion of the additions, as the values of integrals were not growing by the same amount after each addition of KEX. It can be concluded that this MOF uptakes a very small amount of KEX, if at all, because due to the broad signals the integration was difficult. Instead, what was clear from the titration is that MIL-100(Fe) uptakes benzene quite effectively. The signal from coordinated benzene grows throughout the titration (at 6.77 ppm). Similar results were observed with the activated MIL-100(Fe) with an internal standard (Figures S30–S32), although the signals from DMF were considerably smaller. The uptake of benzene was much more favorable than the uptake of KEX in this case also.

When comparing the MIL-100(Fe) prepared by solvothermal and mechanochemical synthesis, spectra look different. Only one aromatic signal is observed in the case of pristine mechanochemically synthesized MIL-100(Fe), as seen in Figures S33 and S34. This signal corresponds to the coordinated H3btc at 8.85 ppm and is observed through the whole titration after the first addition of KEX. Also, the signal moves upfield indicating increased shielding due to the uptake of KEX. In the aliphatic region (Figure S34), signals from CH_3_ and CH_2_ protons of EtOH are seen. At 1:0.4 (MOF/KEX), signals of free KEX (CH_3_ protons at 1.41 ppm) are observed. Because the EtOH signals are so intense, they most probably overlap with any signals from coordinated KEX. The pristine MOF with an internal standard (Figures S35–S37) shows an aromatic signal of the H_b_ protons of H3btc at 8.86 ppm, and the signal shifts to upfield during the titration. Another very small aromatic signal is seen at 9.12 ppm from 1:1.6 to 1:2.6 (MOF/KEX) for which the origin is unknown. In the aliphatic region, signals from both free and coordinated KEX are observed. At the beginning only coordinated KEX is seen (CH_2_ at 3.70 ppm and CH_3_ at 1.23 ppm), but at 1:0.4 (MOF/KEX) one signal of free KEX appears (CH_3_ at 1.40 ppm). At 1:0.6 (MOF/KEX), equal intensity signals are seen from free and coordinated KEX. After this, the signals from free KEX are more intense. This indicates that MOF uptakes only very small amounts of KEX. Interestingly, no uptake of benzene is observed in the case of mechanochemically synthetized MIL-100(Fe) in the pristine form. The activated form with an internal standard (Figures S38–S40) showed the same behavior in the uptake of KEX as the non-activated with an internal standard. In the aromatic region, a signal of unknown origin is seen at 8.61 ppm when titration had progressed to 1:2.8 (MOF/KEX) and was observed to the end of the titration. The uptake of the internal standard, benzene, was observed until the signals merged with the signal from benzene protons at 1:4.0 (MOF/KEX). The color of MOF changed from reddish orange to dark brown during the titration.

Regarding MIL-100(Al), similar results were observed with the pristine MOF with and without internal standards and activated MOFs with internal standards (Figures S41, S42, S43–S45 and S46–S48, respectively). Pristine MIL-100(Al) binds KEX partially, as can be seen in Figures S41 and S42. At 2.28 ppm, a signal arising from impurities of the H3btc reagents is seen in all experiments, even in the activated MOF slightly. Titration with the activated MOF was performed with a product from another synthesis, and the MOF was approximately 1:1 MIL-100(Al)/MIL-96(Al) based on the PXRD analysis. Despite the product being a mixture of MIL-96 and MIL-100, a similar performance of the two different products was observed during the studies. From the first addition of KEX onward, two sets of signals arising from KEX can be observed: coordinated and free forms. During the titration of pristine MOF, the CH_3_ and CH_2_ signals of free KEX were observed at 4.51 and 1.39 ppm, respectively. In the case of coordinated KEX, the CH_3_ and CH_2_ signals were seen at 3.70 and 1.23 ppm, respectively. After the first addition of KEX, the signals from coordinated KEX are more intense (coordinated/free ratio was 1.3:1) in the case of pristine MOF without an internal standard. But, from 1:0.4 (MOF/KEX) onward, the free KEX dominates (coordinated/free ratio approx. 1:2). In other titrations, a similar observation was made from the beginning of the experiment: roughly one-third of the KEX added was taken up by the MOF. With the activated MOF uptake of benzene was seen, no other aromatic signals were observed. No changes in color or consistency of the MOF were observed. Based on NMR spectra, the MOF did not collapse after addition of KEX.

UiO-66’s ability to uptake KEX was the weakest one of the MOFs studied. The pristine MOF without an internal standard showed multiple signals in the aromatic region arising from free and coordinated H2bdc ligands (7.98, 7.93, and 7.72 ppm, respectively) in addition to one unknown signal at 8.50 ppm (Figure S49). In the aliphatic region (Figure S50), multiple signals from the impurities of the ligand reagent were seen. The most clear was the fact that signals from free KEX were predominantly observed (at 4.52 ppm for the CH_2_ signal and 1.39 ppm for the CH_3_ signal) during the titration and no signals of coordinated KEX were seen. With internal standard, the pristine MOF (Figures S51–S53) was observed to uptake benzene (signal at 6.78 ppm), although the benzene signal disappears at 1:2.2 (MOF/KEX). The unknown signal at 8.50 ppm is significantly weaker. Other aromatic signals are similar to the MOF without internal standard. However, no difference in the uptake capacity of KEX was observed. Signals of free KEX were seen at the same NMR shift values as in the case of pristine UiO-66 without an internal standard. Activated MOFs with an internal standard (Figures S54–S56) showed slightly better affinity toward KEX, the ratio between the integrals of free and coordinated KEX was approx. 4:1 throughout the titration. The activated MOF also binds benzene, but the signal of coordinated benzene disappears at 1:1.2 (MOF/KEX). Other aromatic signals were similar to the ones observed with the pristine MOF and were growing steadily. All the signals were slightly more at the upfield when compared to the titrations performed with pristine MOFs.

### ^13^C Solid-State NMR Measurements

For the ^13^C solid-state NMR measurements, pristine HKUST-1 and UiO-66 were selected due to their opposite function in the uptake of KEX. In the case of HKUST-1, the measurements were long (9 days) due to the copper reducing the intensity of signals. A detailed description of the sample preparation and the measurements are provided in the Supporting Information.

**Figure 5:**
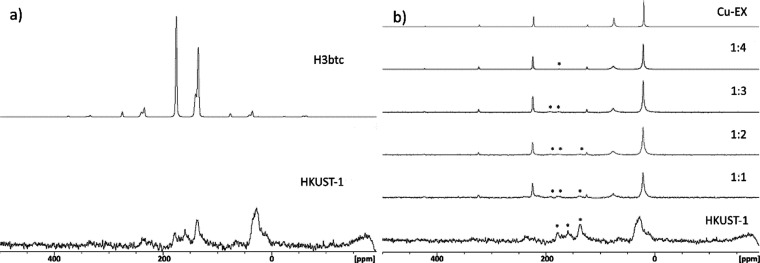
(a) Solid-state spectra of H3btc and HKUST-1. (b) stacked solid-state spectra of HKUST-1, 1:1 to 1:4 (HKUST-1/KEX), and copper ethyl xanthate (Cu-EX). Signals clearly originating from HKUST-1 are marked with an asterisk.

Compared to the H3btc alone, pristine HKUST-1 had signals shifted to the upfield as the coordination to copper influences the signals (Figure 5a). As the amount of KEX is increased from 1:1.0 to 1:4.0 (HKUST-1/KEX), HKUST-1 is observed to collapse. At 1:2.0, one signal of the HKUST-1 is observed, but after that only signals from copper ethyl xanthate are seen. When comparing the 1:4.0 spectrum to the solid-state ^13^C NMR spectrum of the synthetized copper ethyl xanthate, the spectra are nearly identical (Figure 5b).

**Figure 6:**
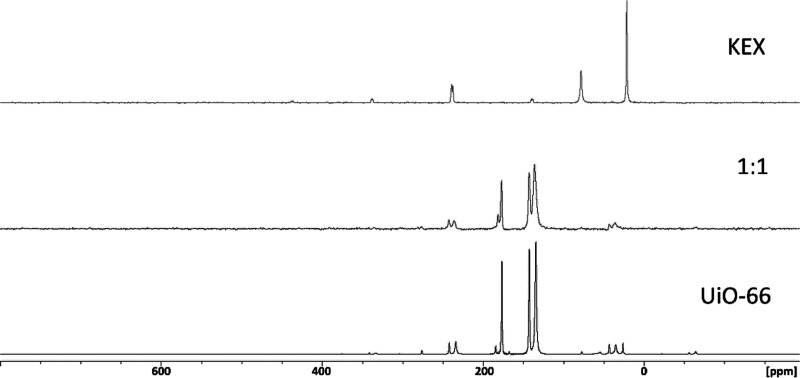
Solid-state spectra of UiO-66 and 1:1 UiO-66/KEX and KEX.

In the case of UiO-66, no signals arising from ethyl xanthate is observed at 1:1.0 (UiO-66/KEX). In the Figure 6, the difference between pristine UiO-66 and UiO-66/KEX 1:1.0 are presented. This verifies the observations made during the ^1^H NMR titrations of UiO-66 being virtually ineffective in the uptake of KEX. The measurements were not continued further as the signals of KEX were not as significantly evident as in the case of HKUST-1.

### FTIR

Samples for FTIR measurements were prepared with a similar procedure as in the case of ^13^C solid-state measurements, see details in the Supporting Information.

**Figure 7:**
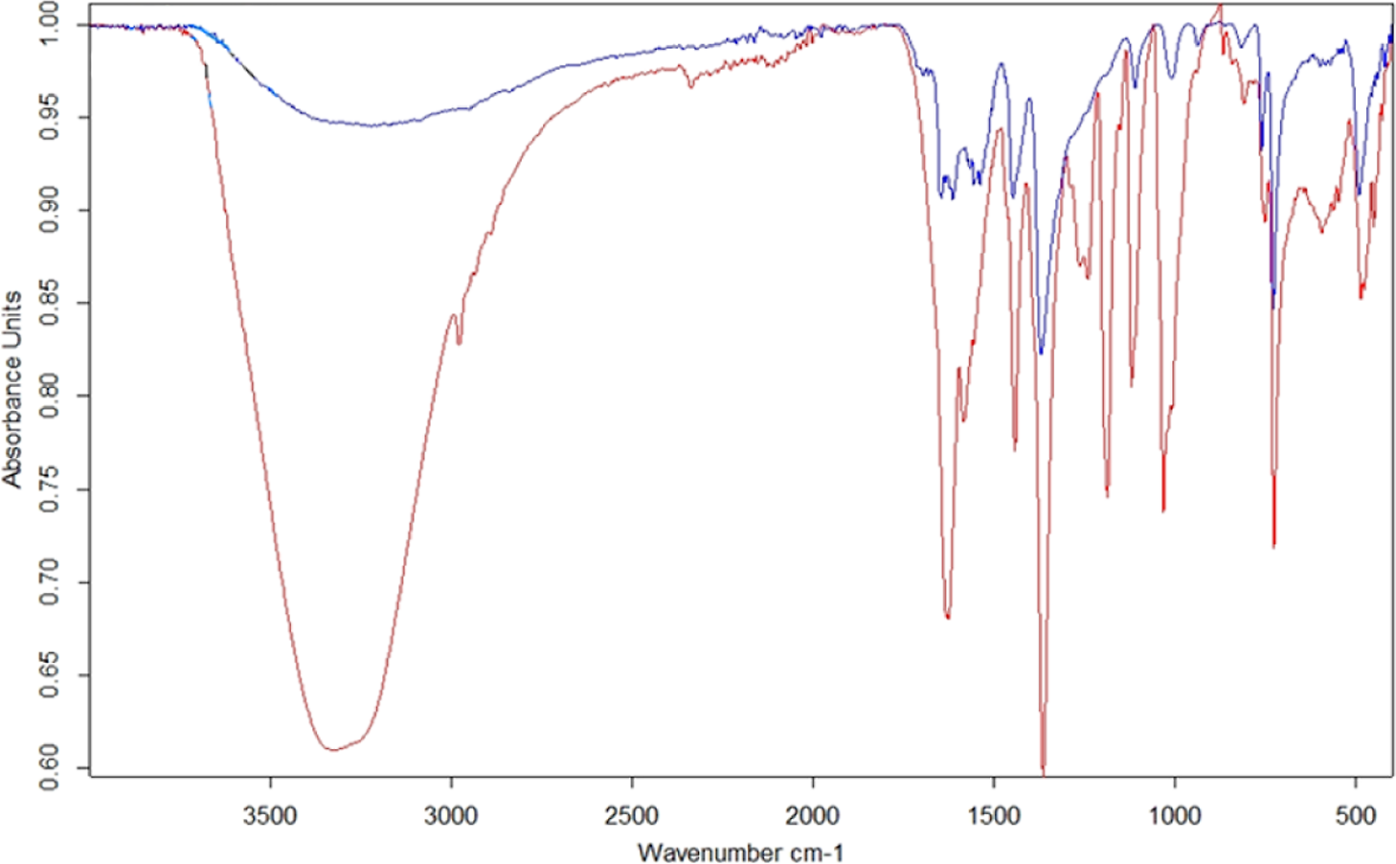
FTIR spectra of activated HKUST-1 (blue line) and 1:1 HKUST-1/KEX (red line).

In the FTIR spectrum of HKUST-1 (see Figures 7, and S57, S58), all the characteristic peaks were observed: Cu–O stretch at 728 cm^–1^, C–O stretch at 1370 cm^–1^, C=O and C=C stretches at 1539–1695 cm^–1^ and O–H stretch at 3206 cm^–1^. The FTIR spectrum of HKUST-1/KEX 1:1 proof of KEX uptake into the MOF was observed. In addition to the peaks arising from HKUST-1, characteristic peaks of KEX were seen. C–H stretch at 2979 and 1442 cm^–1^, C–O–C bend at 1120–1240 cm^–1^, C=S stretch at 1032 cm^–1^, and C–S stretch at 595 cm^–1^. Also, the peaks from HKUST-1 C=O, C=C and Cu–O stretches had larger intensities in the 1:1 sample than in HKUST-1 alone (Figure 7). Similar results were obtained with both of the PSM forms of HKUST-1 (see Figures S59–S64 in the Supporting Information).

In the case of MIL-100(Fe) (both solvothermally and mechanochemically synthetized), MIL-96(Al) and UiO-66 the FTIR spectra (see Figures S65–S76) did not reveal the uptake of KEX as clearly as the HKUST-1 and it is PSM forms. Instead, all the characteristic peaks were observed for the MOFs in samples with and without KEX. For MIL-100(Fe), from the solvothermal synthesis (Figures S65–S67), Fe–O stretch was observed at 457 cm^–1^, C–O stretch at 1373 cm^–1^, as well as C=O and C=C stretches at 1621–1446 cm^–1^. The FTIR spectrum of mechanochemically synthetized MIL-100(Fe) (Figures S68–S70) Fe–O stretch was observed at 457 cm^–1^, C–O stretch at 1372 cm^–1^, as well as C=O, and C=C stretches at 1614–1446 cm^–1^. MIL-96(Al) (Figures S71–S73) showed peaks at 539 cm^–1^ for Al–O, and 1399 cm^–1^ for C–O and at 1460–1660 cm^–1^ for C=C and C=O stretches. With UiO-66 (Figures S74–S76) peaks, at 500 cm^–1^ for Zr–O, at 1390 cm^–1^ for C–O, and at 1557–1434 cm^–1^ for C=C and C=O stretches.

## Conclusions

HKUST-1 and its modified forms were observed to be the most effective MOFs in the uptake of KEX in this study. Especially, in the concentration range relevant to the potential application regarding purification of mine waste waters in arctic areas, in which the concentration of xanthates in the waste waters is usually below 10 mg/L.^35,61^ In higher concentration of KEX, the MOF network collapses, as can be observed in the all the NMR measurements conducted excluding the HKUST-1 with 4-PA. The other studied MOFs were weaker in the uptake of KEX and followed the order mechanochemical MIL-100(Fe) > MIL-100/96(Al) > solvothermal MIL-100(Fe) > UiO-66.

When regarding the stability of the MOFs studied, in the current study activated HKUST-1 with 4-PA was the most effective MOF. Instead, when looking at the uptake of KEX only, the activated HKUST-1 was the most effective. When considering the results obtained with solid-state NMR, PXRD, and FTIR, it is confirmed that the structure of the studied MOFs stays intact in the low concentrations of KEX. This is promising for the future application in the mine wastewater purification. In the future, a more detailed study on the factors affecting the stability of the MOFs and the uptake of xanthates are to be conducted, as well as tests with real mine wastewaters. In addition, 3D-printed objects of the most effective MOFs found in this study are to be tested regarding the uptake of other xanthates as well. With the 3D-printed objects more accurate NMR titrations, reusability tests as well as thermodynamic measurements are possible.

## Supporting Information Available

Details of the syntheses and measurement methods; instruments and sample preparation; PXRD analysis; ^1^H NMR-titration spectra; and FTIR spectra (PDF)
