# Supplementary material for: Uptake of Ethyl Xanthate to Metal Organic Frameworks
Source: ACS Omega. 2023 Sep 14;8(38):35044–53. doi: 10.1021/acsomega.3c04539 (PMC10536054; doi:10.1021/acsomega.3c04539)
Supplement: Supplementary file 1 — ao3c04539_si_001.pdf [file ao3c04539_si_001.pdf]

Uptake of Ethyl Xanthate to Metal Organic Frameworks

Supporting Information

Riikka Kuosmanen*, Elina Sievänen, and Manu Lahtinen*

Department of Chemistry, University of Jyväskylä, P.O. Box 35, 40014 Jyväskylä, FINLAND

E-mail: riikka.t.kuosmanen@jyu.fi, elina.i.sievanen@jyu.fi, manu.k.lahtinen@jyu.fi

Table of Contents

Synthesis 2

PXRD 3

Pawley fit results 4

NMR-titrations 13

Solid state NMR 35

FTIR 36

References 46

Synthesis

MOFs studied were synthetized following methods found from literature. If MOFs were needed in larger amounts, the syntheses were upscaled by multiplying the amounts of reagents. Reagents were purchased from Aldrich, FluoroChem, VWR, Strem Chemicals, and Acros Organics. All reagents and solvents were used as received.

Activation of the MOFs was conducted in vacuum at 90 °C overnight.

HKUST-1 and its modified forms

HKUST-1 was synthetized as described in ref. [1] but slightly modified as the synthesis was conducted in higher temperature. 1.8 mmol (435 mg) of $Cu(NO_3)_2{\cdot}3H_2O$ was dissolved in 6 mL of deionized water. 1.0 mmol (221 mg) of H3btc was dissolved in 6 mL of ethanol (99 %, Altia). Solutions were combined and transferred into 25 mL Teflon lined autoclave and kept in 120 °C for 24h. After cooling to room temperature within 6 h, the turquoise product was washed with deionized water three times. The nature of the MOF was verified with PXRD.

Post-synthetic modification was conducted with vacuum dried HKUST-1 following the procedure in refs. [2] and [3].

MIL-100 (Fe)

MIL-100(Fe) was synthetized by mixing 1:1 $Fe(NO_3)_2{\cdot}9H_2O$:H3btc and adding water as a solvent. When a 25 mL Teflon lined autoclave was used, 0.567 mmol of each reagent (119.1 mg of H3btc and 229.1 mg of $Fe(NO_3)_2{\cdot}9H_2O$) and 17 mL of deionized water were used. The autoclave was kept at 200 °C for 8 h and cooled to room temperature within 20 h. The product was washed with EtOH, DMF and deionized water and dried in air. The synthesis method was adopted from ref. [4]. The nature of the product was confirmed with PXRD.

Mechanochemical MIL-100(Fe)

Same reagents were used in the mechanochemical synthesis as in the solvothermal synthesis of MIL-100(Fe) in 1.5:1 (H3btc:iron salt) molar ratio. The synthesis method was adopted from ref. [5]. Both solid reagents (630 mg of H3btc and 808 mg of $Fe(NO_3)_2{\cdot}9H_2O$) were put into an agate mortar and ground for 10 min. During the grinding the reaction mixture changed from white and fluffy to pale yellow and sticky. Reaction mixture was transferred to 25 ml Teflon lined autoclave and kept at 160 °C for 4 h, followed by cooling to room temperature in 14 h. Red orange product was washed once with deionized water and twice with EtOH.

MIL-100(Al)

MIL-100(Al) was synthesized according to the instruction given in ref. [6]. H3btc (2.40 mmol, 505 mg) and $Al(NO_3)_2{\cdot}9H_2O$ (4.23 mmol, 900 mg) were weighed to beakers, followed by addition of 5 mL deionized water. After 10 min stirring, solutions were combined and stirring continued at room

temperature for 1 h. 0.2 mL of glacial acetic acid was added dropwise to the reaction mixture and stirring continued for 5 min. Reaction mixture was transferred to a 25 mL Teflon lined autoclave. Autoclave was placed into an oven and temperature was slowly increased to 210 °C (40 °C/h). The temperature was held at 210 °C for 30 min. Autoclave was cooled rapidly by placing it to an ice bath. As soon as it was possible, the autoclave was opened, and the Teflon liner was allowed to cool down to room temperature in a fume hood. Pale yellow product was washed three times with MeOH and dried in air. The product was confirmed to be MIL-100(Al) with PXRD.

Due to the rapid cooling in the ice bath, some of the product escaped from the Teflon lined inner container. Because of this, the following syntheses were cooled in fume hood after 30 min at 210 °C in the oven. The resulting products were a mixture of MIL-100(Al) and MIL-96(Al) according the PXRD patterns.

UiO-66

UiO-66 was synthetized with the same method as in ref. [7]. 0.13 mmol of H2bdc (21.6 mg) and $ZrCl_4$ (30.3 mg) were weighed into a beaker followed by addition of DMF (13.6 mL). The mixture was then stirred and 1.3 mmol of glacial acetic acid was added. After this the reaction mixture was 20 min in an ultrasound bath. Reaction mixture was transferred to a 25 mL Teflon-lined autoclave it was held at 120 °C for 24h. After cooling to room temperature within 10 h, the product was washed three times with DMF and MeOH. Product was dried in air. The identity of the product was confirmed with PXRD.

PXRD

Measurements were conducted for pristine and activated MOFs as well as 1:1 ratio (MOF:KEX) samples. Samples were prepared similarly to the solid-state NMR measurements. 10 mg of MOF in question was weighed and appropriate amount of KEX in ultrapure water was added. After 40 min the sample was either filtered with glass sinther or centrifugated, after which the sample was measured.

The room- temperature powder X-ray diffraction (PXRD) measurements were made by PANalytical X´Pert PRO diffractometer using Cu K_α radiation (λ = 1.5418 Å; 45 kV, 40 mA). For a routine PXRD experiments lightly hand-ground powder samples were prepared on zero–background signal generating Si-plate using petrolatum jelly as an adhesive. Diffraction intensities were recorded from a spinning sample. Diffraction data were acquired by an X´Celerator detector using 2θ range of 3−60° with step size of 0.017° and counting time of 50 s per step. Data processing and Pawley fits were performed with the program X´pert HighScore Plus (v. 4.9). [8] The unit cell parameters of the powder samples were refined by Pawley analysis using the corresponding single crystal structure parameters retrieved from the Cambridge Structrural Database (CSD). [9] Variables for the fits were as follows: zero-offset, polynomial background, sample displacement, unit cell parameters and peak profile parameters including peak width, shape, and asymmetry.

Pawley fit results

HKUST-1 MOFs

Table S1. Crystallographic data of **HKUST-1**, **HKUST-1_activated**, **HKUST-1_3PA** and **HKUST-1_4PA** determined by whole pattern Pawley fits compared with corresponding single crystal data (SCXRD).

Parameters	SCXRD	PXRD	PXRD	PXRD	PXRD	SCXRD
Compound	FIQCEN[10]	**HKUST-1**	**HKUST-1_activated**	**HKUST-1_3PA**	**HKUST-1_4PA**	BODPAN[11]
Temp (°C)	22	22	22	22	22	22
Crystal system	cubic	cubic	cubic	cubic	cubic	cubic
Space group	*Fm-3m*	*Fm-3m*	*Fm-3m*	*Fm-3c*	*Fm-3c*	*Fm-3c*
a /Å	26.343(5)	26.325(2)	26.326(8)	52.63(3)	52.535(6)	52.5078(17)
b /Å	26.343(5)	26.325(2)	26.326(8)	52.63(3)	52.535(6)	52.5078(17)
c /Å	26.343(5)	26.325(2)	26.326(8)	52.63(3)	52.535(6)	52.5078(17)
α /°	90	90	90	90	90	90
β /°	90	90	90	90	90	90
γ /°	90	90	90	90	90	90
V/Å^3	18280	18242	18247	145794	144996	144768
R_p		0.0342	0.0443	0.0283	0.0354	
R_{wp}		0.0407	0.0507	0.0323	0.0439	
GOF		1.5017	1.85135	1.88115	2.511	

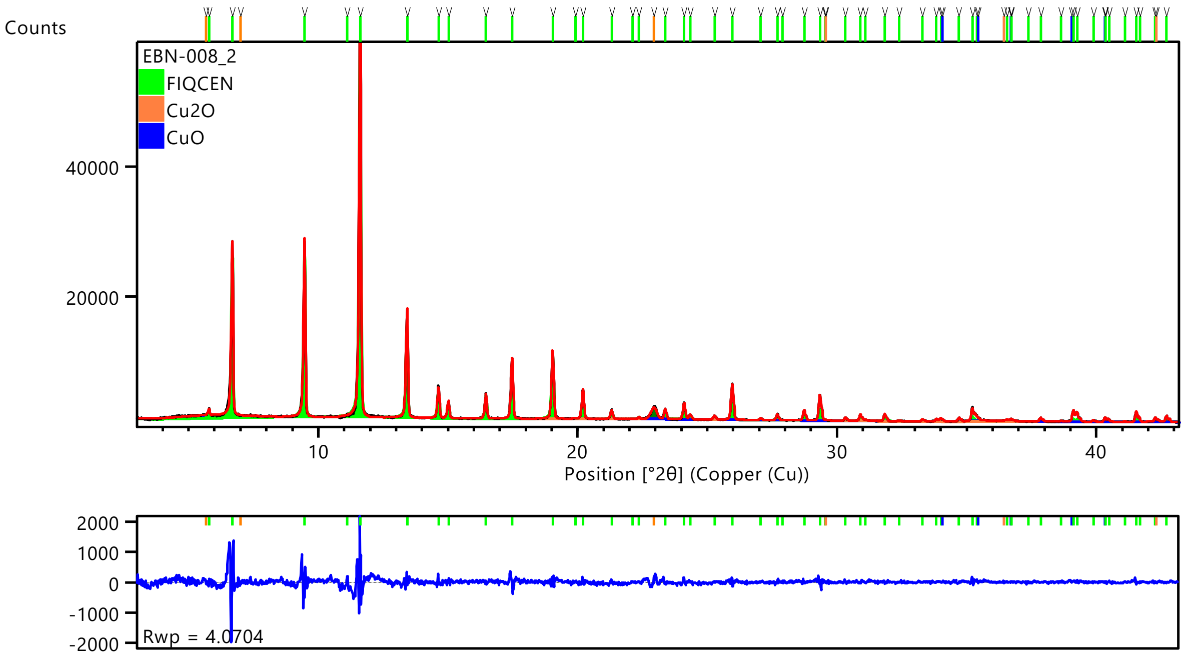

Figure S1. Pawley refinement plot of **HKUST-1**. Experimental pattern is shown in black and refined profile in red, whereas green colored markers on top correspond to characteristic Bragg peak positions of the main component (CSD entry: FIQCEN). Whereas the blue and orange markers indicate peak positions of residual copper oxide phases. Difference plot of experimental vs. refined profile is shown below in blue color.

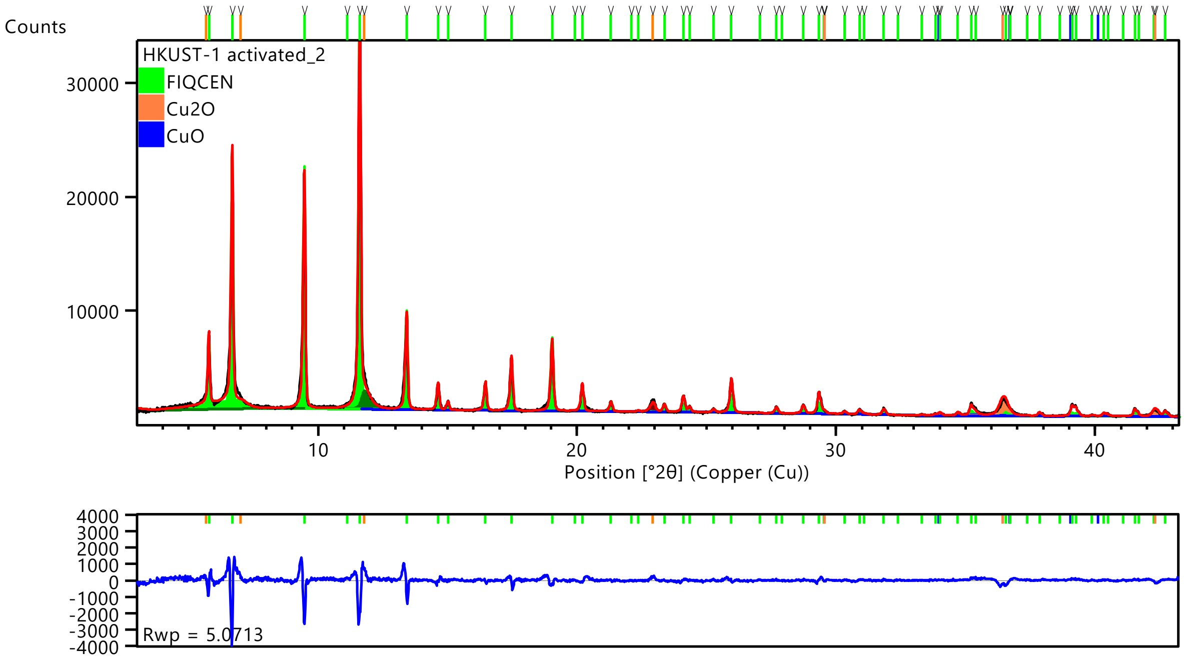

Figure S2. Pawley refinement plot of **HKUST-1_activated.** Experimental pattern is shown in black and refined profile in red, whereas green colored markers on top correspond to characteristic Bragg peak positions of the main component (CSD entry: FIQCEN). Whereas the blue and orange markers indicate peak positions of residual copper oxide phases. Difference plot of experimental vs. refined profile is shown below in blue color.

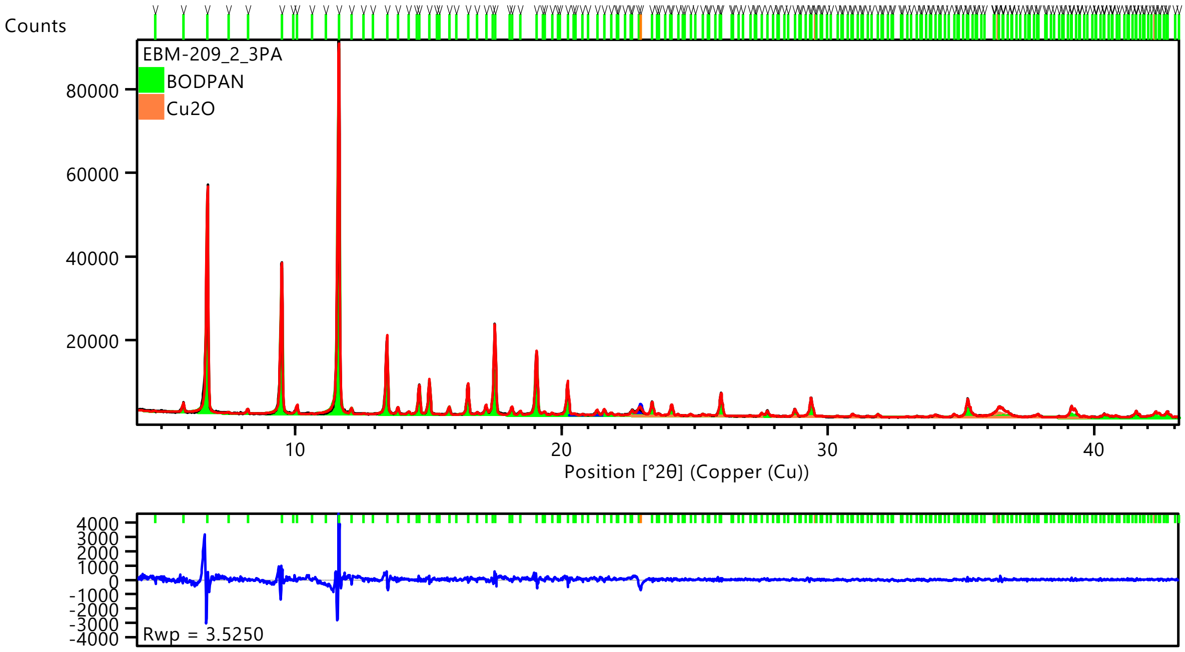

Figure S3. Pawley refinement plot of **HKUST-1_3PA**. Experimental pattern is shown in black and refined profile in red, whereas green colored markers on top correspond to characteristic Bragg peak positions of the of the main component (CSD entry: BODPAN). Whereas the orange markers indicate peak positions of residual copper oxide phase. Difference plot of experimental vs. refined profile is shown below in blue color.

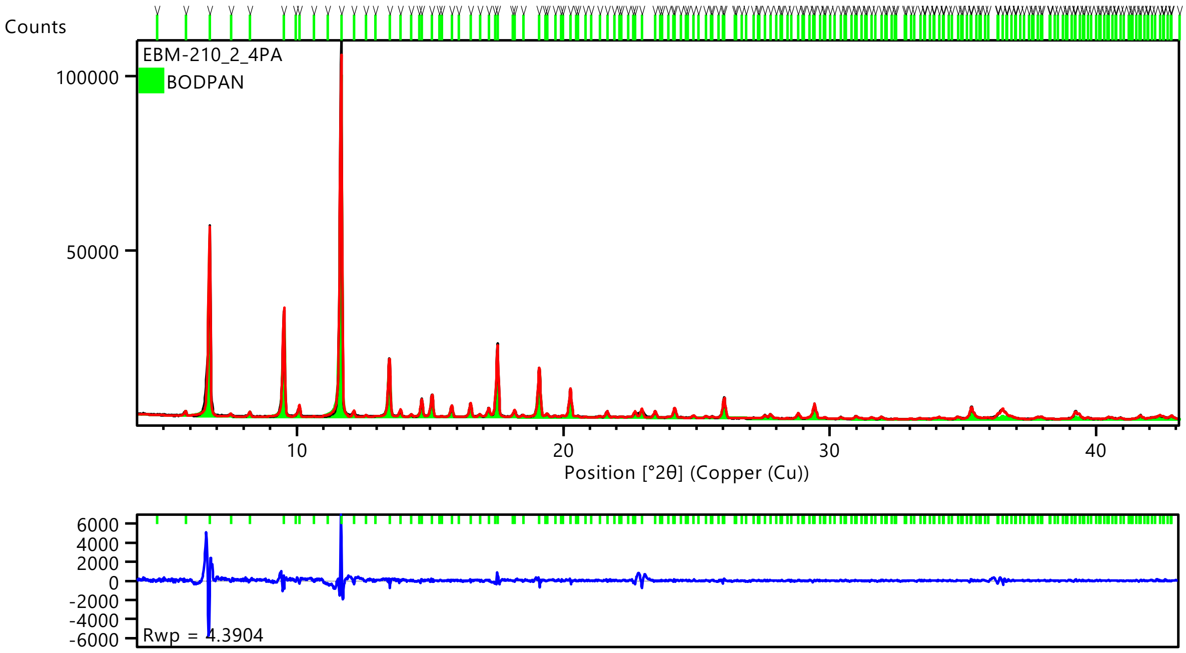

Figure S4. Pawley refinement plot of **HKUST-1_4PA**. Experimental pattern is shown in black and refined profile in red. Characteristic Bragg peak positions of the main component (CSD entry: BODPAN) are shown by vertical markers on top. Difference plot of experimental vs. refined profile is shown below.

MIL-100 MOFs

Table S2. Crystallographic data of **MIL-100(Fe)**, **MIL-100(Fe)-mechano**, **MIL-100(Fe)-activated** and MIL-**100(Fe)-mechano-activated** determined by whole pattern Pawley fits compared with corresponding single crystal data (SCXRD).

Parameters	PXRD*	PXRD	PXRD	PXRD	SCXRD
Compound	**MIL-100(Fe)**	**MIL-100(Fe)-mechano**	**MIL-100(Fe)-activated**	**MIL-100(Fe)-mechano-activated**	CIGXIA[12]
Temperature (°C)	22	22	22	22	22
Crystal system	cubic	cubic	cubic	cubic	cubic
Space group	*Fd*-3*m*	*Fd*-3*m*	*Fd*-3*m*	*Fd*-3*m*	*Fd*-3*m*
a /Å	73.4(3)	73.4(3)	73.4(3)	73.3(2)	73.34019
b /Å	73.4(3)	73.4(3)	73.4(3)	73.3(2)	73.34019
c /Å	73.4(3)	73.4(3)	73.4(3)	73.3(2)	73.34019
α /°	90	90	90	90	90
β /°	90	90	90	90	90
γ /°	90	90	90	90	90
V/Å^3	394810	394677	394772	394545	394481
$R_{prof.}$	0.0333	0.0282	0.0108	0.0214	
$R_{w\text{-}prof}$	0.0402	0.0311	0.1545	0.0225	
GOF	1.324	1.452	1.221	1.549	

* Unit cell parameters for the trace components have not been presented.

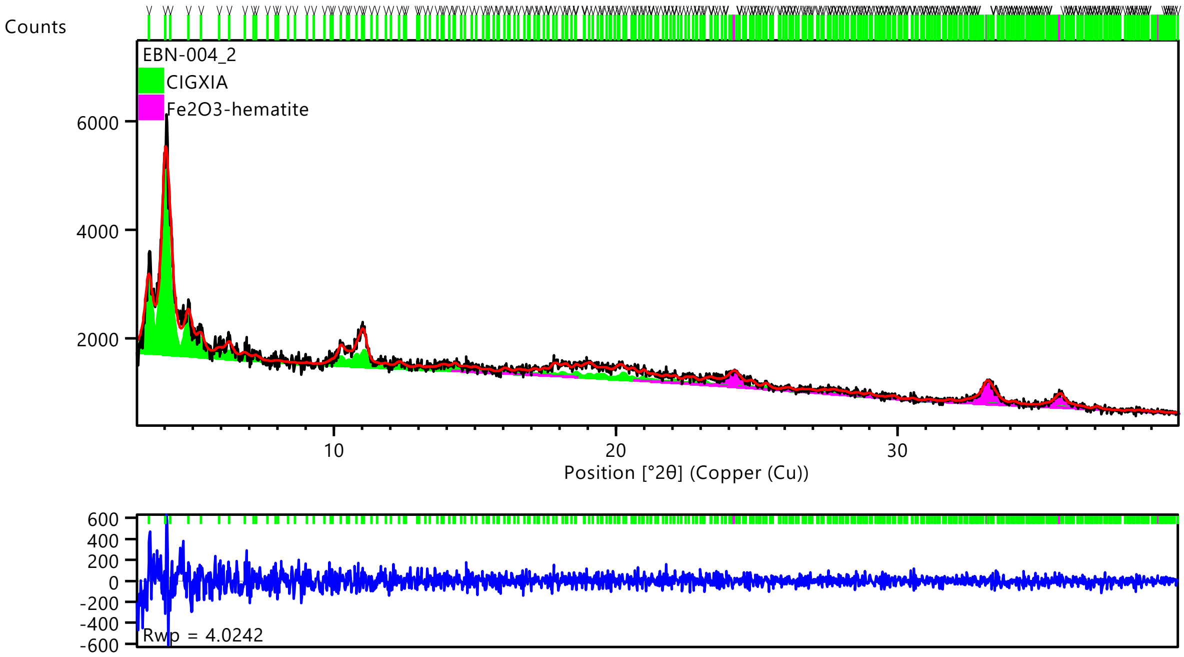

Figure S5. Pawley refinement plot of **MIL-100(Fe)**. Experimental pattern is shown in black and refined profile in red. Characteristic Bragg peak positions of the main component MIL-100(Fe) (green: CSD entry CIGXIA), and trace impurity phase hematite Fe_2O_3 (pink: PDF entry 01-089-0597) are shown by vertical markers on top. Difference plot of experimental vs. refined profile is shown below.

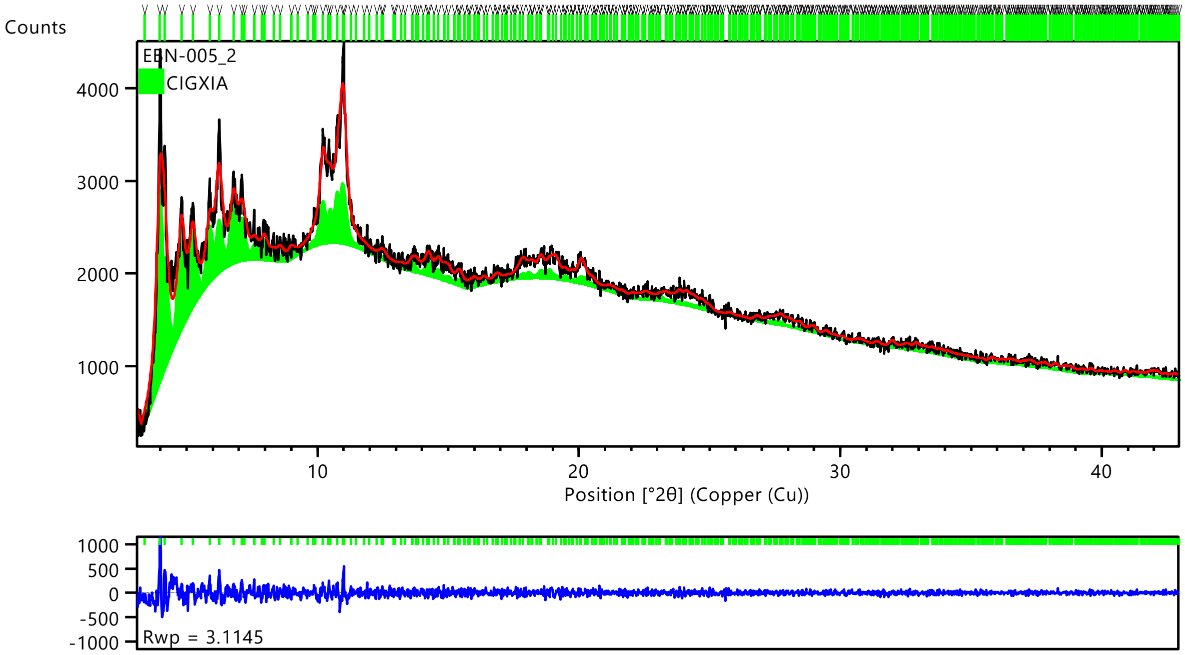

Figure S6. Pawley refinement plot of **MIL-100(Fe)-mechano**. Experimental pattern is shown in black and refined profile in red. Characteristic Bragg peak positions of the main component MIL-100(Fe) (green: CSD entry CIGXIA). Difference plot of experimental vs. refined profile is shown below.

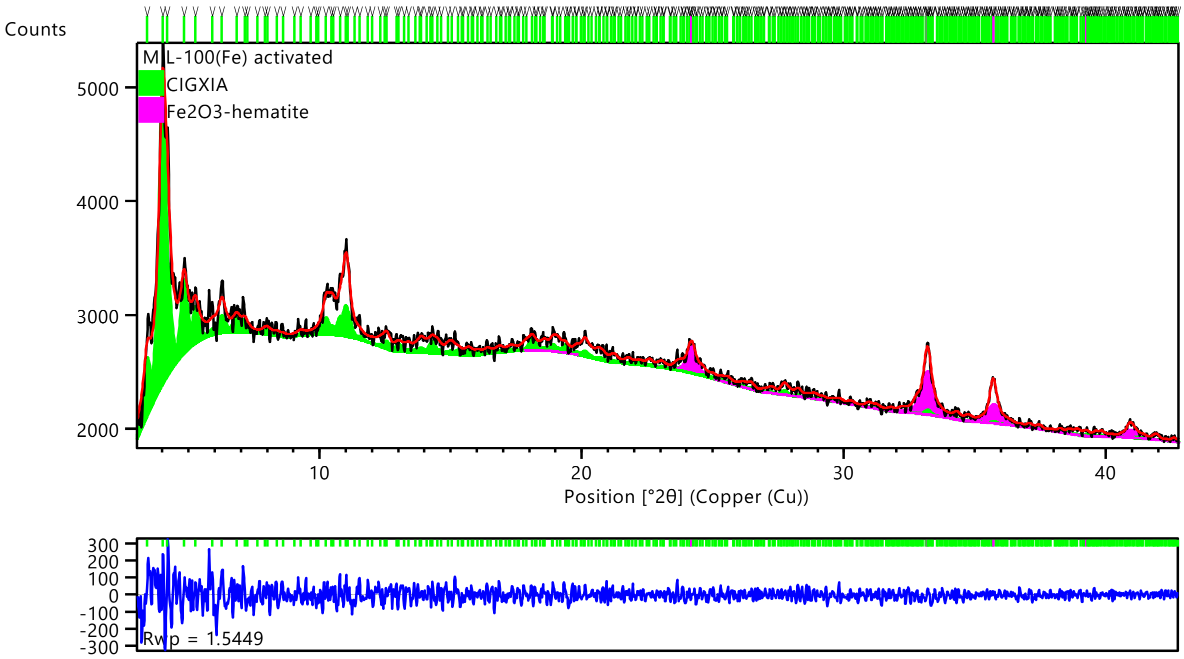

Figure S7. Pawley refinement plot of **MIL-100(Fe)-activated**. Experimental pattern is shown in black and refined profile in red. Characteristic Bragg peak positions of the main component MIL-100(Fe) (green: CSD entry CIGXIA), and trace impurity phase hematite Fe_2O_3 (pink: PDF entry 01-089-0597) are shown by vertical markers on top. Difference plot of experimental vs. refined profile is shown below.

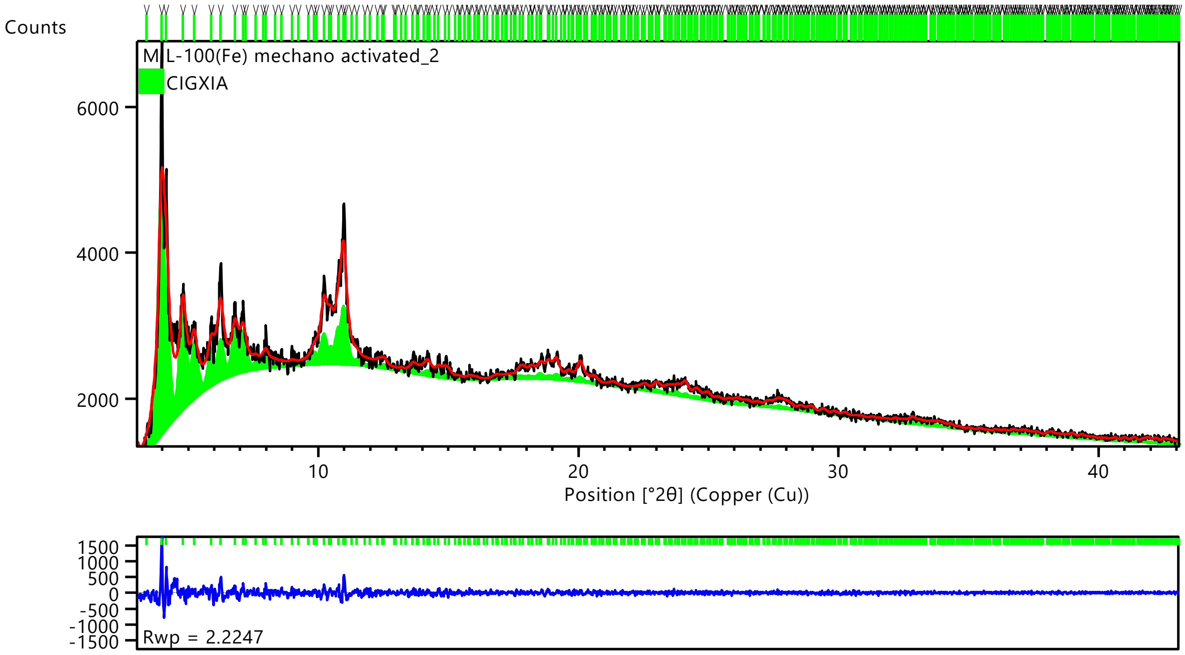

Figure S8. Pawley refinement plot of **MIL-100(Fe)-mechano-activated**. Experimental pattern is shown in black and refined profile in red. Characteristic Bragg peak positions of the main component MIL-100(Fe) (green: CSD entry CIGXIA. Difference plot of experimental vs. refined profile is shown below.

MIL-100/-96(Al) MOFs

Table S3. Crystallographic data of **MIL-100(Al)** and **MIL-100/-96(Al)** determined by whole pattern Pawley fits compared with corresponding single crystal data (SCXRD).

Parameters	PXRD	SCXRD	PXRD		SCXRD
Compound	**MIL-100(Al)**	UDEMEW[13]	**MIL-100/-96(Al)**		WEVYEE[14]
Temperature (°C)	22	-193	22		-173
Crystal system	cubic	cubic	cubic	rhombohedral	rhombohedral
Space group	*Fm*-3*m*	*Fm*-3*m*	*Fm*-3*m*	$P6_3/mmc$	$P6_3/mmc$
a /Å	72.1(4)	72.9057	71.94 (1)	14.328(4)	14.290(2)
b /Å	72.1(4)	72.9057	71.94 (1)	14.328(4)	14.290(2)
c /Å	72.1(4)	72.9057	71.94 (1)	31.311(6)	31.300(6)
α /°	90	90	90	90	90
β /°	90	90	90	90	90
γ /°	90	90	90	120	120
V/Å^3	374227	387511	372248	5566.7	5535.28
$R_{prof.}$	0.0614		0.0412		
$R_{w\text{-}prof}$	0.0699		0.0457		
GOF	2.958		1.731		

Ref. [13], unit cell of isostructural MIL-100(Cr) was used in comparison.

Table S4. Crystallographic data of **MIL-100/-96(al)**, and **MIL-100/-96(Al)Fe-activated** determined by whole pattern Pawley fits compared with corresponding single crystal data (SCXRD).

Parameters	PXRD		SCXRD	SCXRD
Compound	**MIL-100/-96(Al)-activated**		UDEMEW[13]	WEVYEE[14]
Temperature (°C)	22		-193	-173
Crystal system	cubic	rhombohedral	cubic	rhombohedral
Space group	*Fm*-3*m*	$P6_3/mmc$	*Fm*-3*m*	$P6_3/mmc$
a /Å	71.93(2)	14.333(4)	72.9057	14.290(2)
b /Å	71.93(2)	14.333(4)	72.9057	14.290(2)
c /Å	71.93(2)	31.3(1)	72.9057	31.300(6)
α /°	90	90	90	90
β /°	90	90	90	90
γ /°	90	120	90	120
V/Å^3	372112	5573.4	387511	5535.28
$R_{prof.}$	0.0418			
$R_{w\text{-}prof}$	0.0455			
GOF	1.744			

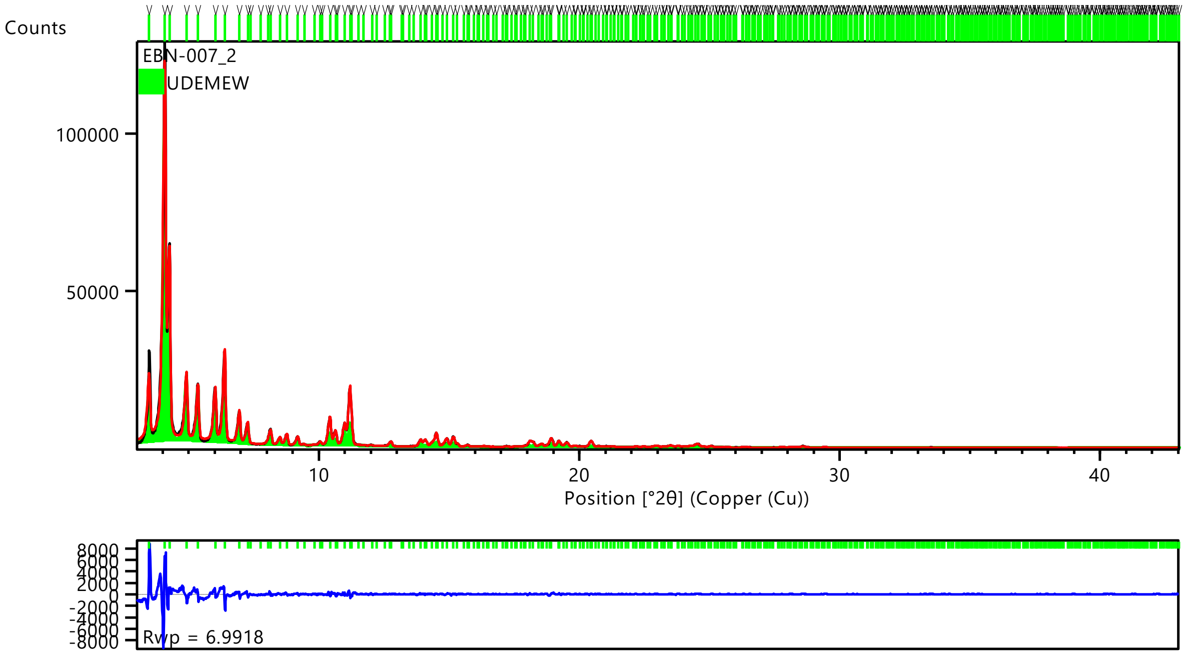

Figure S9. Pawley refinement plot of **MIL-100(Al)**. Experimental pattern is shown in black and refined profile in red. Characteristic Bragg peak positions of the main components MIL-100(Al) are shown by vertical markers on top (green: CSD entry UDEMEW). Difference plot of experimental vs. refined profile is shown below. UDEMEW corresponds to structure analogue MIL-100(Cr).

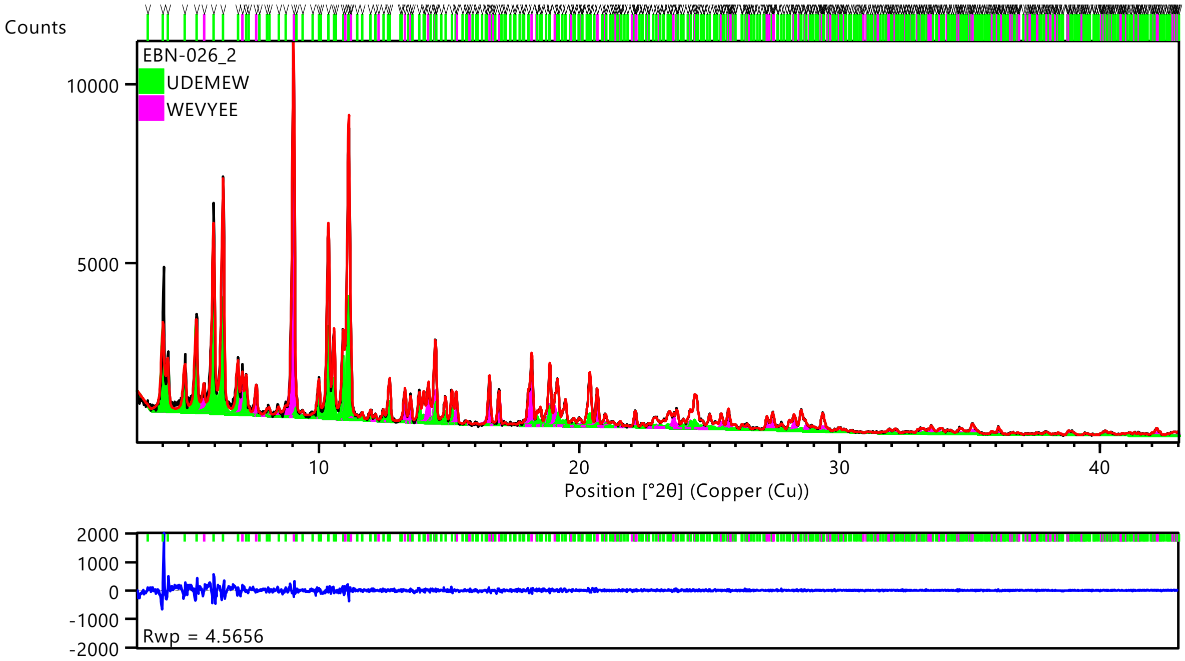

Figure S10. Pawley refinement plot of **MIL-100(Al) + MIL-96(Al)**. Experimental pattern is shown in black and refined profile in red. Characteristic Bragg peak positions of the main components MIL-100(Al) and MIL-96(Al) (green and pink: CSD entries UDEMEW and WEVYEE, respectively) are shown by vertical markers on top. Difference plot of experimental vs. refined profile is shown below. UDEMEW corresponds to structure analogue MIL-100(Cr).

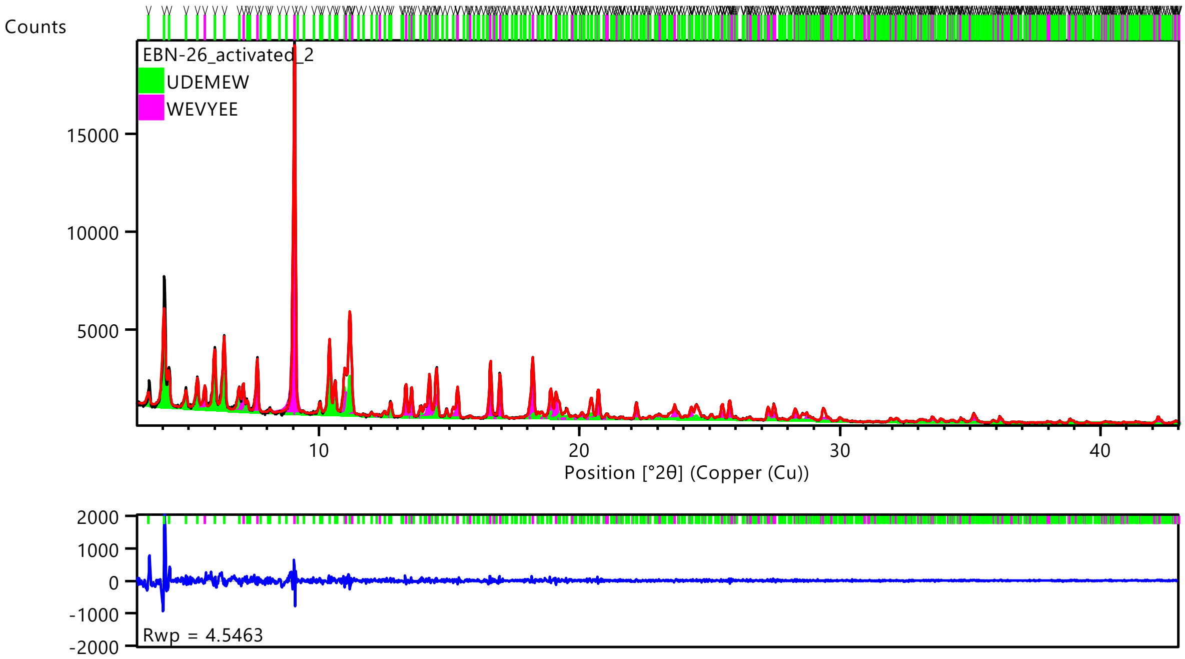

Figure S11. Pawley refinement plot of **MIL-100/-96(Al)-activated**. Experimental pattern is shown in black and refined profile in red. Characteristic Bragg peak positions of the main components MIL-100(Al) and MIL-96(Al) (green and pink: CSD entries UDEMEW and WEVYEE, respectively) are shown by vertical markers on top. Difference plot of experimental vs. refined profile is shown below. UDEMEW corresponds to structure analogue MIL-100(Cr).

UIO-66 based MOFs

Table S5. Crystallographic data of **UiO-66** and **UiO-66-activated** determined by whole pattern Pawley fits compared with corresponding single crystal data (SCXRD).

Parameters	PXRD	PXRD	SCXRD
Compound	**UiO-66**	**UiO-66-activated**	RUBTAK06[15]
Temperature (K)	22	22	-173
Crystal system	cubic	cubic	cubic
Space group	*Fm*-3*m*	*Fm*-3*m*	*Fm*-3*m*
a /Å	20.801(3)	20.793(2)	20.7366(9)
b /Å	20.801(3)	20.793(2)	20.7366(9)
c /Å	20.801(3)	20.793(2)	20.7366(9)
α /°	90	90	90
β /°	90	90	90
γ /°	90	90	90
V/Å^3	9000.3	8989.3	8916.87
$R_{prof.}$	0.0314	0.0411	
$R_{w\text{-}prof}$	0.0433	0.0511	
GOF	1.150	1.387	

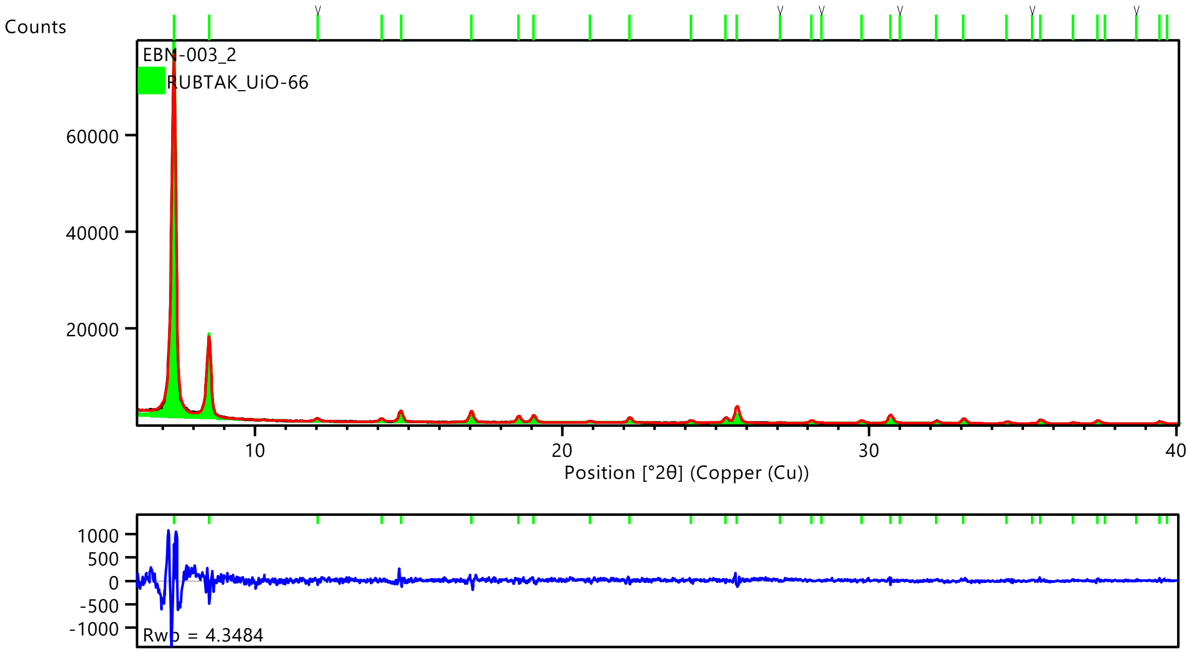

Figure S12. Pawley refinement plot of **UiO-66**. Experimental pattern is shown in black and refined profile in red. Characteristic Bragg peak positions of the main component UiO-66 (green: CSD entry RUBTAK06) is shown by vertical markers on top. Difference plot of experimental vs. refined profile is shown below.

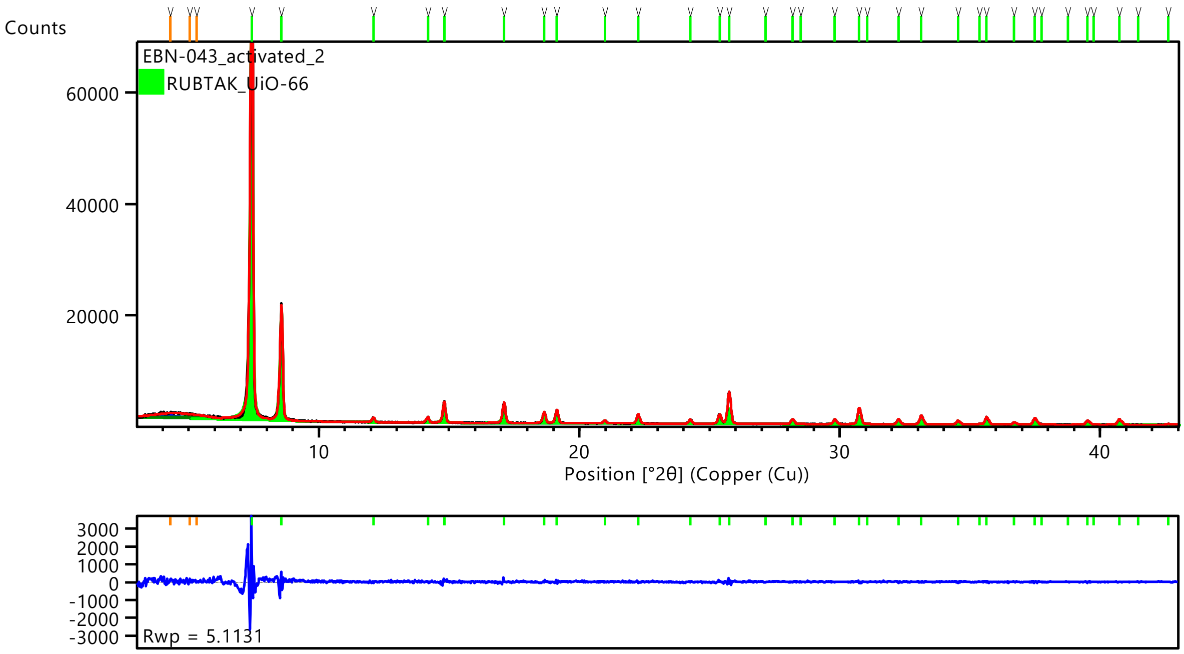

Figure S13. Pawley refinement plot of **UiO-66-activated**. Experimental pattern is shown in black and refined profile in red. Characteristic Bragg peak positions of the main component UiO-66 (green: CSD entry RUBTAK06) is shown by vertical markers on top. Difference plot of experimental vs. refined profile is shown below.

NMR-titrations

NMR-titrations were conducted with Bruker Avance DRX 500 MHz spectrometer. The spectrometer was equipped with Prodigy Cryoprobe operating at 500.17 MHz in ^{1}H experiments.

All measurements were conducted in D_2O. 5 mg of MOF in question was suspended into 600 µL of D_2O in an NMR-tube with two drops of benzene, and appropriate amount of KEX was dissolved in 1 mL of D_2O in a test tube. The ratio of MOF and KEX was 1:1 (with respect of moles). During the titration experiment, KEX was added 0.2 equivalents at a time in 5 min intervals until titration was complete. The titration was continued until the signal of the aromatic protons of the benzene was appr. 1:1 with respect of an aromatic signal observed due to the MOF. In some cases, there were no aromatic proton signal observed from the MOF, thus titration was continued until no significant changes were seen in the spectra.

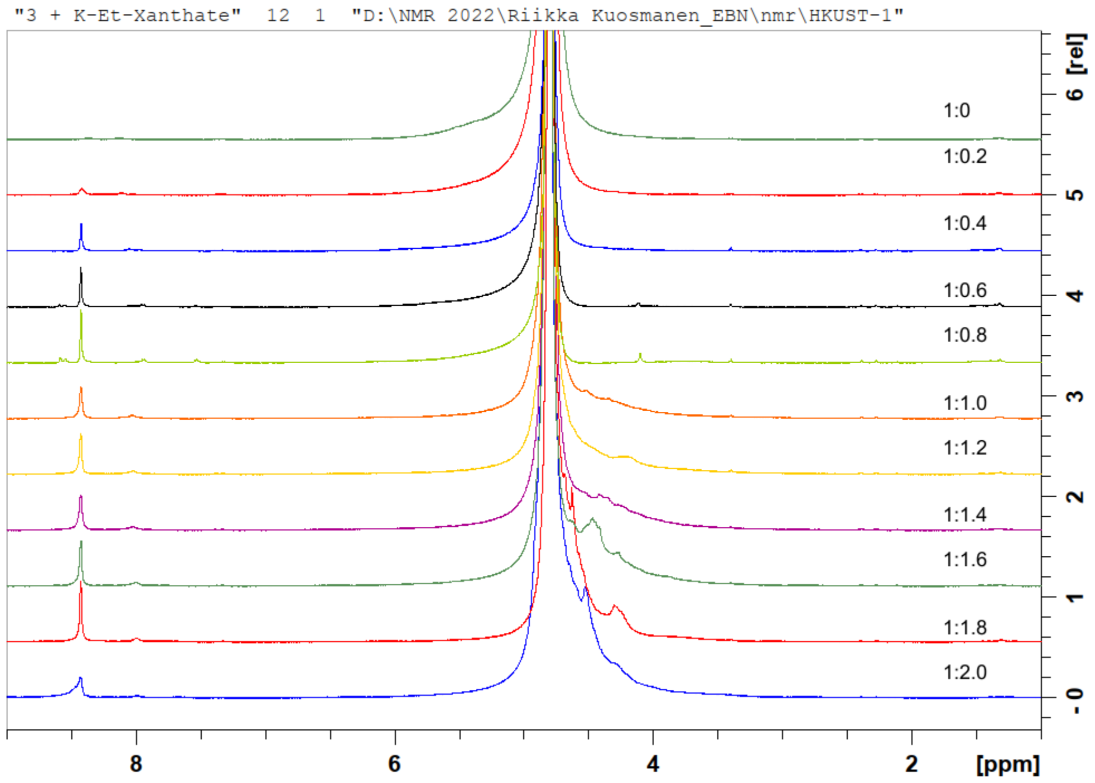

Figure S14. Whole titration of pristine HKUST-1 modified with 3-picolylamine.

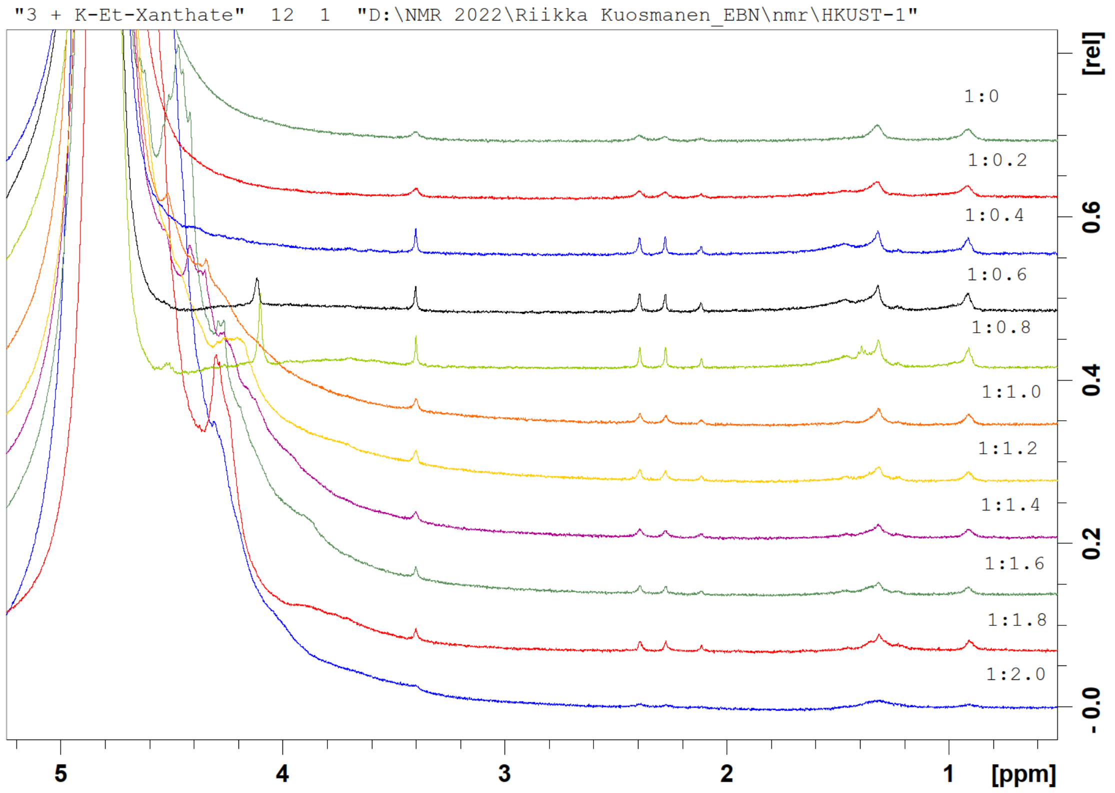

Figure S15. Titration of pristine HKUST-1 modified with 3-picolylamine, aliphatic region.

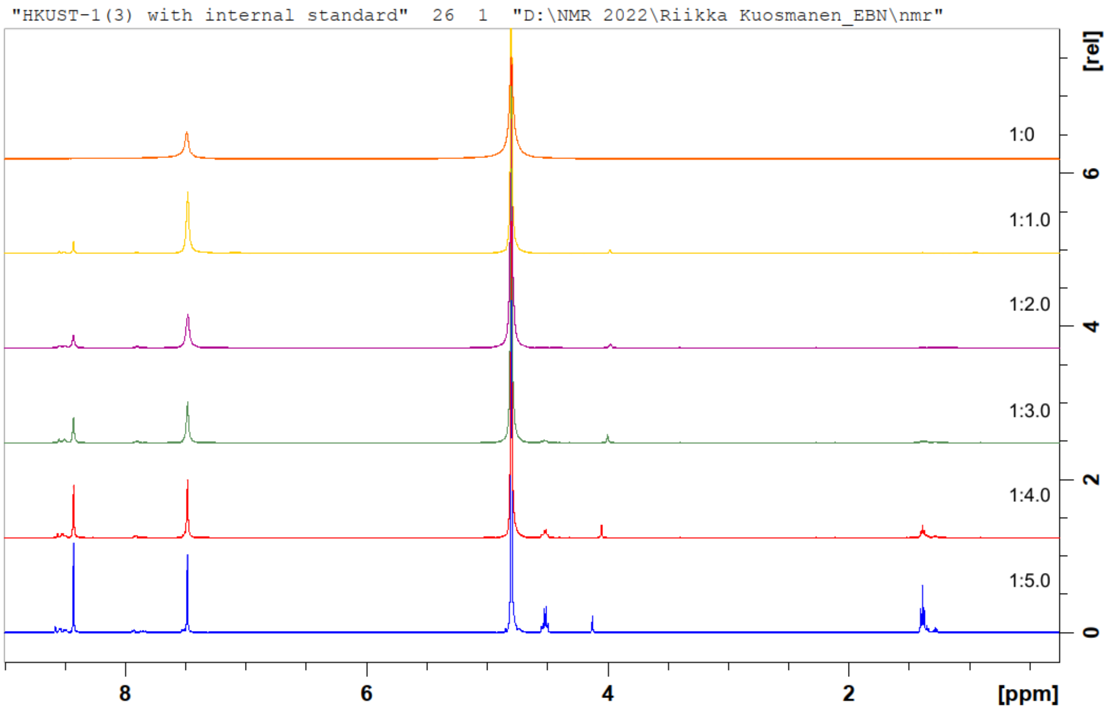

Figure S16. Whole titration of HKUST-1 modified with 3-picolylamine, with internal standard.

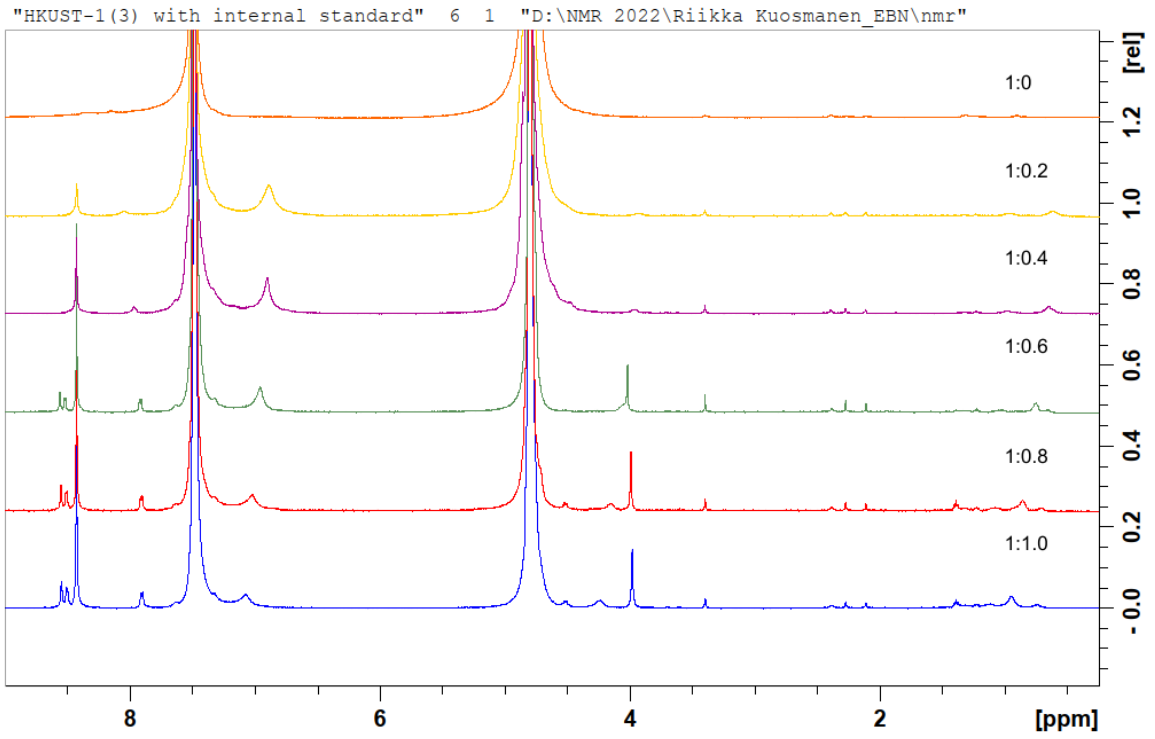

Figure S17. The beginning of titration of HKUST-1 modified with 3-picolylamine, with internal standard.

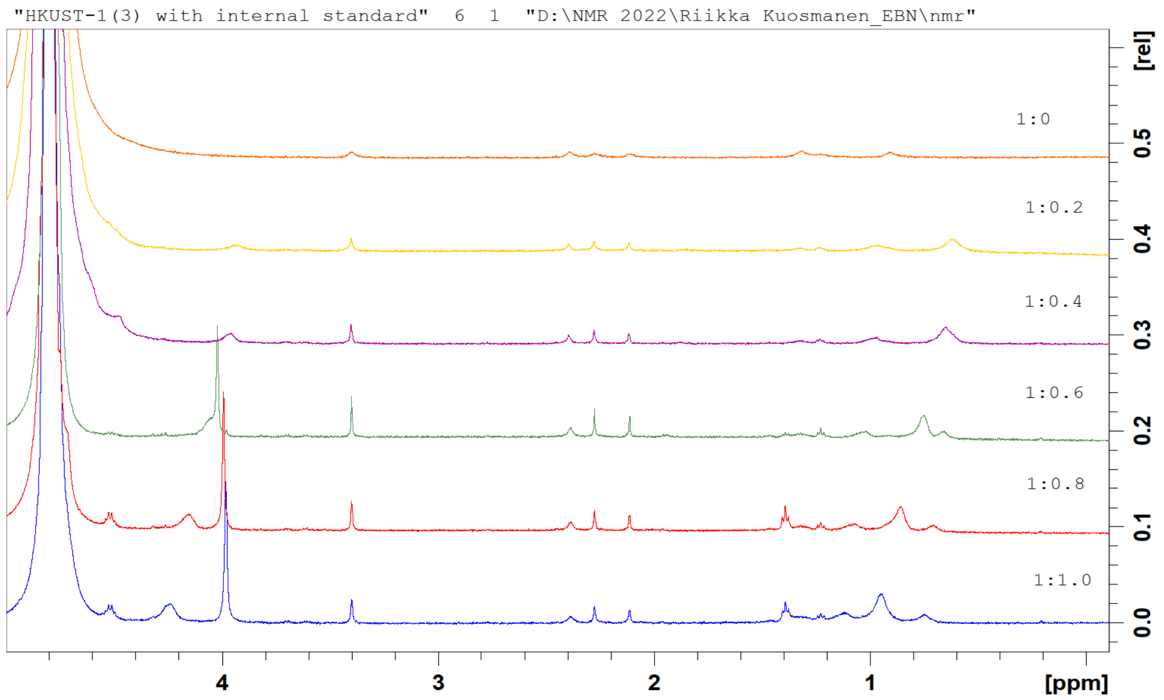

Figure S18. The beginning of titration of HKUST-1 modified with 3-picolylamine, with internal standard, from aliphatic region.

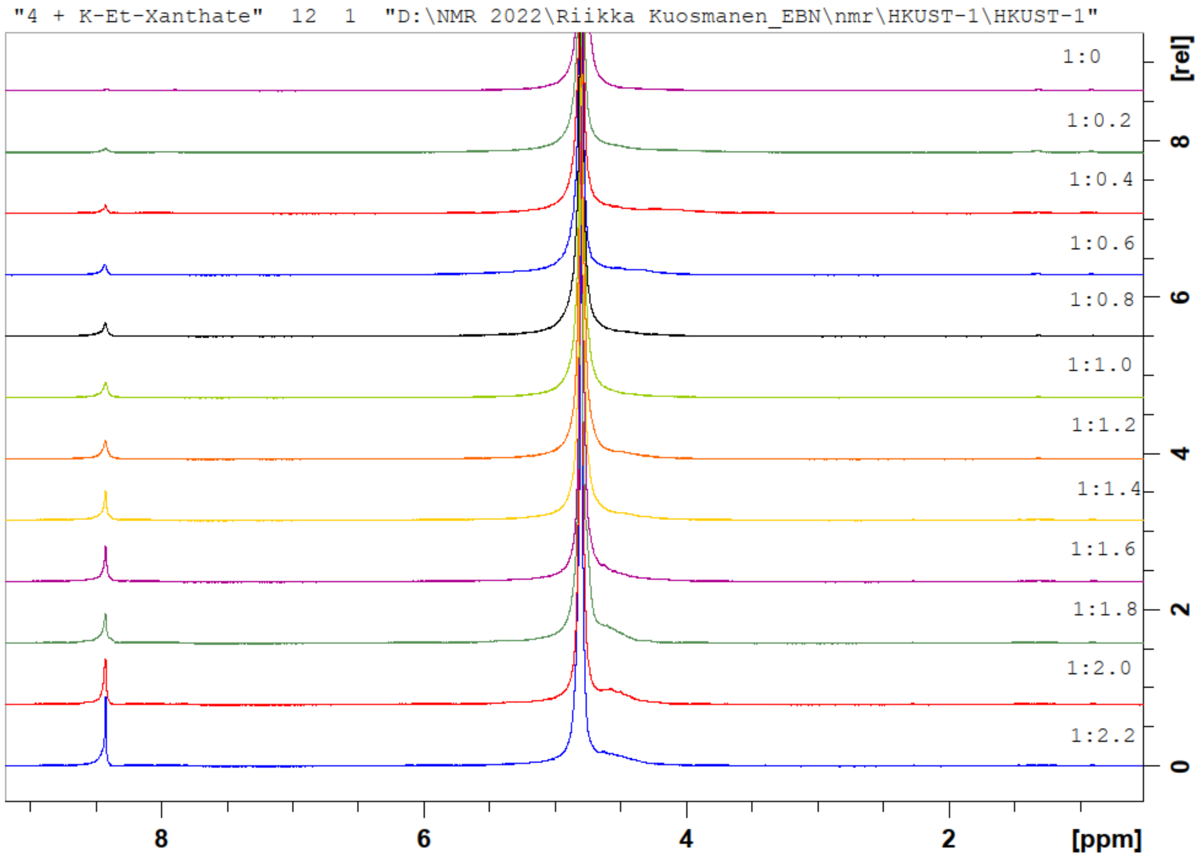

Figure S19. Whole titration of HKUST-1 modified with 4-picolylamine.

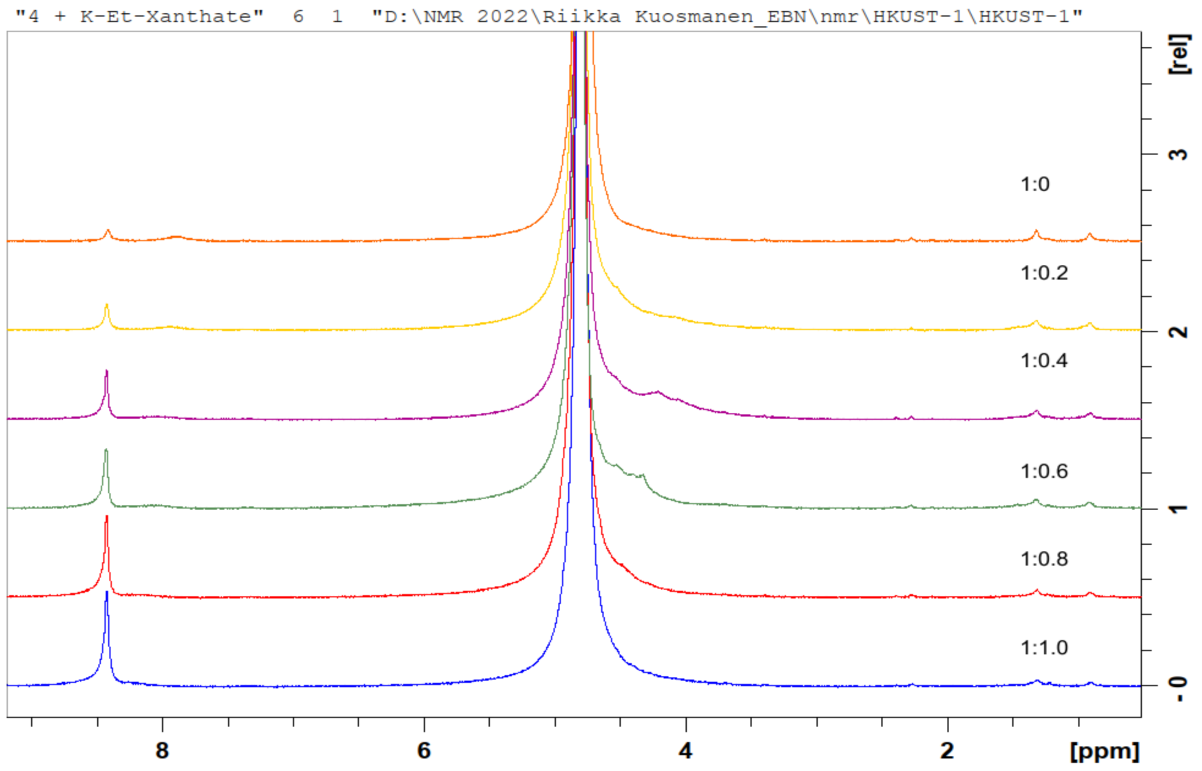

Figure S20. The beginning of titration of HKUST-1 modified with 4-picolylamine.

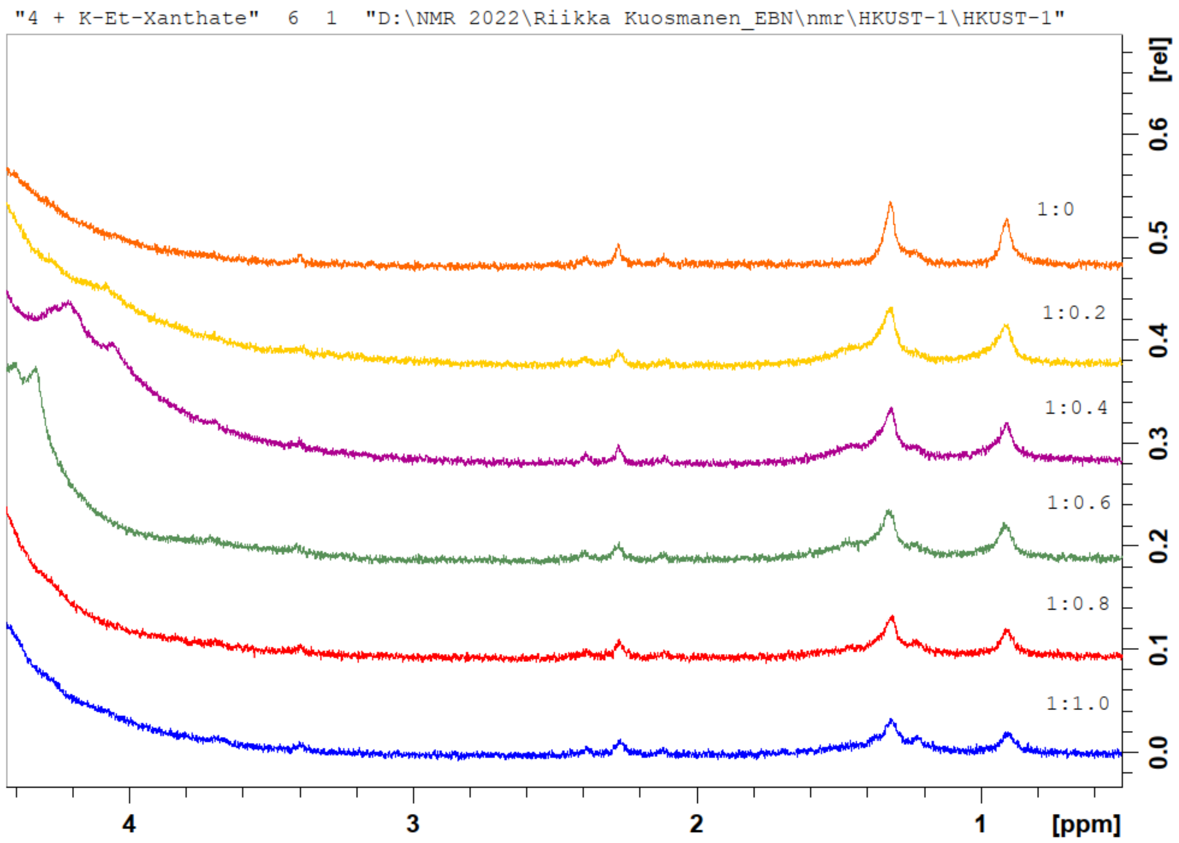

Figure S21. The beginning of titration of HKUST-1 modified with 4-picolylamine, aliphatic region.

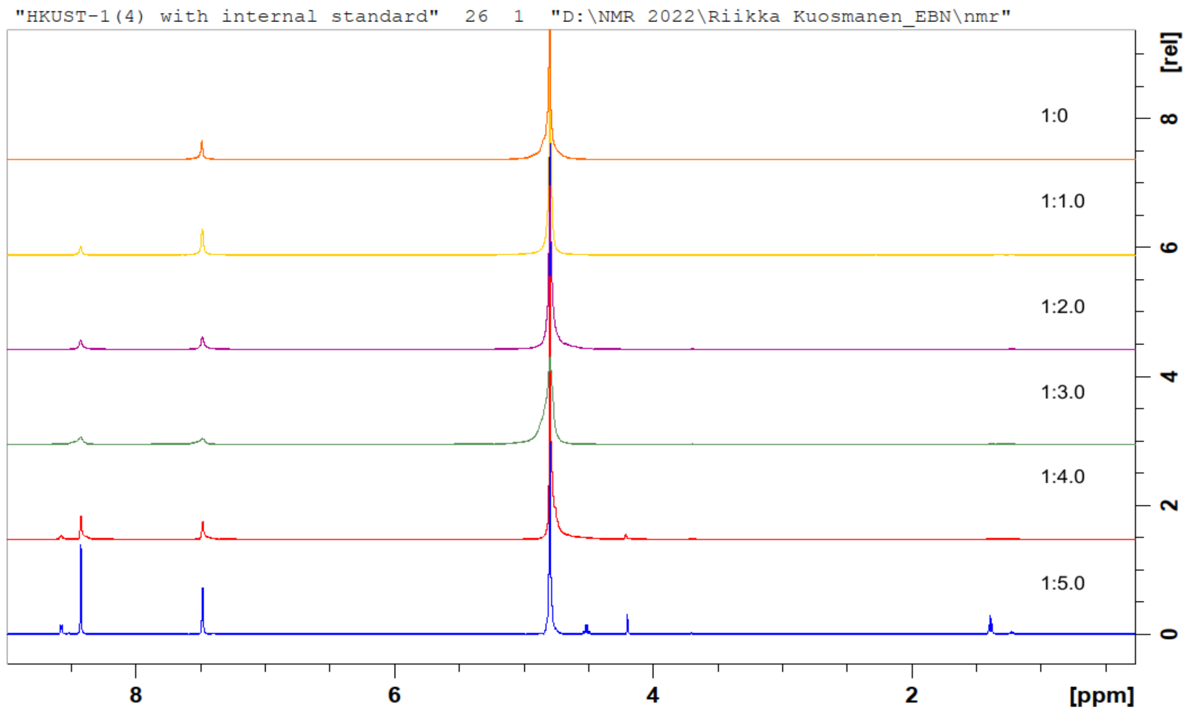

Figure S22. Whole titration of HKUST-1 modified with 4-picolylamine, with internal standard.

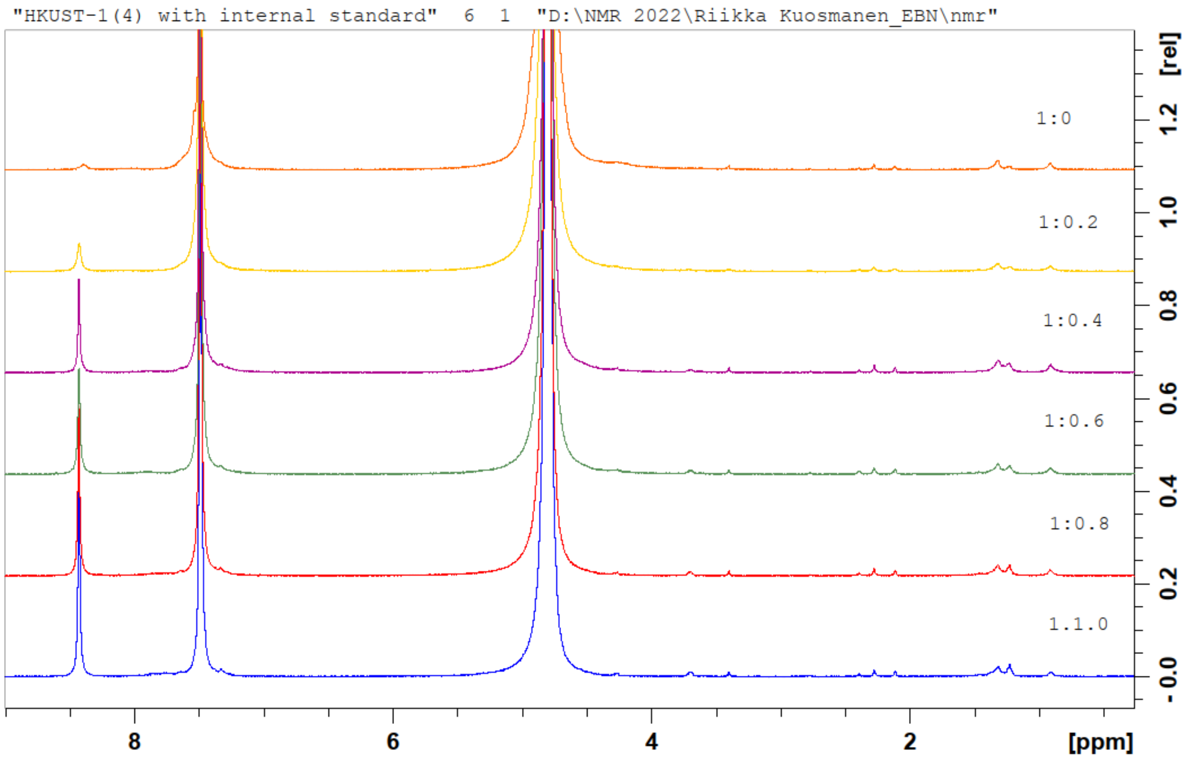

Figure S23. The beginning of titration of HKUST-1 modified with 4-picolylamine, with internal standard.

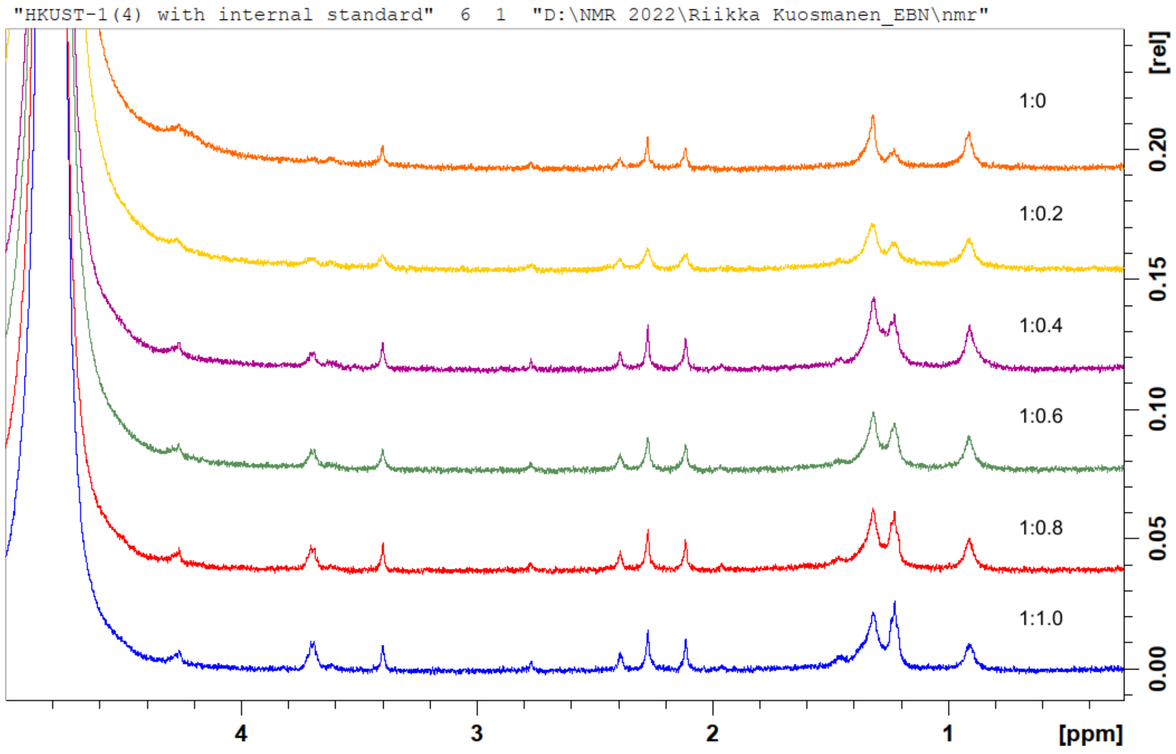

Figure S24. The beginning of titration of HKUST-1 modified with 4-picolylamine, with internal standard, from aliphatic region.

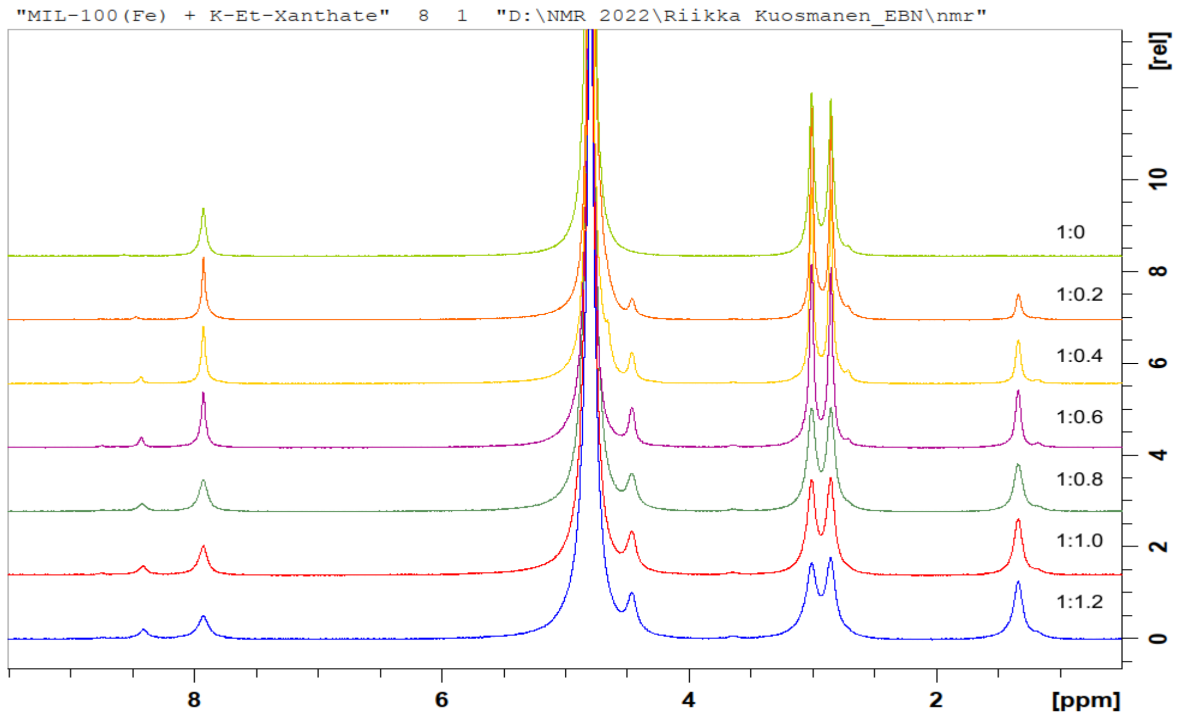

Figure S25. The whole titration of pristine MIL-100(Fe).

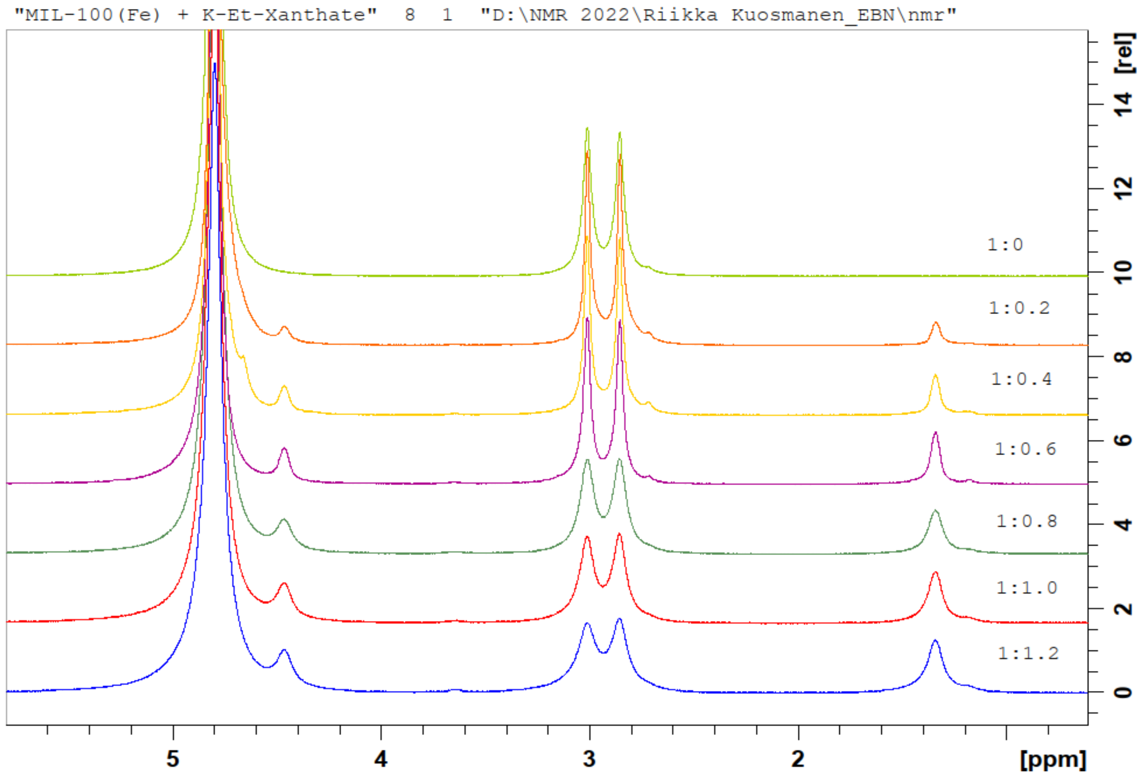

Figure S26. The titration of pristine MIL-100(Fe), aliphatic region.

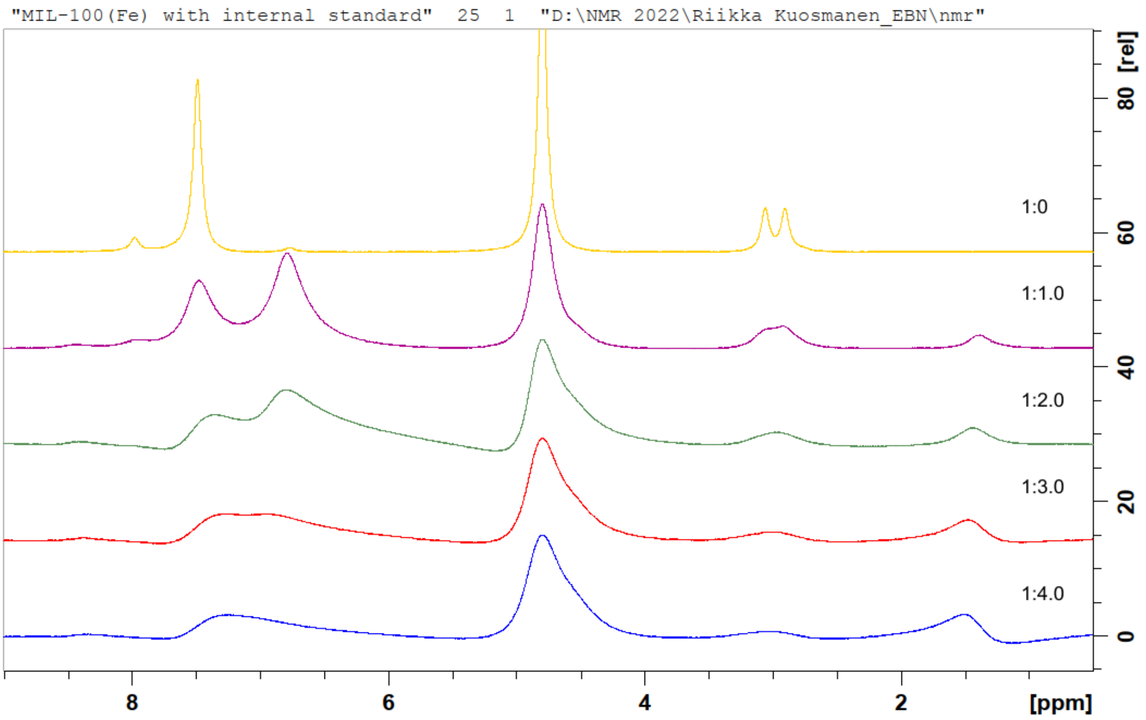

Figure S27. The whole titration of pristine MIL-100(Fe) with internal standard.

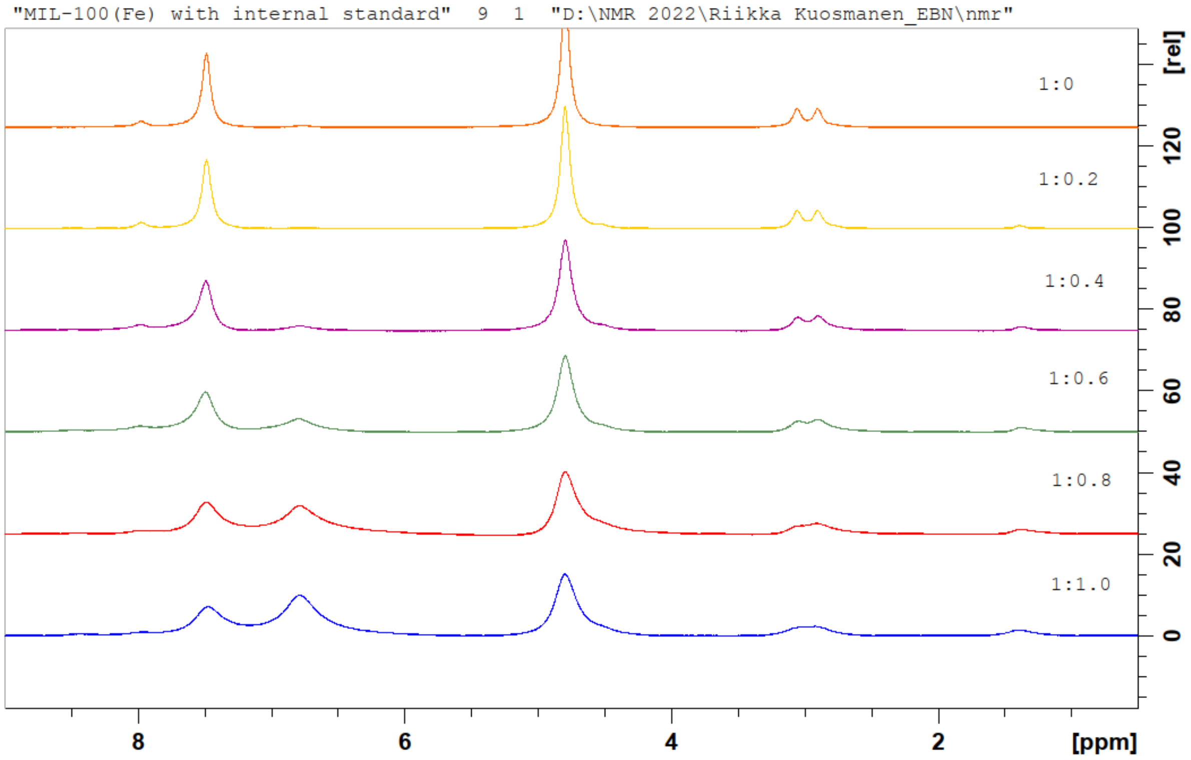

Figure S28. The beginning of titration of pristine MIL-100(Fe) with internal standard.

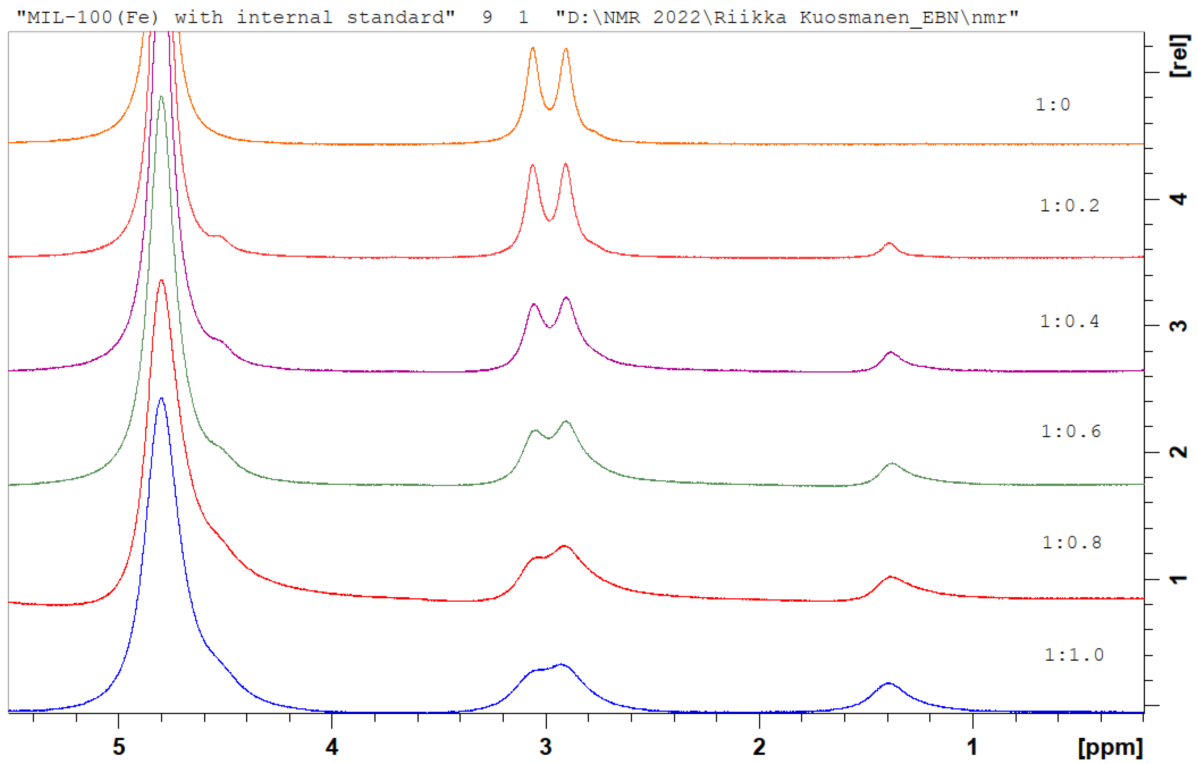

Figure S29. The beginning of titration of pristine MIL-100(Fe) with internal standard, aliphatic region.

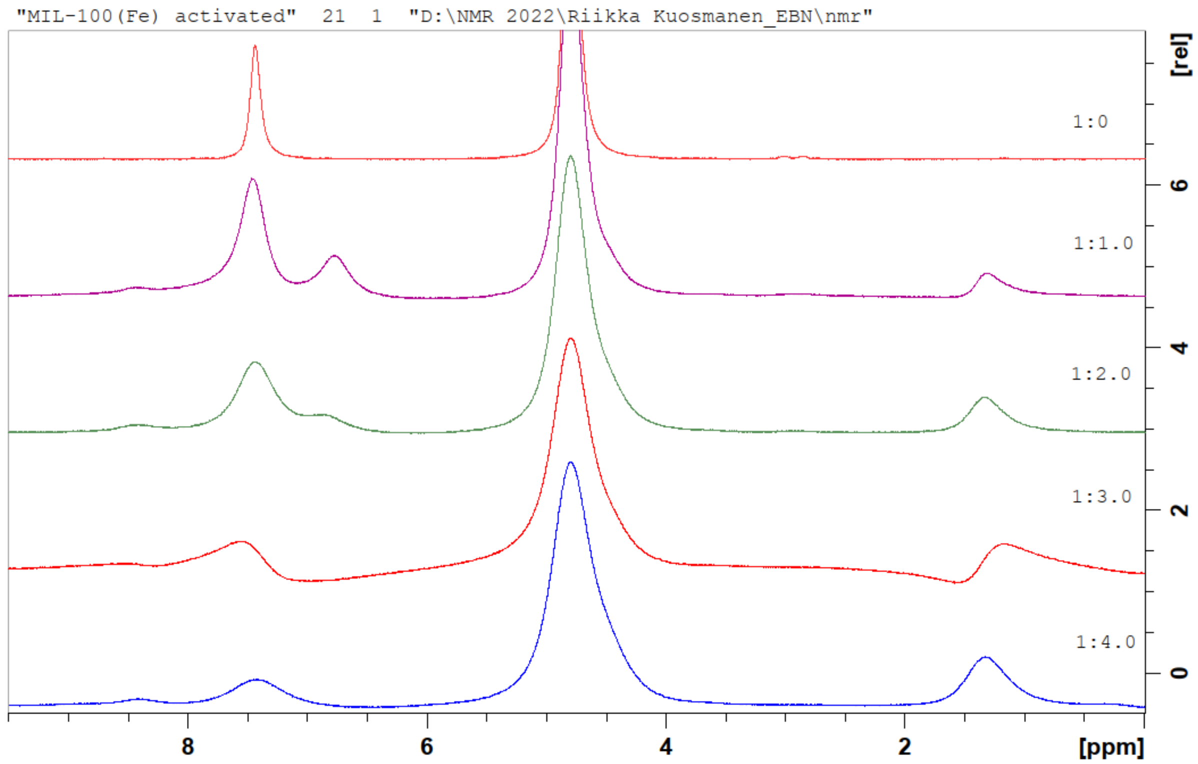

Figure S30. The whole titration of activated MIL-100(Fe) with internal standard.

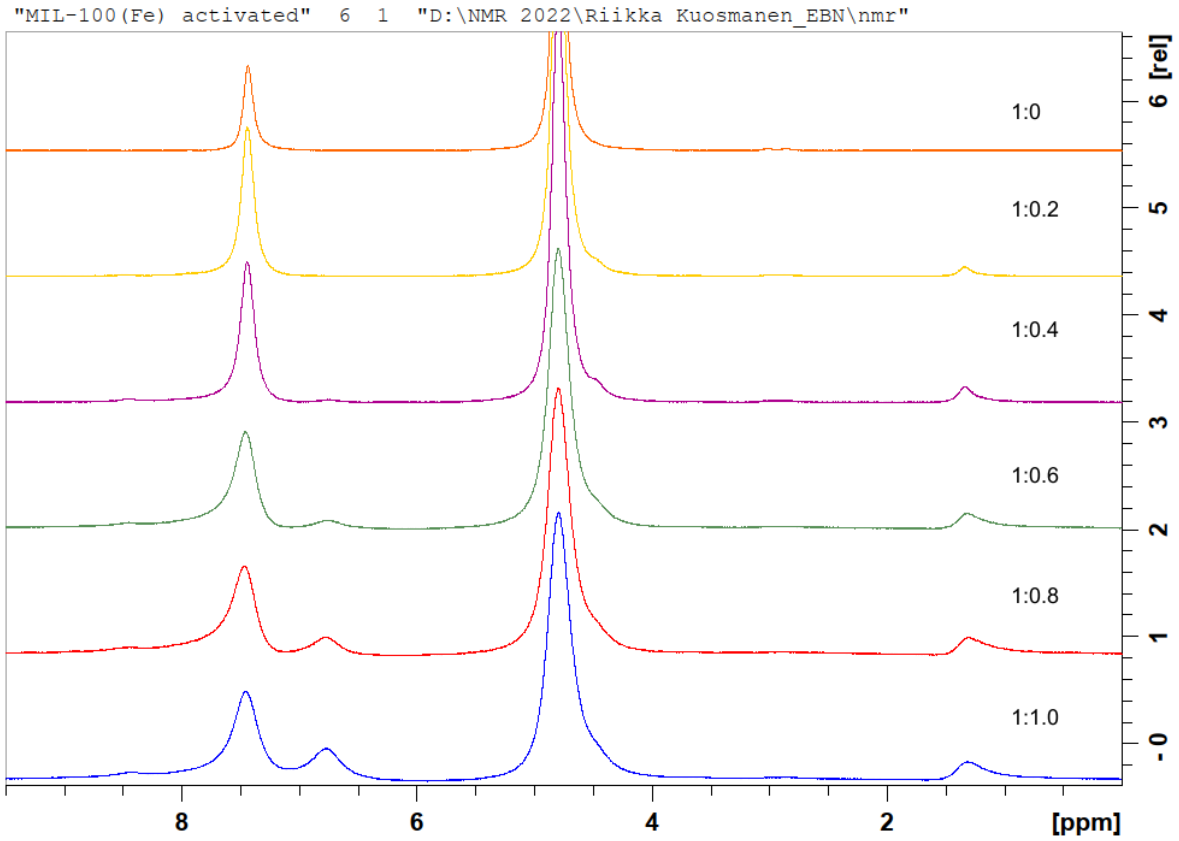

Figure S31. The beginning of titration of activated MIL-100(Fe) with internal standard.

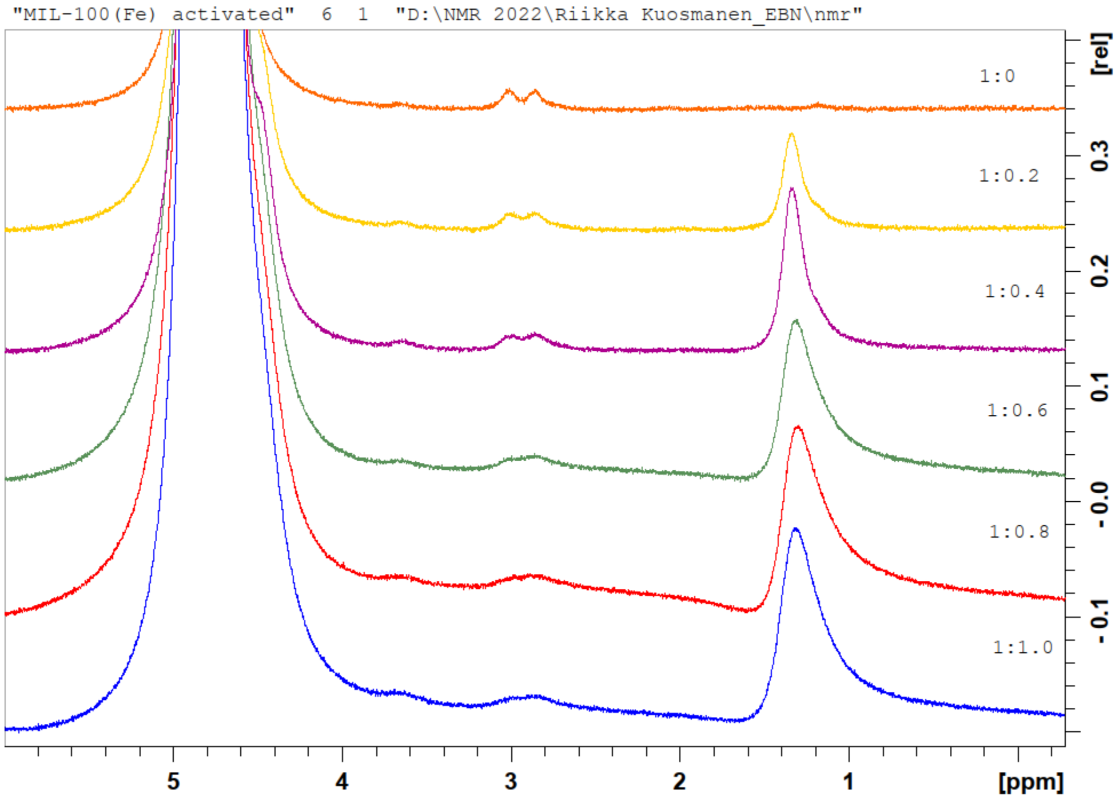

Figure S32. The beginning of titration of activated MIL-100(Fe) with internal standard, aliphatic region.

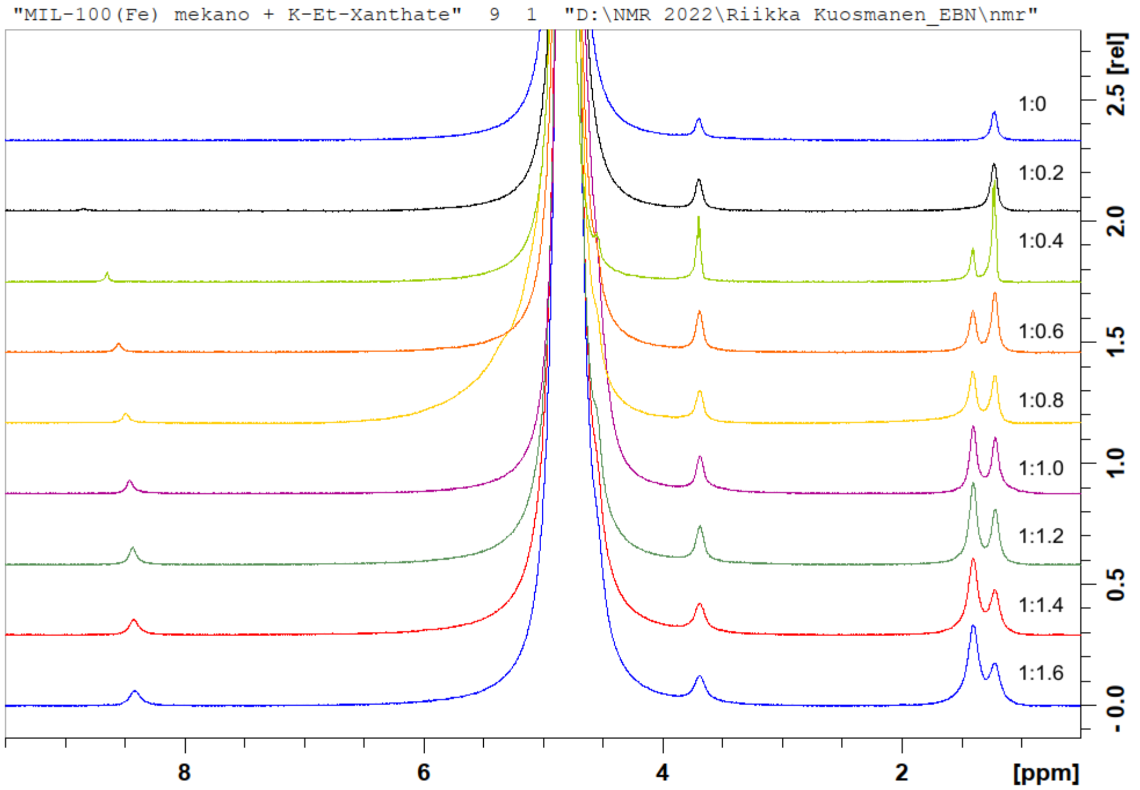

Figure S33. The whole titration of pristine mechanochemical MIL-100(Fe).

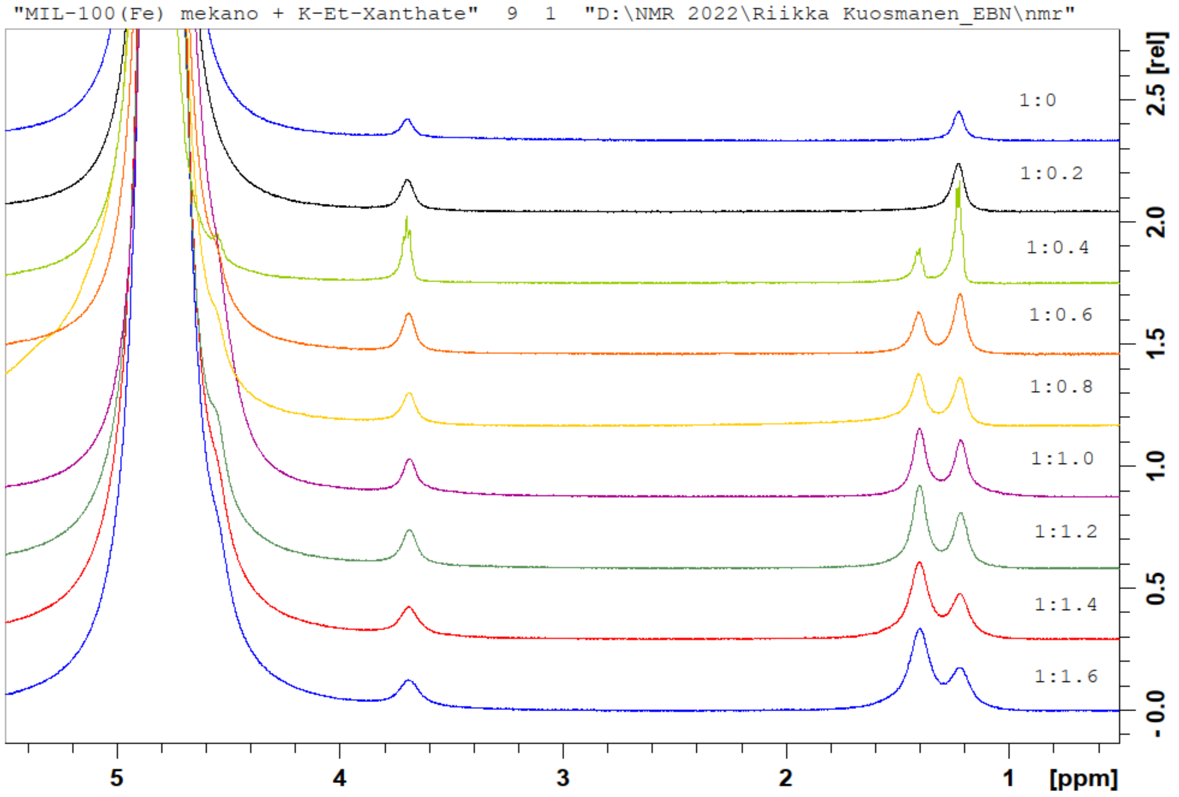

Figure S34. The whole titration of pristine mechanochemical MIL-100(Fe), aliphatic region.

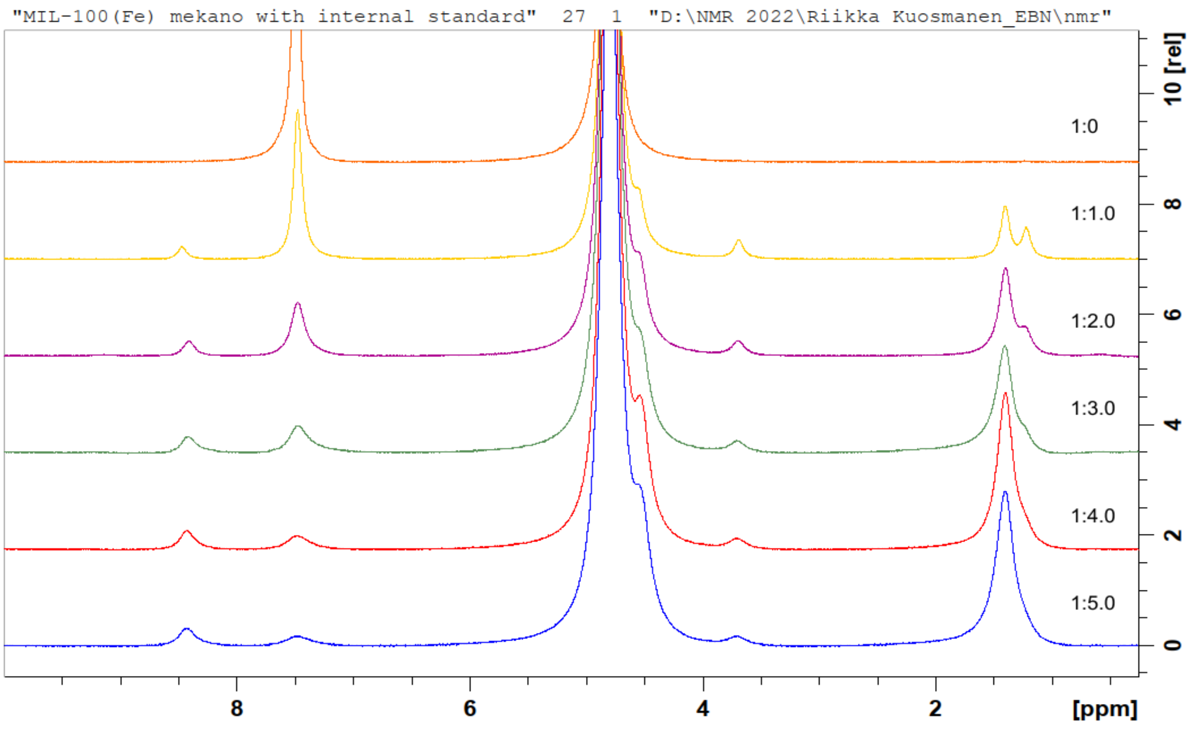

Figure S35. The whole titration of pristine mechanochemical MIL-100(Fe), with internal standard.

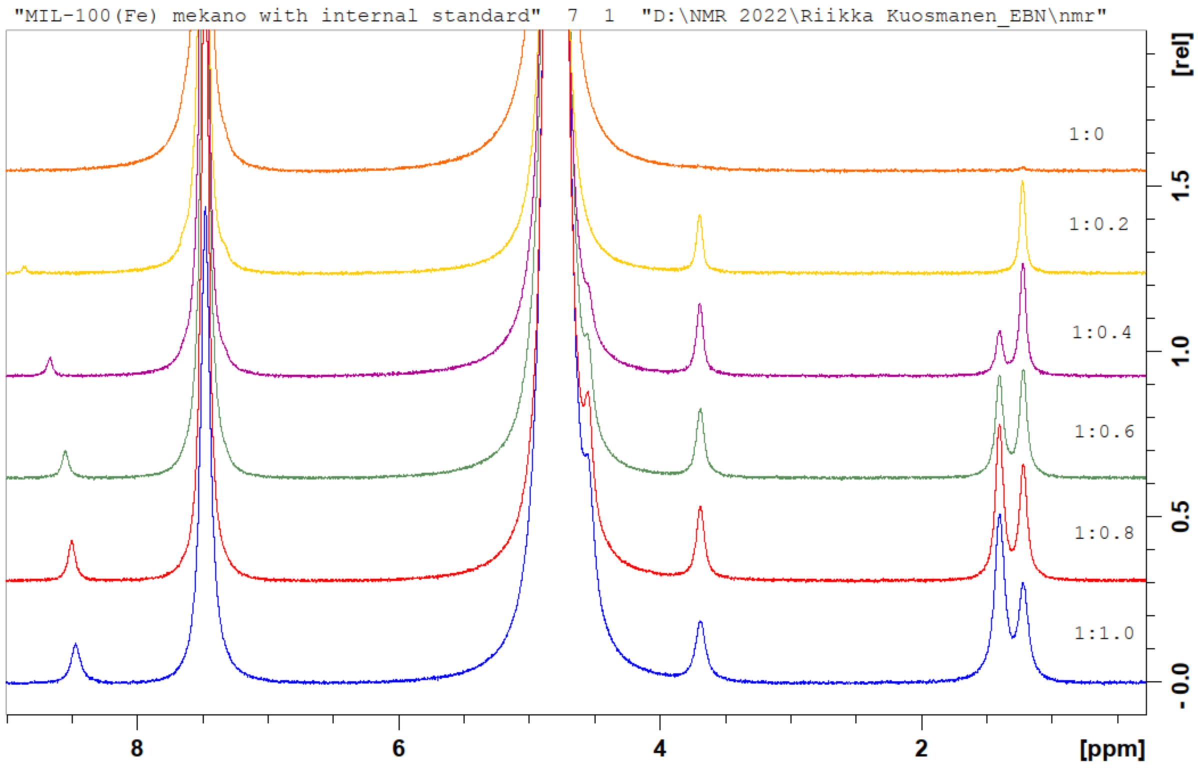

Figure S36. The beginning of titration of pristine mechanochemical MIL-100(Fe), with internal standard.

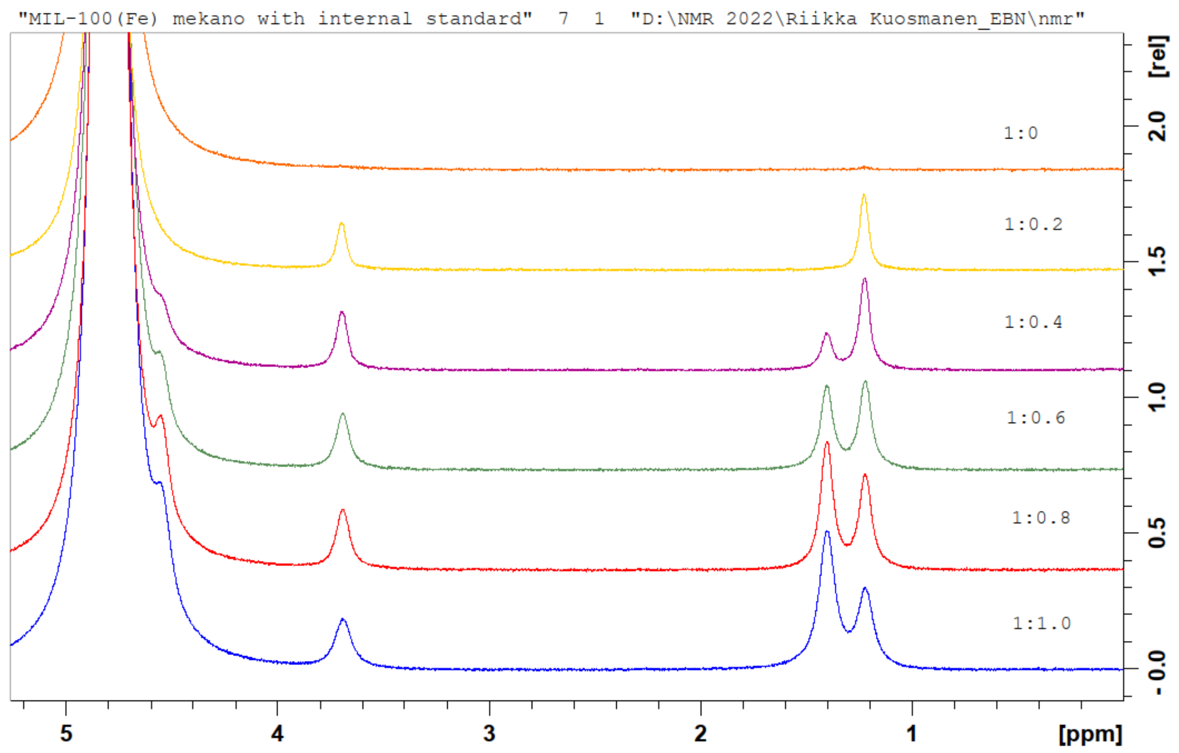

Figure S37. The beginning of titration of pristine mechanochemical MIL-100(Fe), with internal standard, aliphatic region.

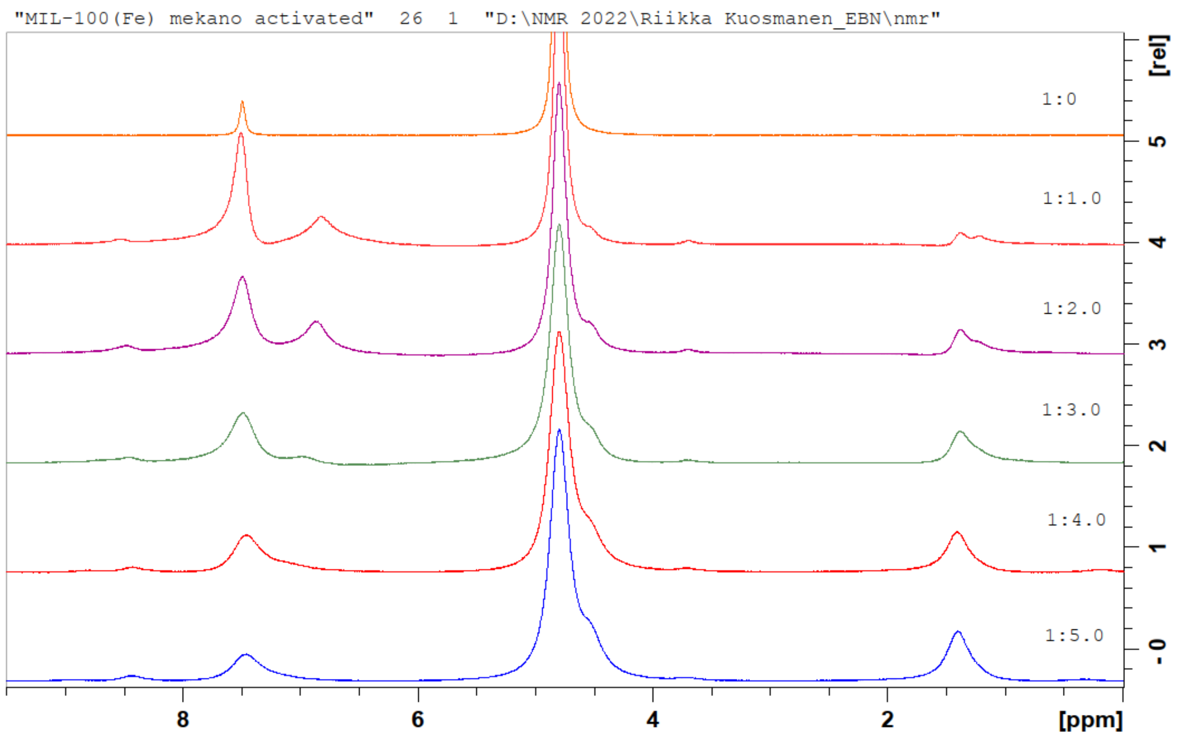

Figure S38. The whole titration of activated mechanochemical MIL-100(Fe), with internal standard.

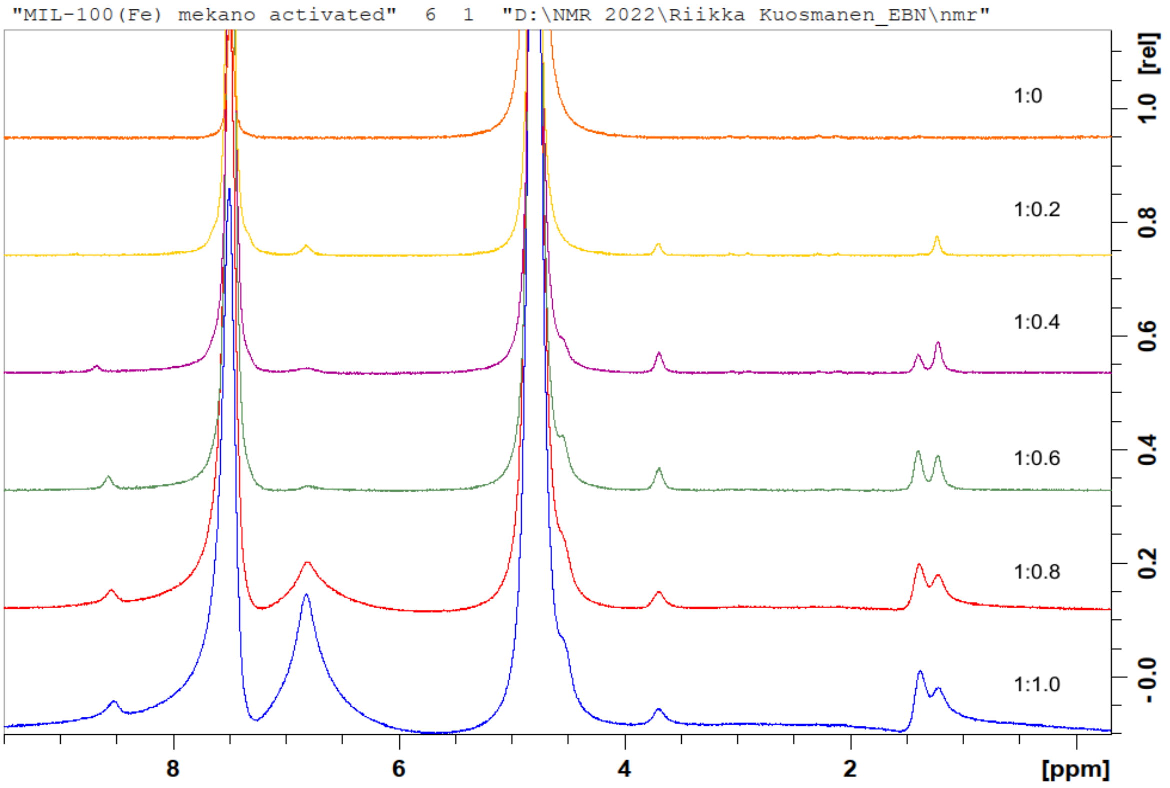

Figure S39. The beginning of titration of activated mechanochemical MIL-100(Fe), with internal standard.

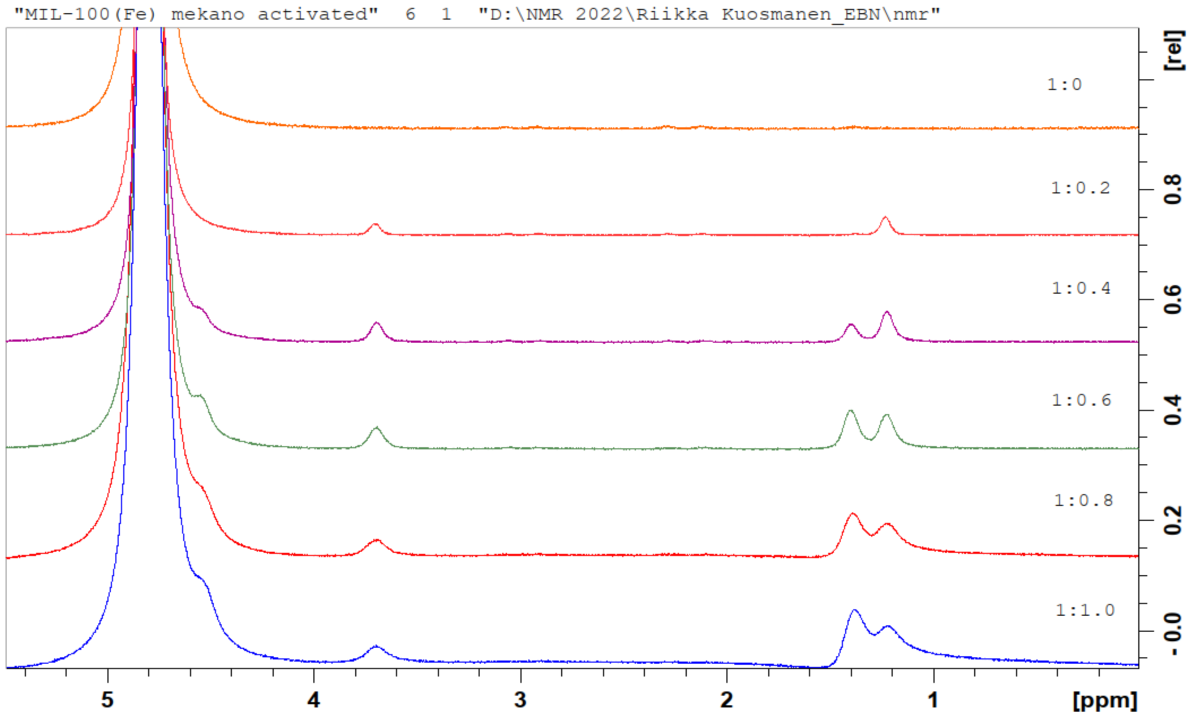

Figure S40. The beginning of titration of activated mechanochemical MIL-100(Fe), with internal standard, aliphatic region.

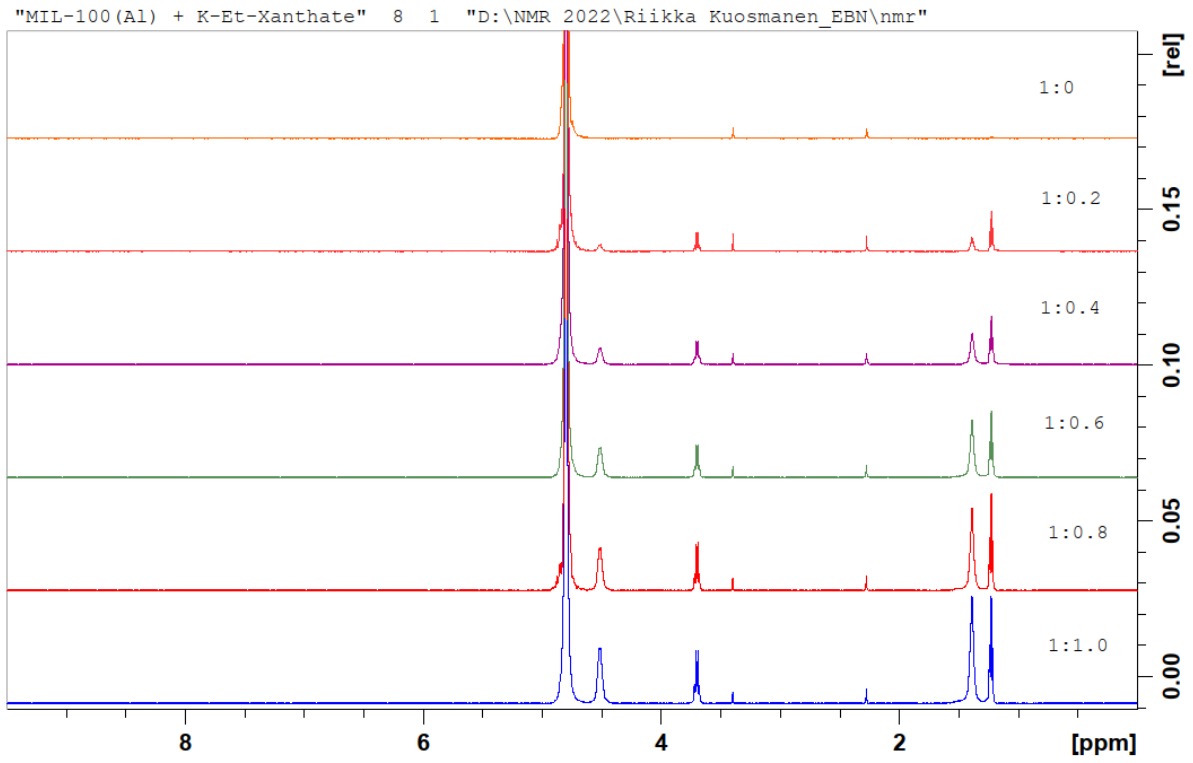

Figure S41. The whole titration of pristine MIL-100(Al).

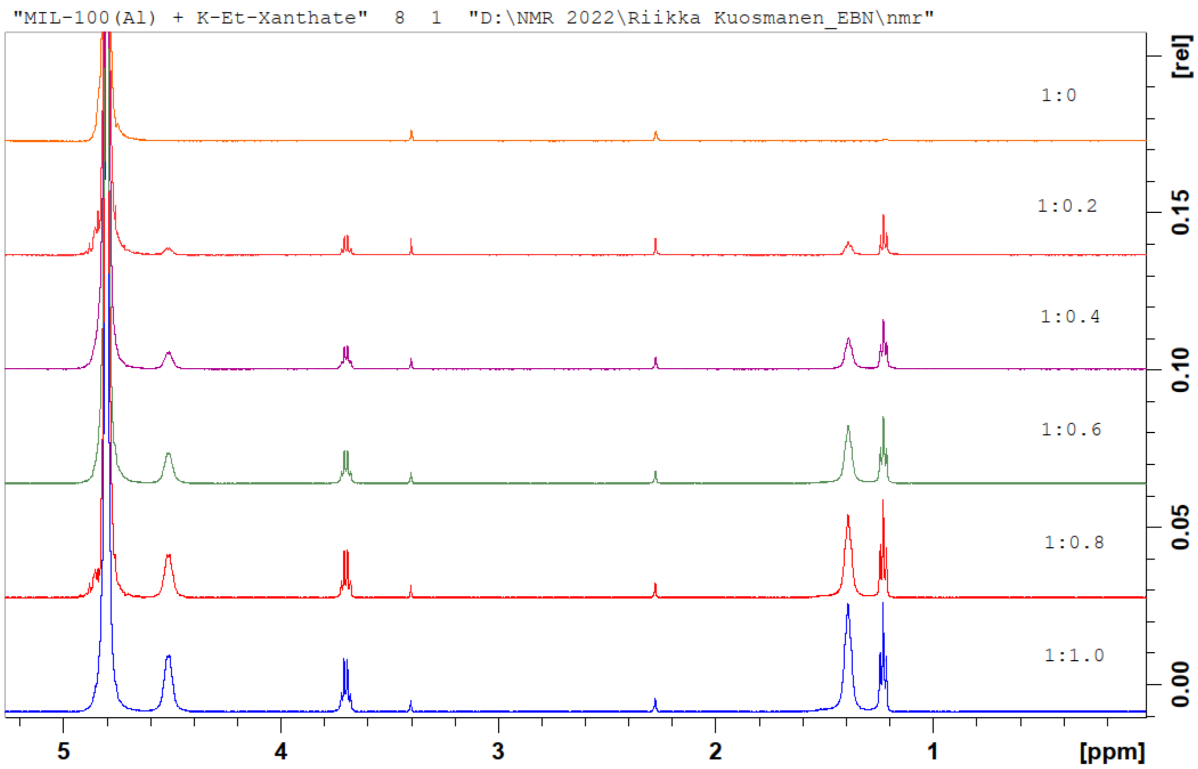

Figure S42. The beginning of titration of pristine MIL-100(Al), aliphatic region.

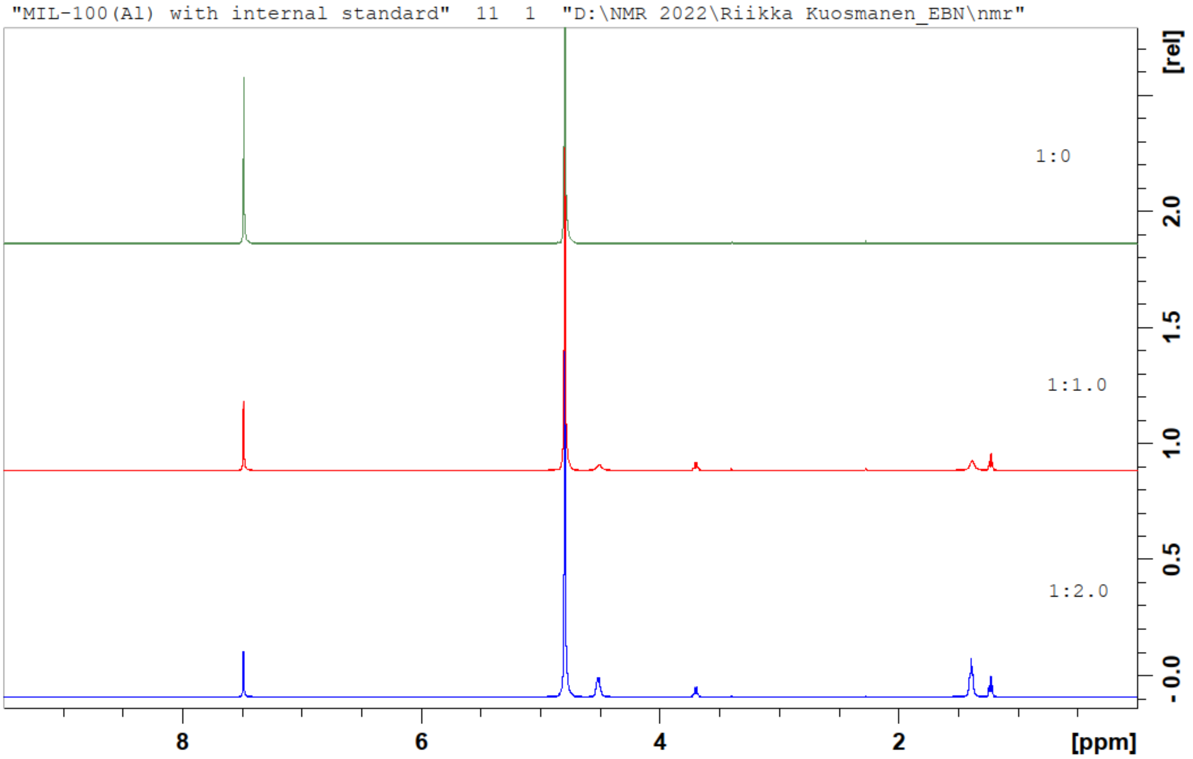

Figure S43. The whole titration of pristine MIL-100(Al) with internal standard.

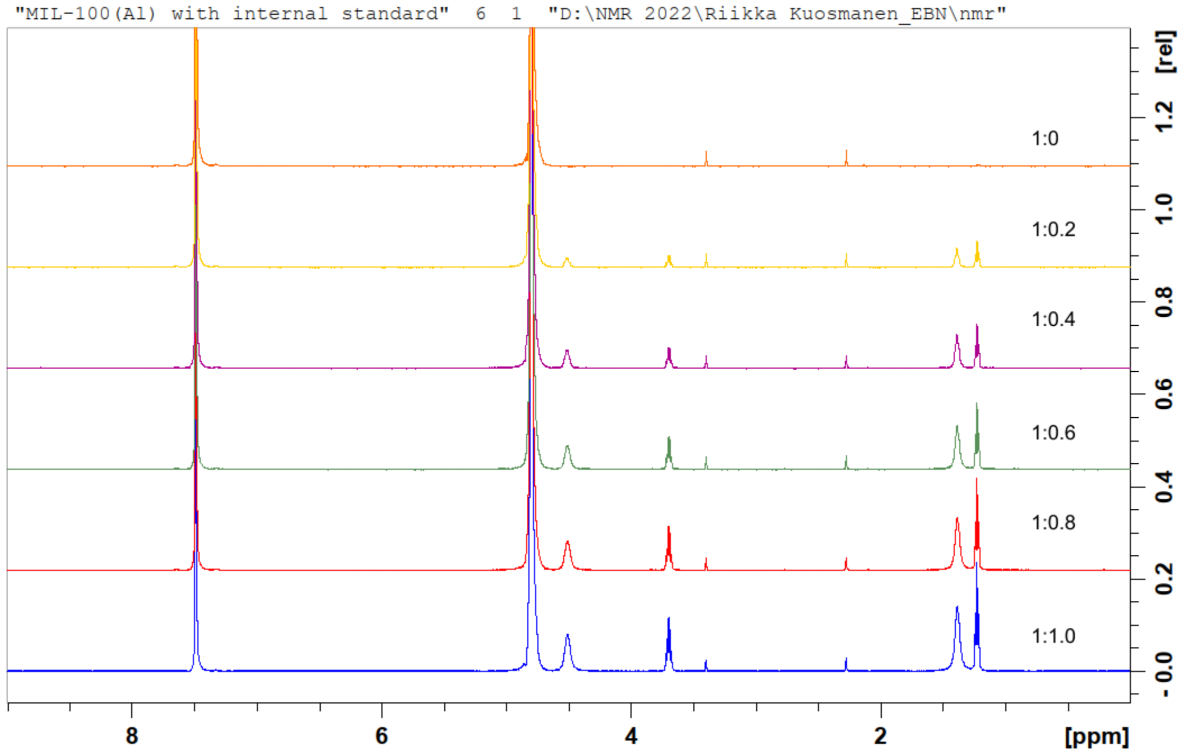

Figure S44. The beginning of titration of pristine MIL-100(Al) with internal standard.

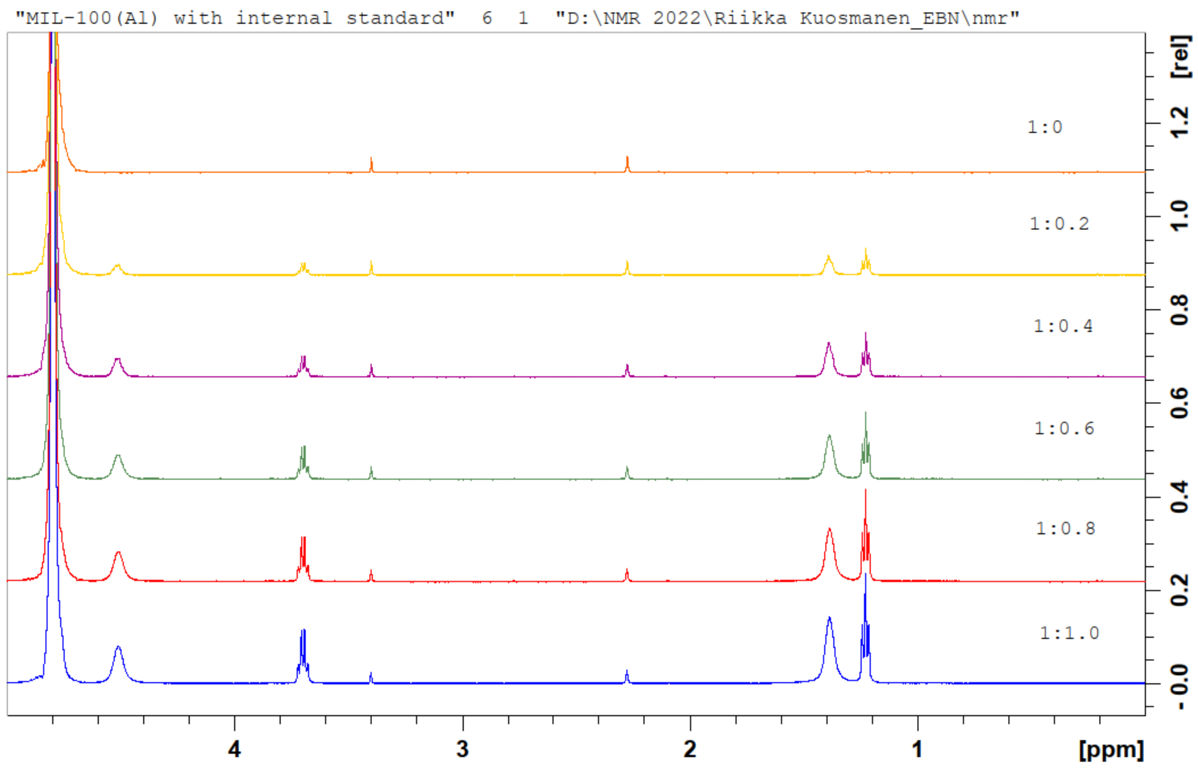

Figure S45. The beginning of titration of pristine MIL-100(Al) with internal standard, aliphatic region.

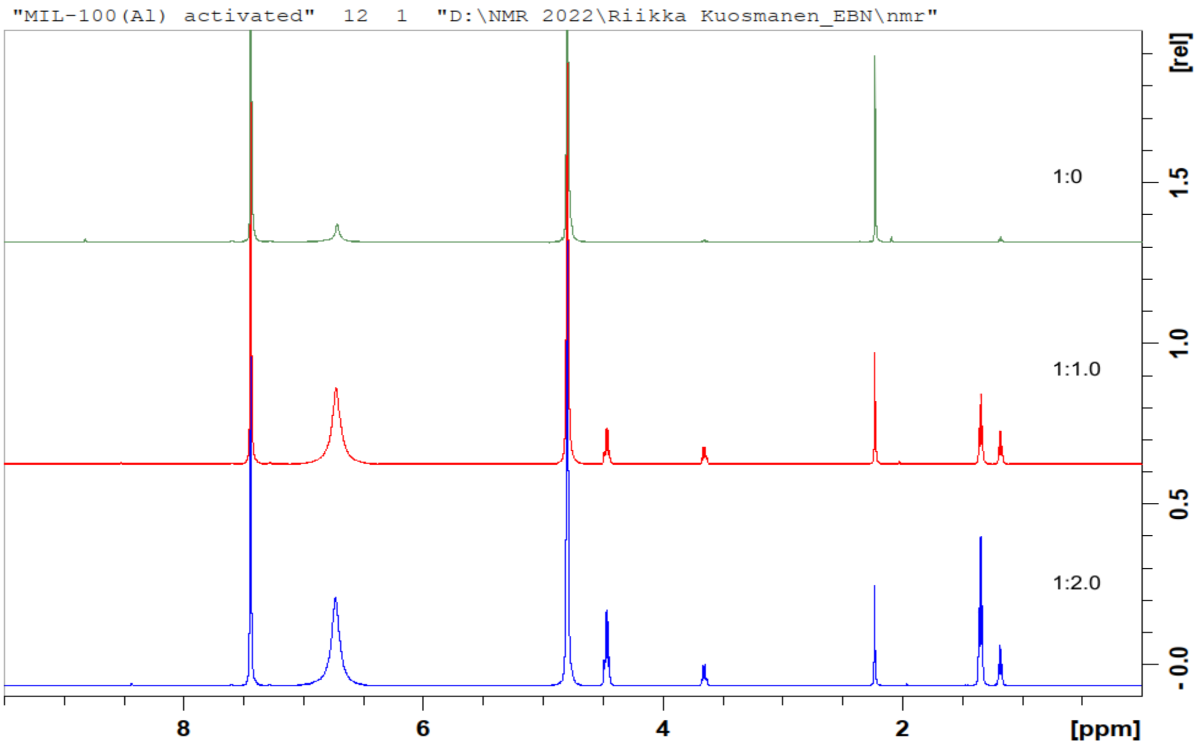

Figure S46. The whole titration of activated 1:1 MIL-100(Al):MIL-96(Al) with internal standard.

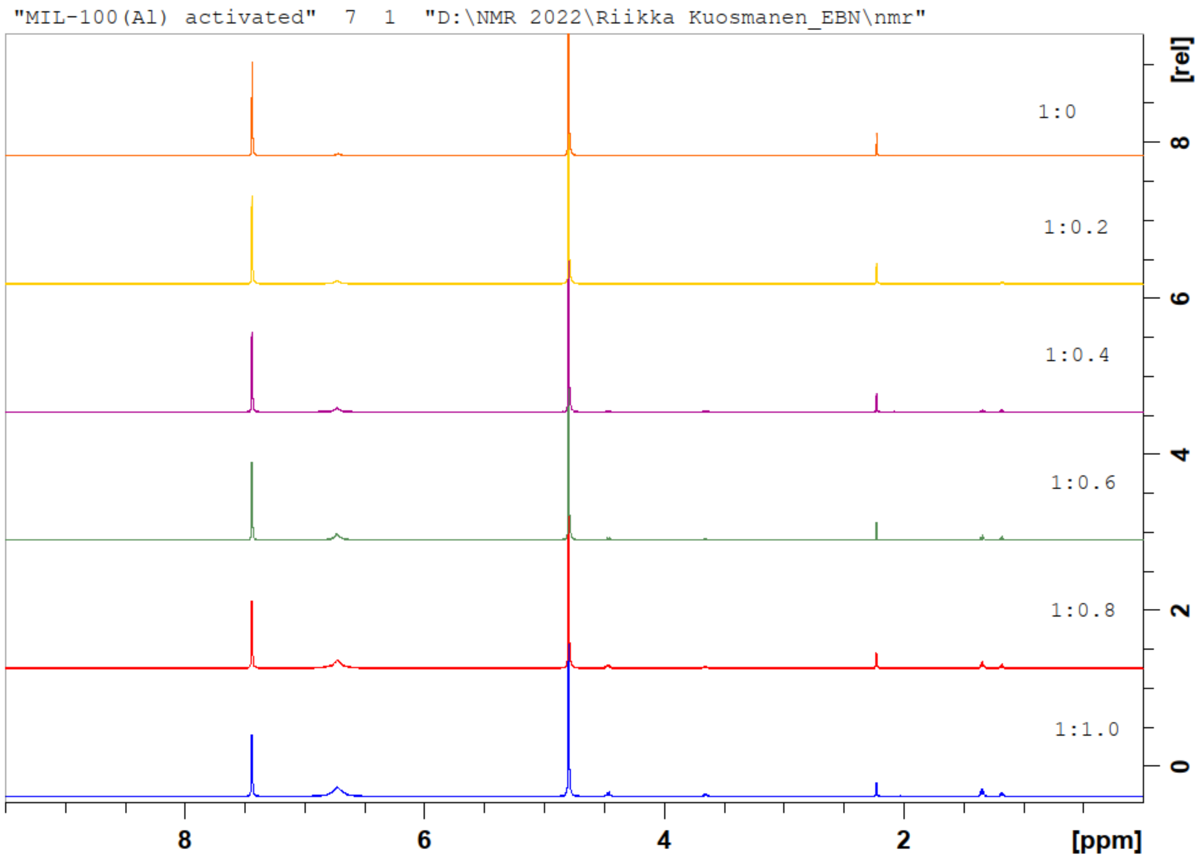

Figure S47. The beginning of titration of activated 1:1 MIL-100(Al):MIL-96(Al) with internal standard.

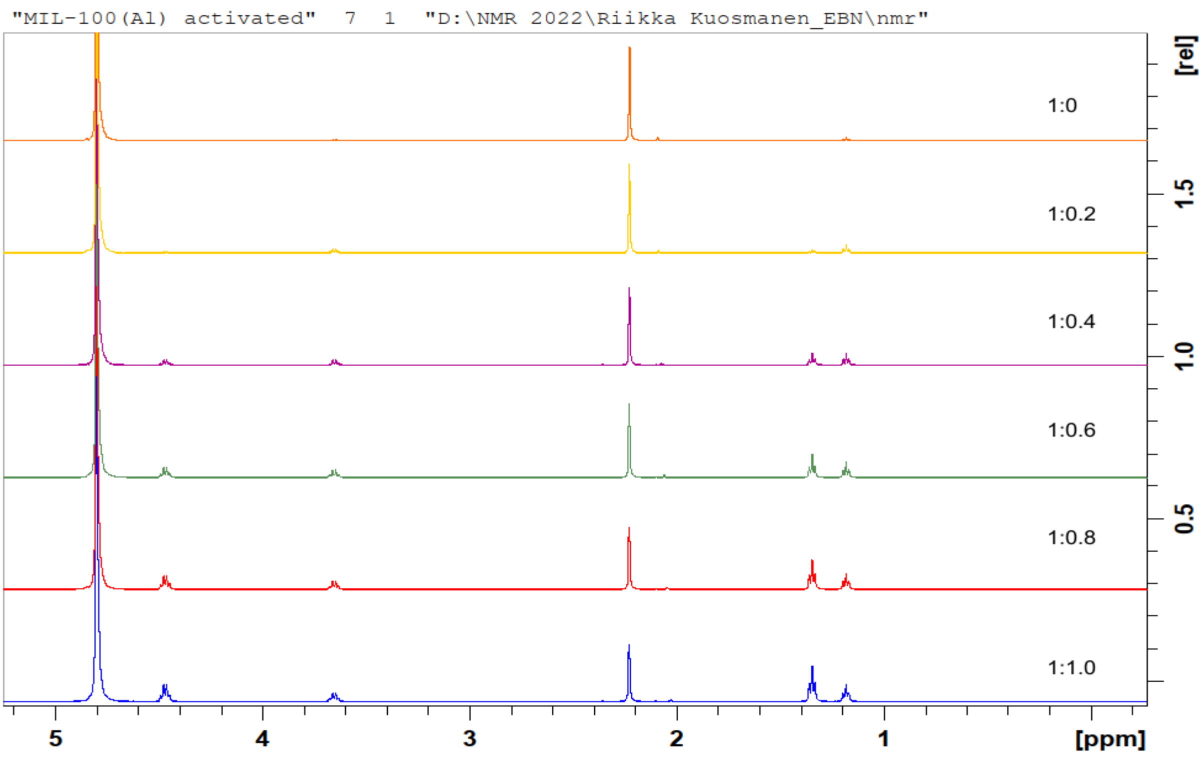

Figure S48. The beginning of titration of activated 1:1 MIL-100(Al):MIL-96(Al) with internal standard, aliphatic region.

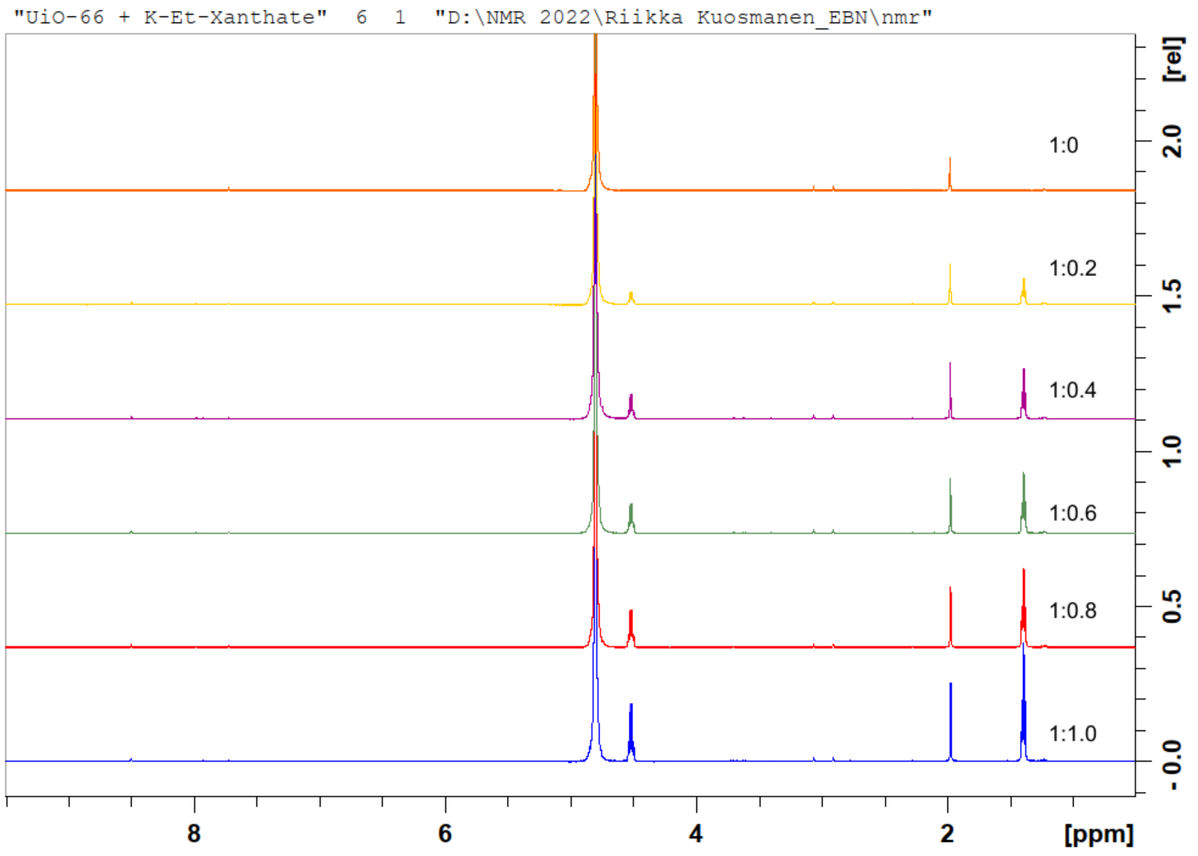

Figure S49. The whole titration of pristine UiO-66.

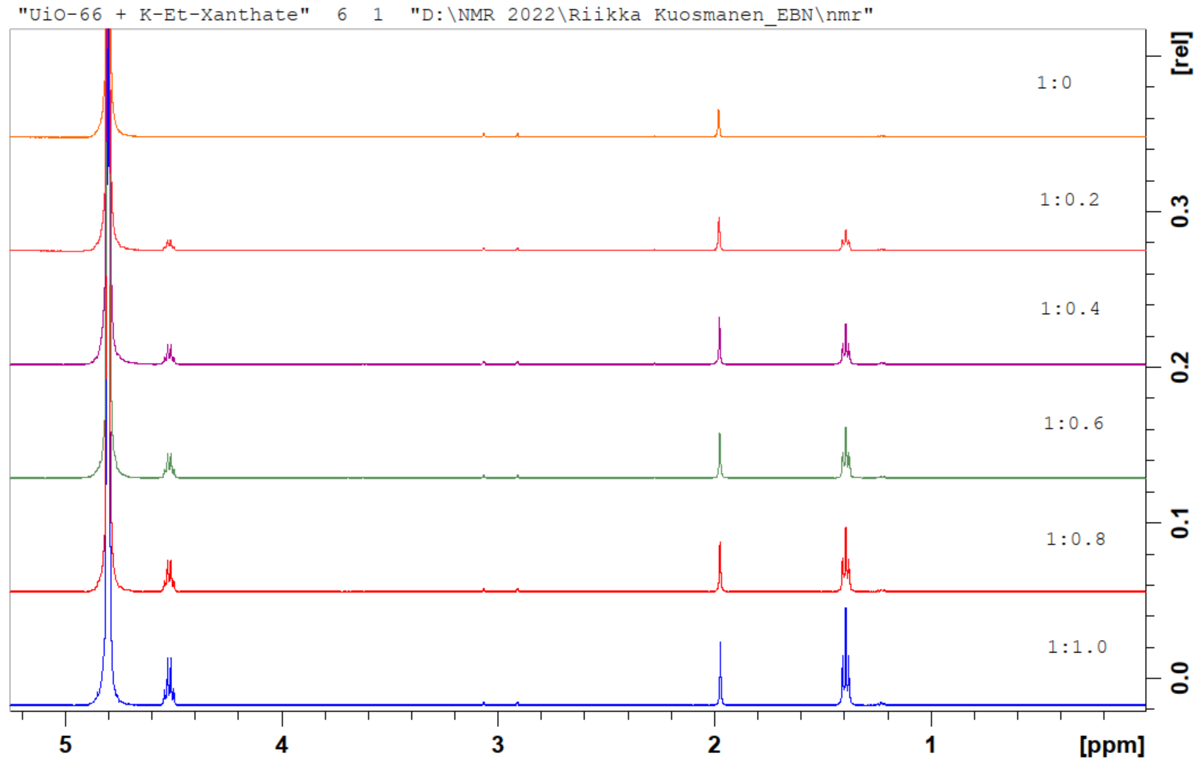

Figure S50. The whole titration of pristine UiO-66, aliphatic region.

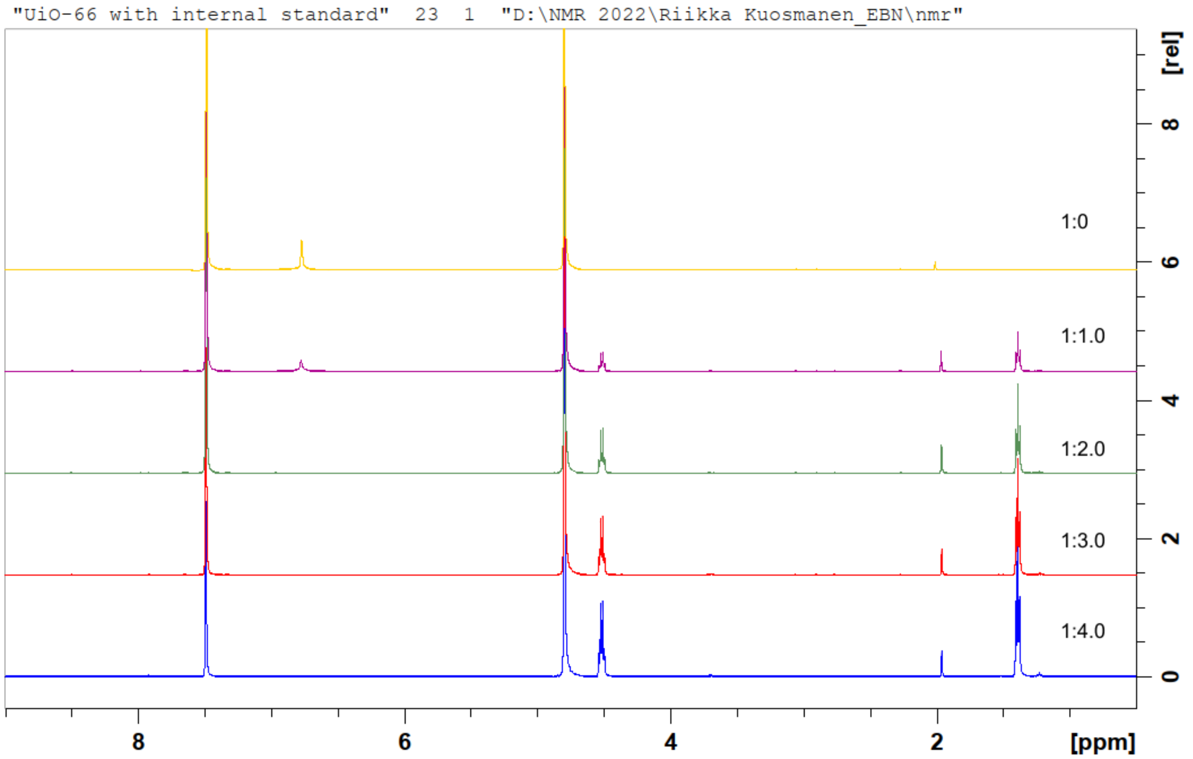

Figure S51. The whole titration of pristine UiO-66 with internal standard.

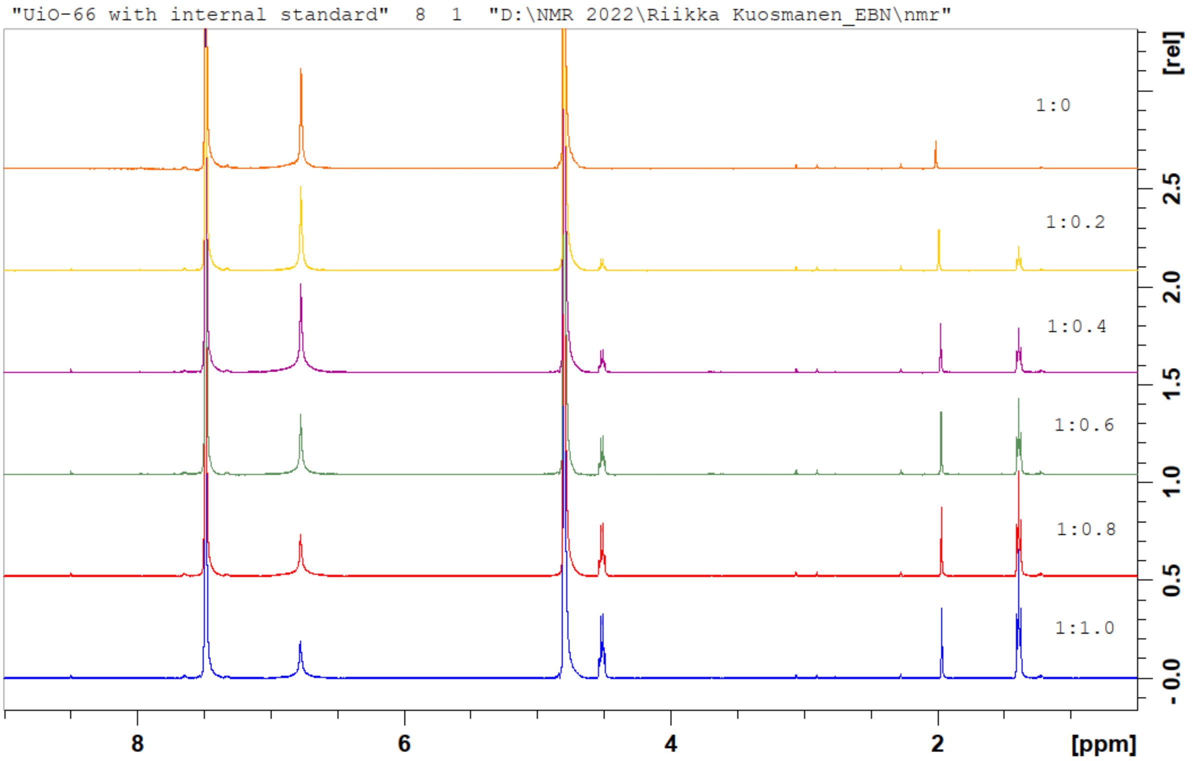

Figure S52. The beginning of titration of pristine UiO-66 with internal standard.

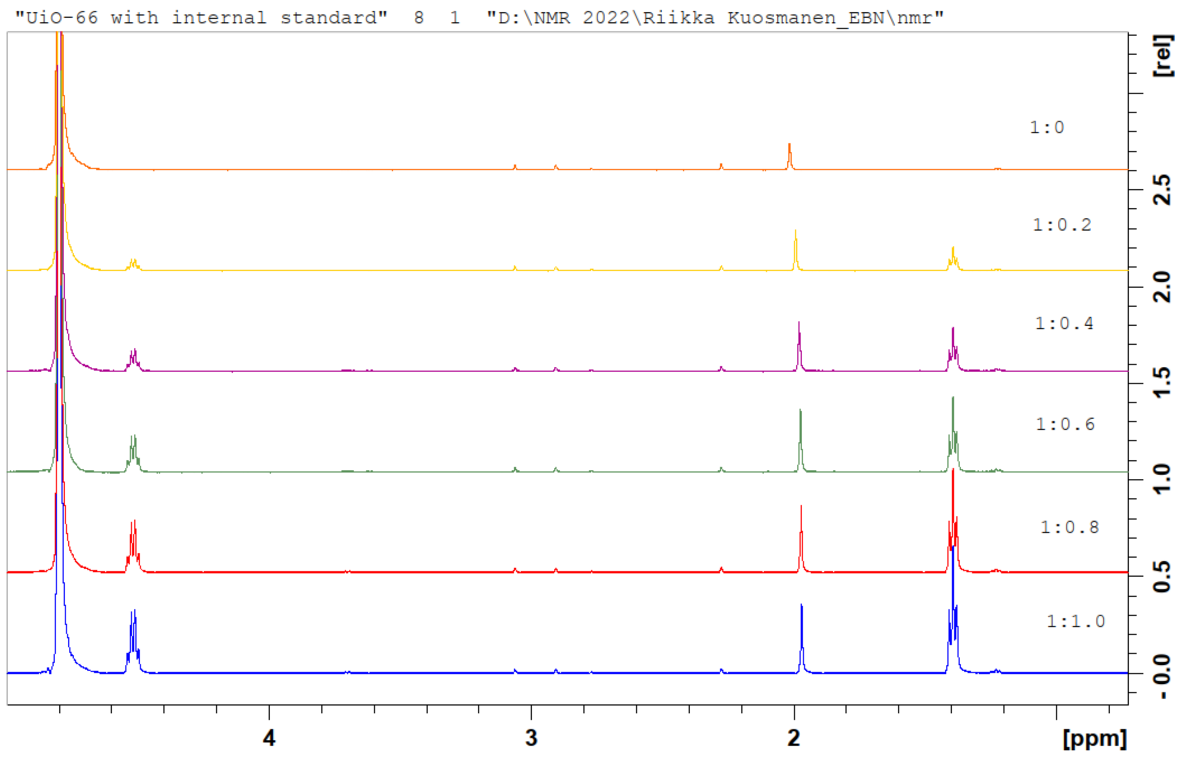

Figure S53. The beginning of titration of pristine UiO-66 with internal standard, aliphatic region.

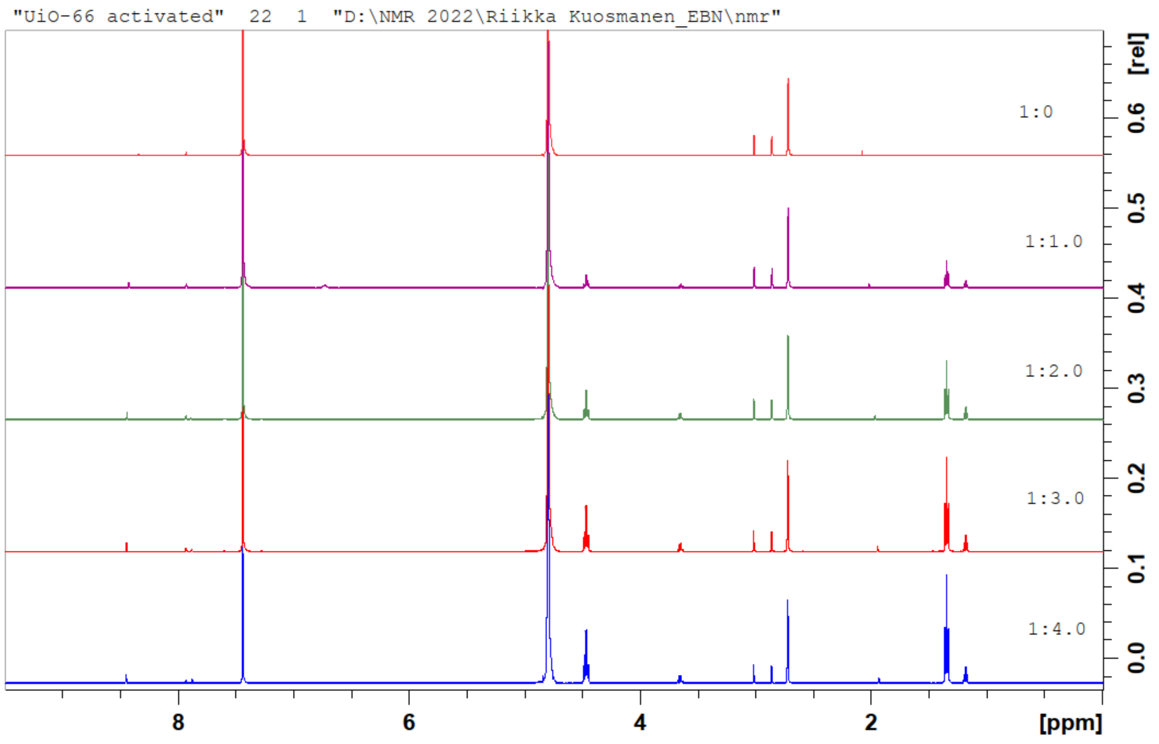

Figure S54. The whole titration of activated UiO-66 with internal standard.

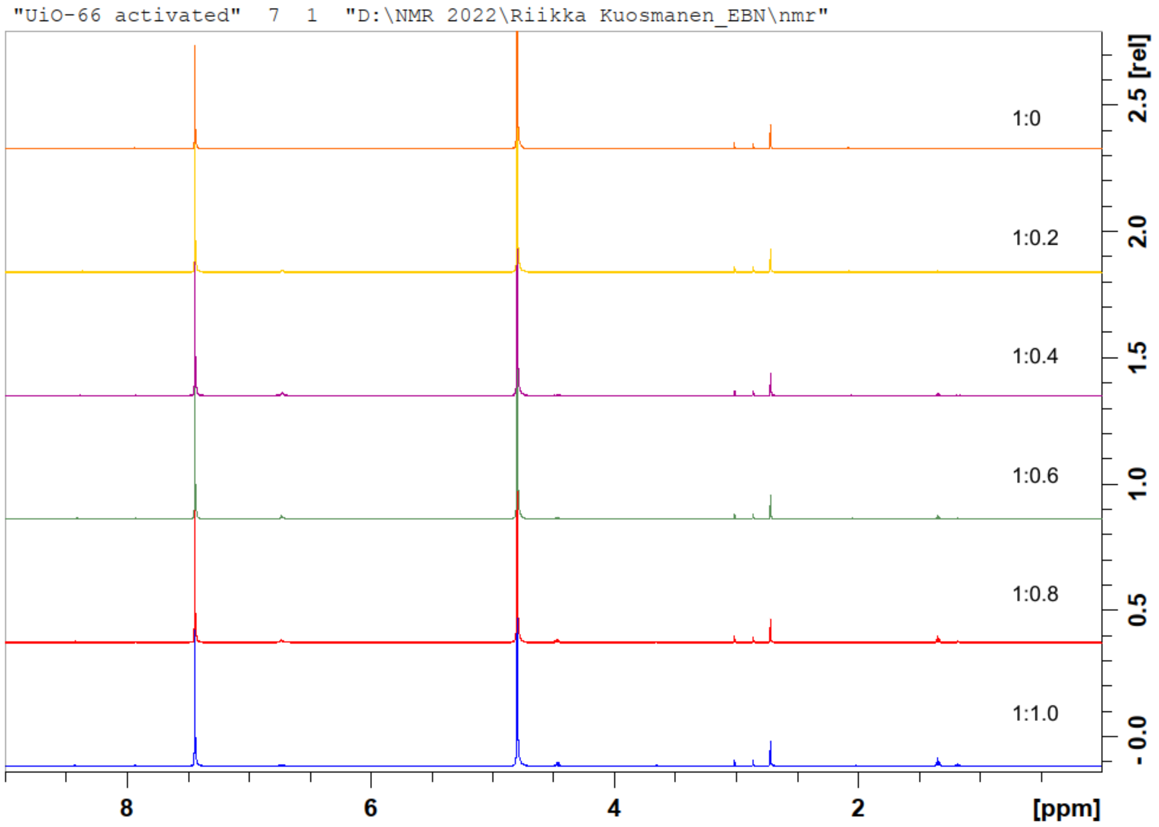

Figure S55. The beginning of titration of activated UiO-66 with internal standard.

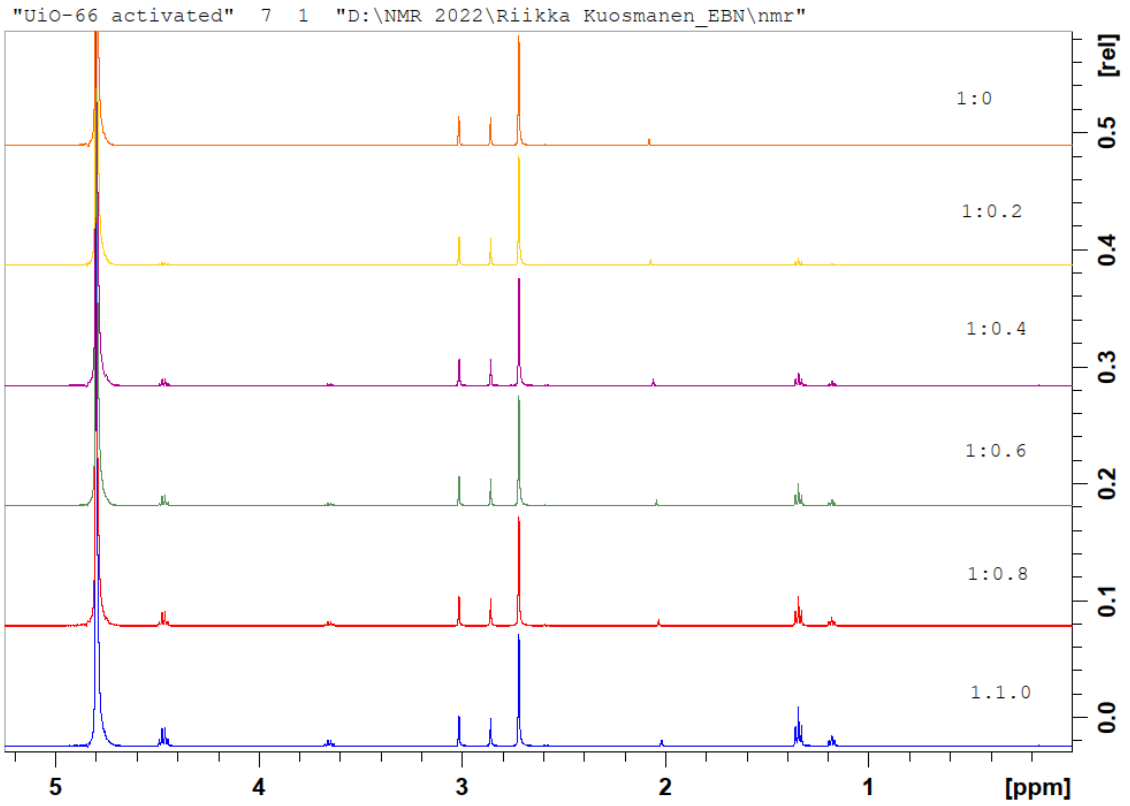

Figure S56. The beginning of titration of activated UiO-66 with internal standard, aliphatic region.

Table S6: The integrals and ratios of integrals of bound and free KEX during the titrations. The integral values are from the points, where the coordination of KEX was at its highest. Abbreviations: pristine (P), activated (A), mechanochemically synthetized (M), without/with internal standard (-SI and +SI), coordinated (C), free (F), and ratio (R). R=C/F.

MOF	MOF:KEX	CH_3 C	CH_3 F	R of CH_3s	CH_2 C	CH_2 F	R of CH_2s
HKUST-1 P (-IS)	-	-	-	-	-	-	-
HKUST-1 P (+IS)	1:1.2	0.0621	0.0095	6.54:1	0.0359	0.0051	7.04:1
HKUST-1 A (+IS)	-	-	-	-	-	-	-
HKUST-1 with 3-PA (-IS)	-	-	-	-	-	-	-
HKUST-1 with 3-PA (+IS)	1:0.6	0.0215	0.0027	7.96:1	0.0134	0.0038	3.53:1
HKUST-1 with 4-PA (-IS)	-	-	-	-	-	-	-
HKUST-1 with 4-PA (+IS)	1:3.6	0.1098	0.0537	2.04:1	0.0487	0.0260	1.87:1
MIL-100(Fe) P (-IS)	1:0.6	0.0916	1.1934	0.08:1	0.0464	0.9611	0.05:1
MIL-100(Fe) P (+IS)	-	-	-	-	-	-	-
MIL-100(Fe) A (+IS)	-	-	-	-	-	-	-
MIL-100(Fe) M P (-IS)	-	-	-	-	-	-	-
MIL-100(Fe) M P (+IS)	1:0.4	0.1009	0.0401	2.52:1	0.0647	-	-
MIL-100(Fe) M A (+IS)	1:0.2	0.0150	0.0010	15:1	0.0096	-	-
MIL-100(Al) P (-IS)	1:0.2	3.8648	0.1456	26.5:1	6.3964	4.8204	1.33:1
MIL-100(Al) P (+IS)	1:0.2	0.0375	0.0532	0.70:1	0.0630	0.0855	0.74:1
MIL-100(Al) A (+IS)	1:0.2	0.0280	0.0100	2.80:1	0.0416	0.0161	2.58:1
UiO-66 P (-IS)	1:1.2	-	62.3580	-	-	40.6525	-
UiO-66 P (+IS)	1:1.2	-	0.231	-	-	0.150	-
UiO-66 A (+IS)	1:1.2	0.0214	0.0832	1:3.89	0.0328	0.1297	1:3.95

The integral values are not provided, if there were no signals from free or coordinated KEX available. Thus, there was no ratio to be calculated.

Solid state NMR

Samples for solid state measurements were prepared from pristine HKUST-1 and UiO-66. In addition, KEX and copper xanthate were measured. Samples of MOF:KEX from 1:1 to 1:4 in the case of HKUST-1 and MOF:KEX 1:1 in the case of UiO-66 were prepared in ultrapure water. To a small beaker 200 mg of MOF was weighed and appropriate amount of KEX in 10 mL of ultrapure water was added. The

resulting suspension was stirred at room temperature for 40 min (1:1), 80 min (1:2), 120 min (1:3), or 160 min (1:4) after which the samples were filtered with a glass sinther and transferred to CPMAS rotor immediately. Measurement time was 9 days for HKUST-1 samples and 2 days for UiO-66 samples.

^{13}C CPMAS NMR spectra of the MOFs, ethyl xanthate, copper ethyl xanthate and ligands were recorded at room temperature with Bruker Avance 400 MHz spectrometer equipped with a SB 4 mm CPMAS probe, using 4 mm ZrO_2 rotors. The solid samples were spun at a rate of 10 kHz. The CP contact time was 2 ms and relaxation delay 5 or 6 s depending on the measurement. Adamantane was used in the calibration of the instrument.

FTIR

FTIR measurements were conducted for activated MOFs and 1:1 ratio (MOF:KEX) samples. Samples were prepared similarly to the solid state NMR measurements. 10 mg of MOF in question was weighed and appropriate amount of KEX in ultrapure water was added. After 40 min the sample was either filtered with glass sinther or centrifugated, after which the sample was measured. Bruker Alpha Platinum ATR was used to measure the spectra and data was handled with Opus 7.0 program.

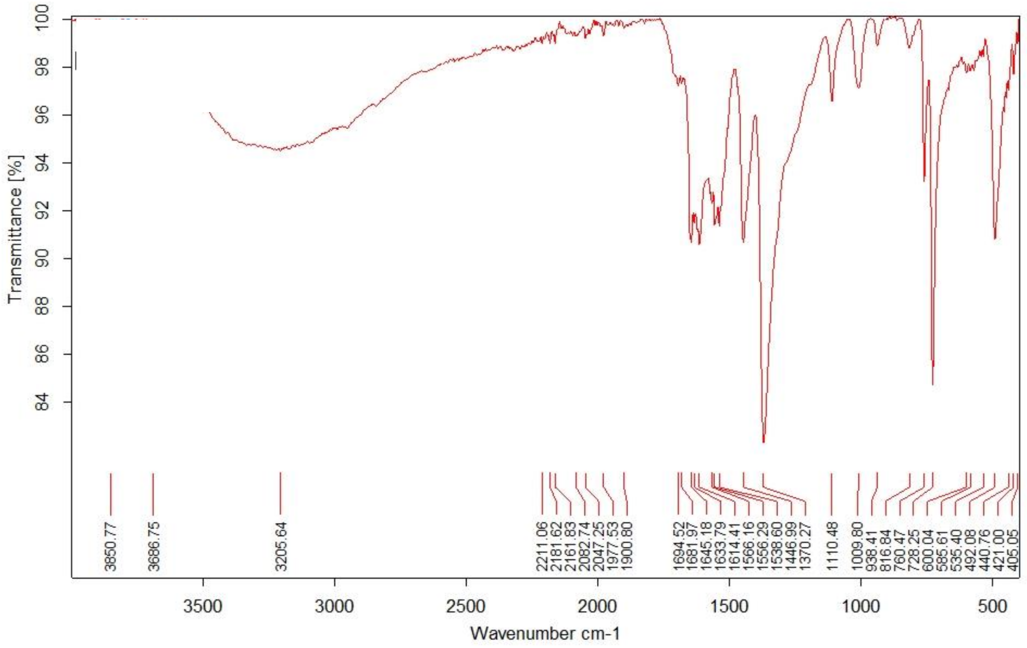

Figure S57. FTIR spectrum of activated HKUST-1.

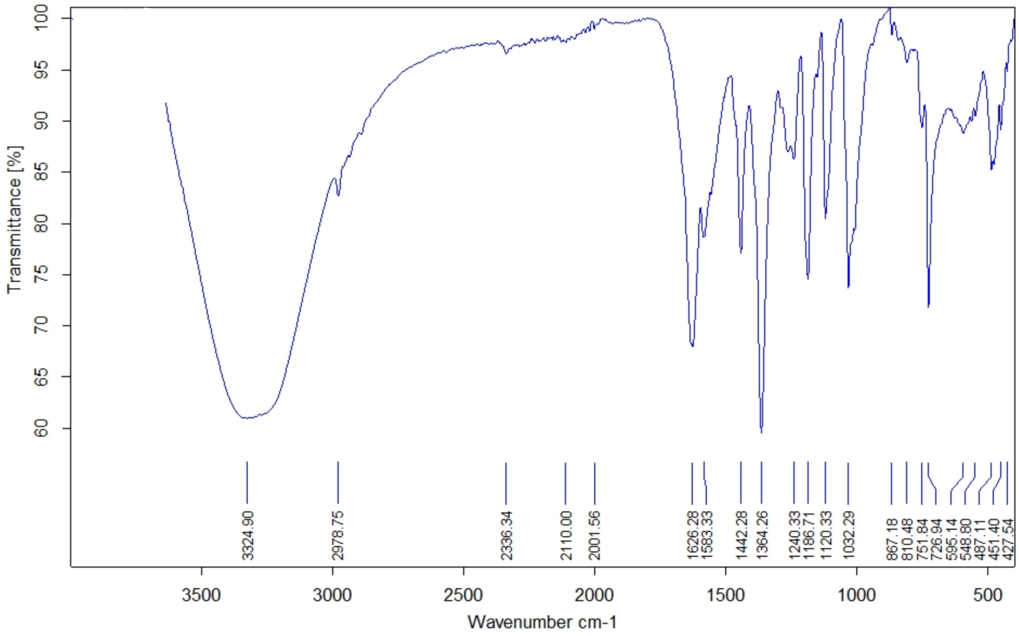

Figure S58. FTIR spectrum of activated HKUST-1 with KEX (1:1).

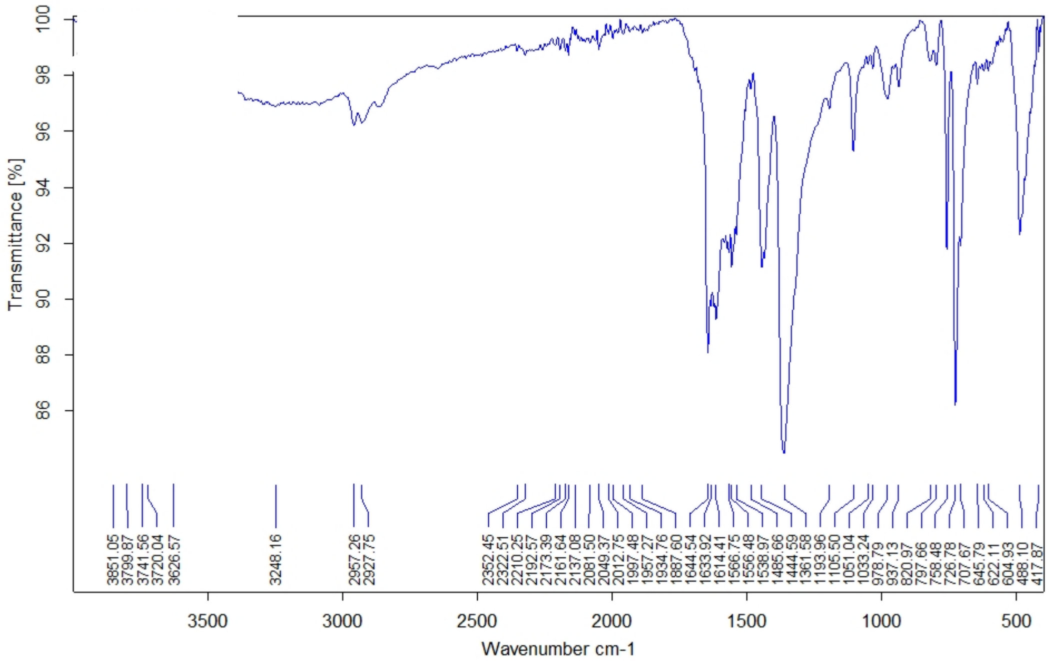

Figure S59. FTIR spectrum of activated HKUST-1 post-synthetically modified with 3-PA.

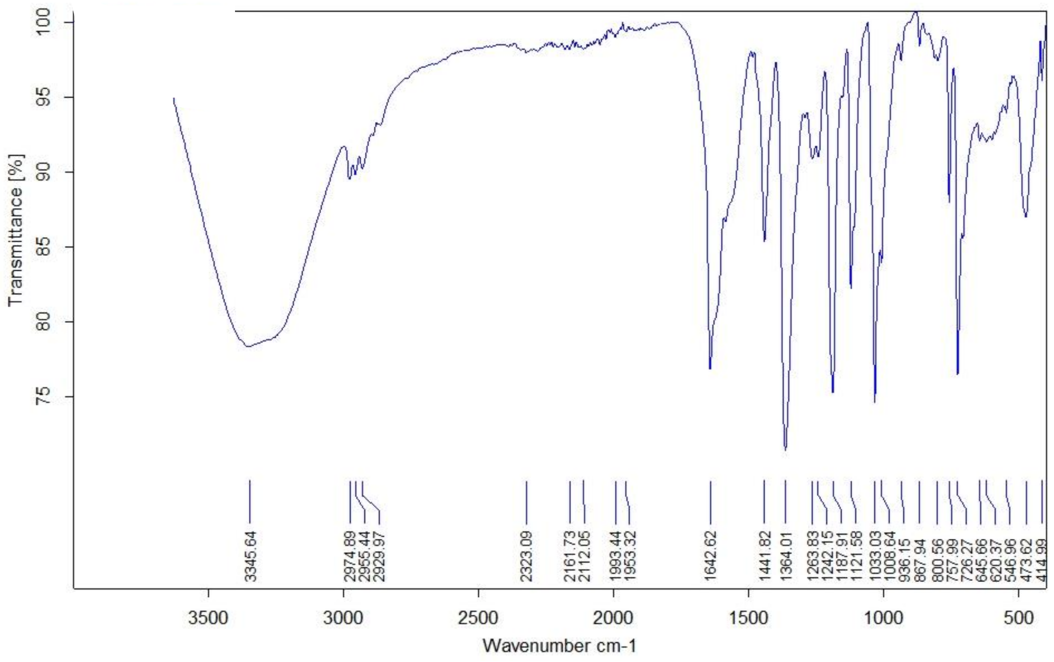

Figure S60. FTIR spectrum of activated HKUST-1 post-synthetically modified with 3-PA with KEX (1:1).

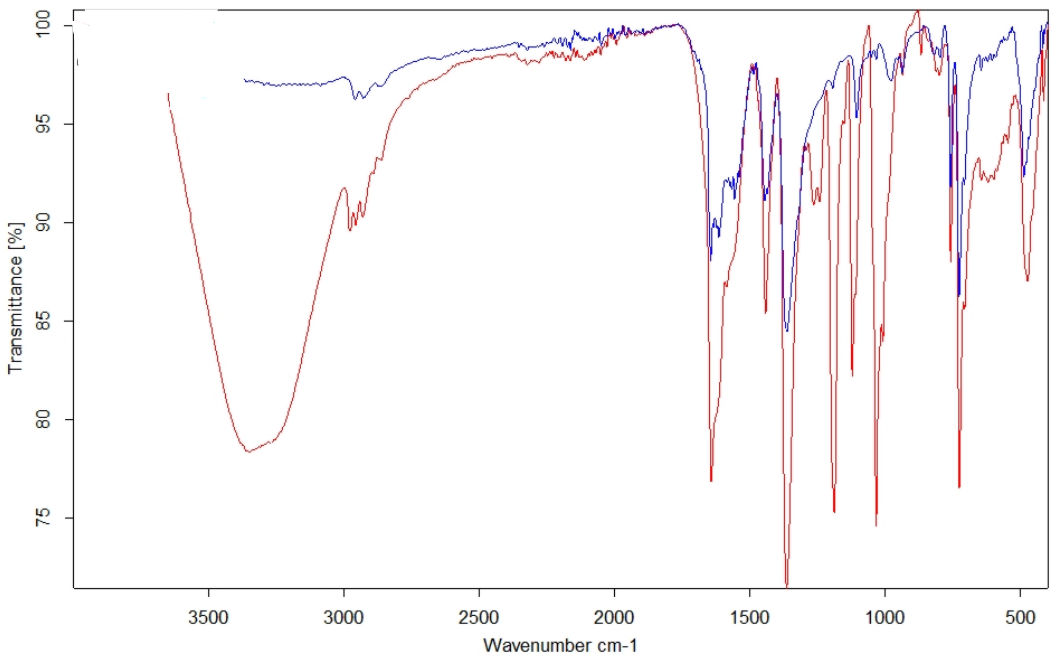

Figure S61. FTIR spectrum of activated HKUST-1 post-synthetically modified with 3-PA (blue) and same MOF as 1:1 with KEX (red).

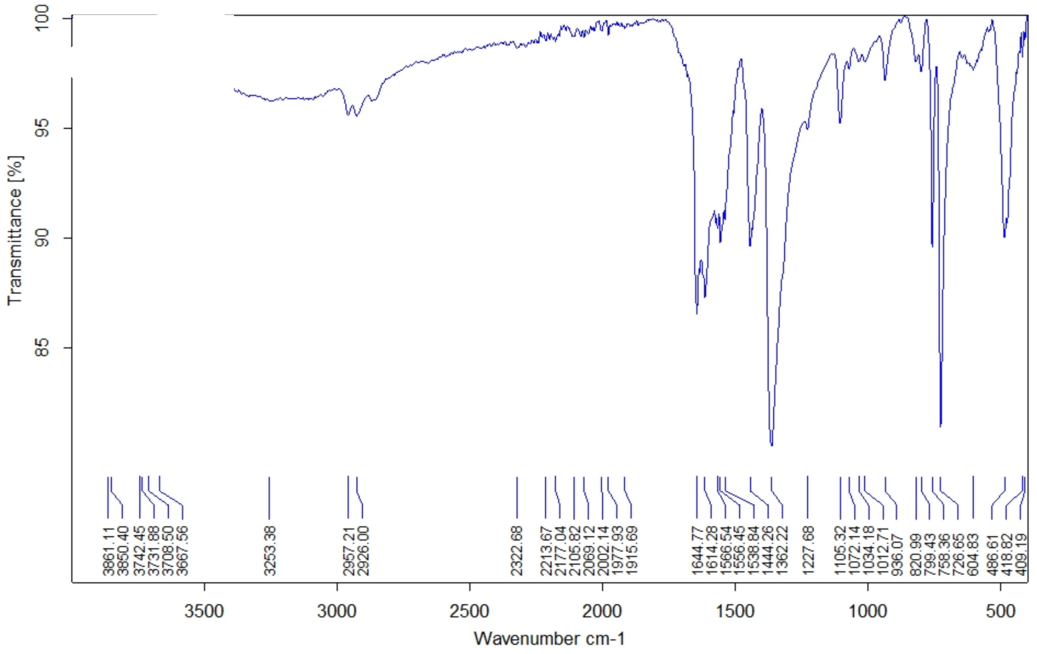

Figure S62. FTIR spectrum of activated HKUST-1 post-synthetically modified with 4-PA.

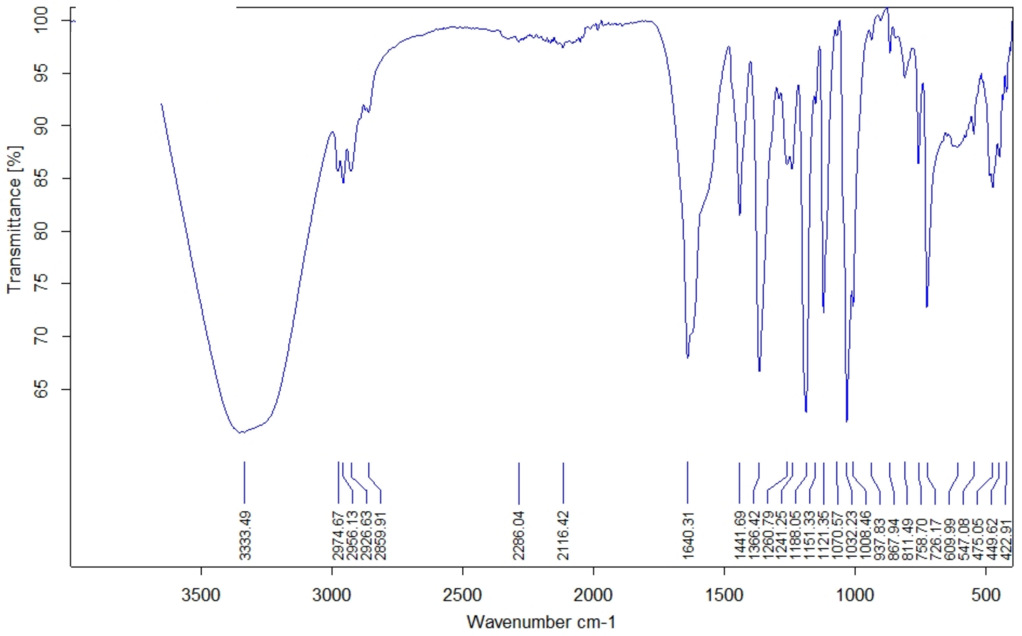

Figure S63. FTIR spectrum of activated HKUST-1 post-synthetically modified with 4-PA 1:1 with KEX.

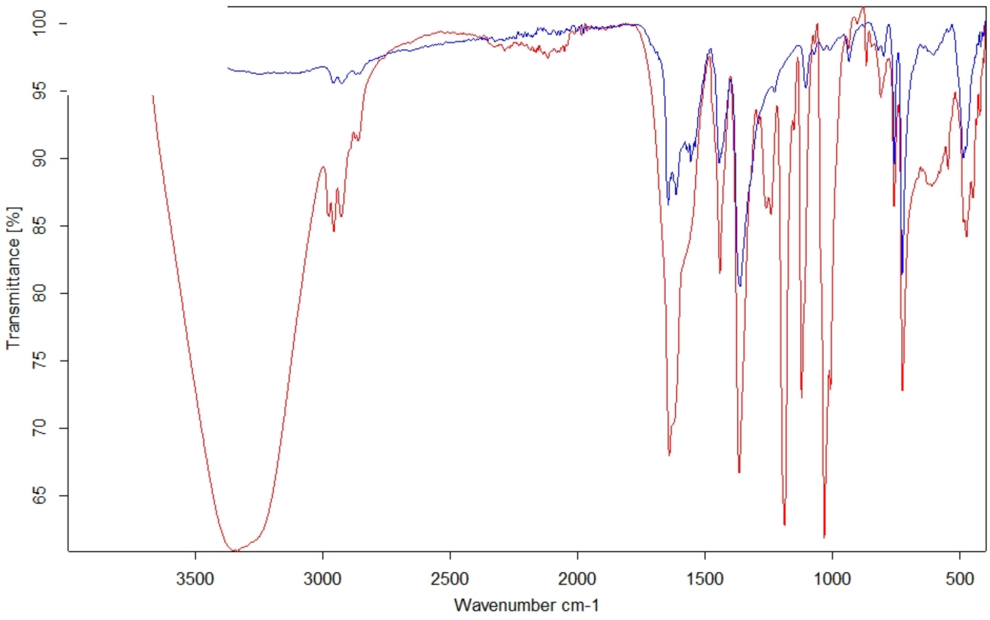

Figure S64. FTIR spectrum of activated HKUST-1 post-synthetically modified with 4-PA (blue) and same MOF with KEX (1:1) (red).

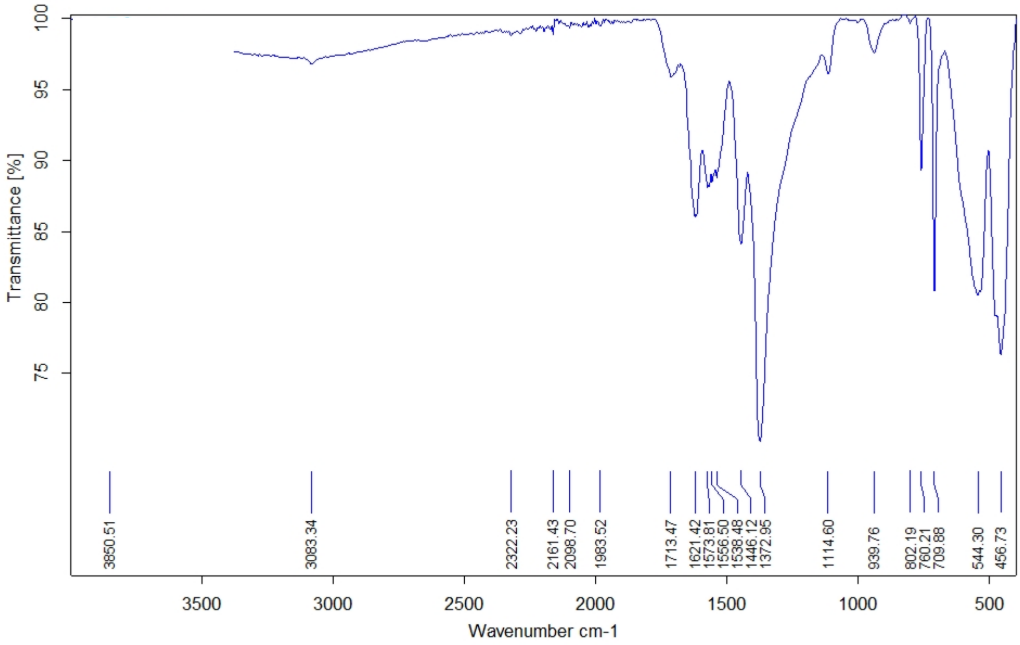

Figure S65. FTIR spectrum of activated MIL-100(Fe).

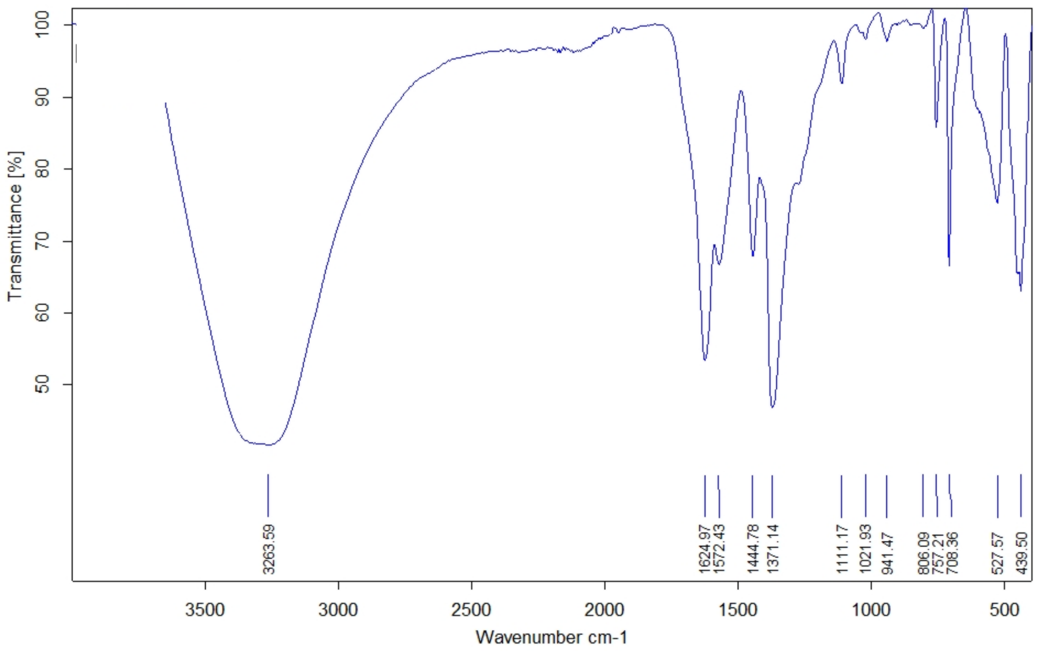

Figure S66. FTIR spectrum of activated MIL-100(Fe):KEX 1:1.

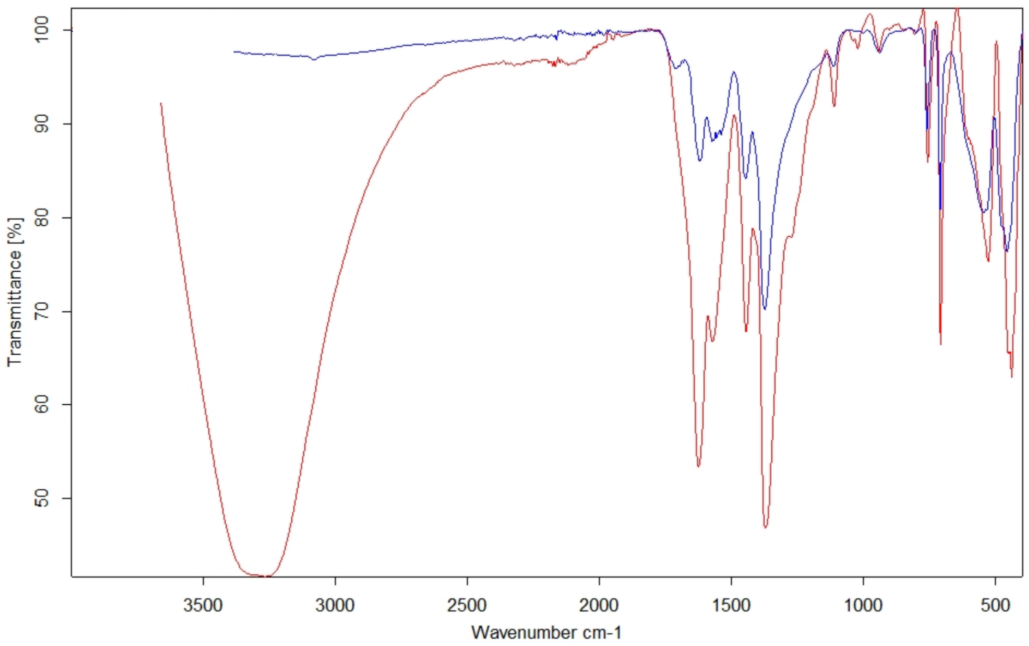

Figure S67. FTIR spectrum of activated MIL-100(Fe) (blue) and MIL-100(Fe):KEX 1:1 (red).

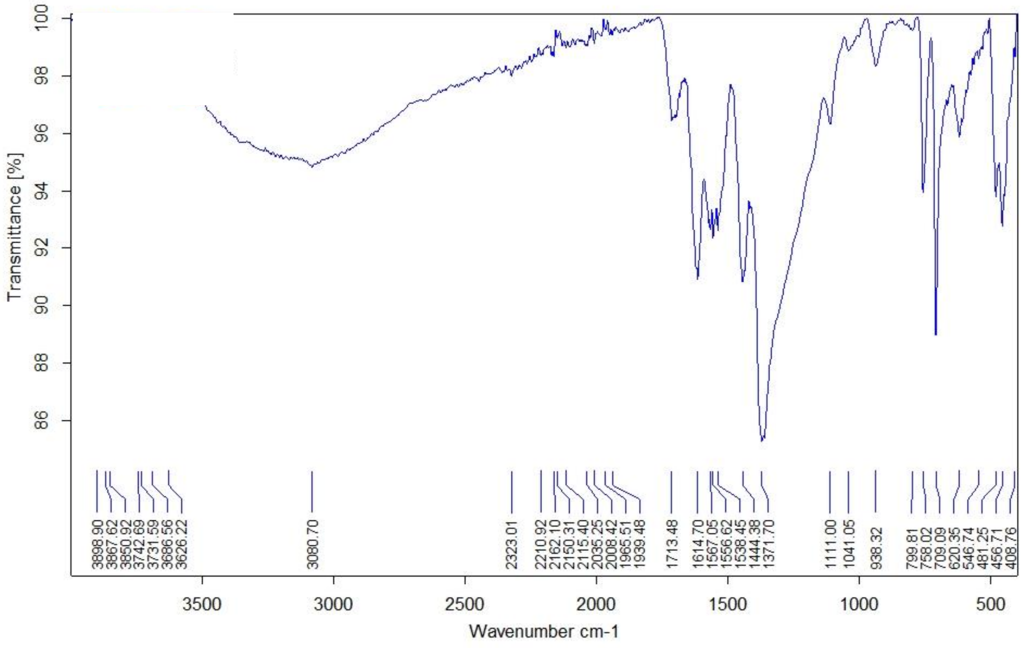

Figure S68. FTIR spectrum of activated mechanochemically synthetized MIL-100(Fe).

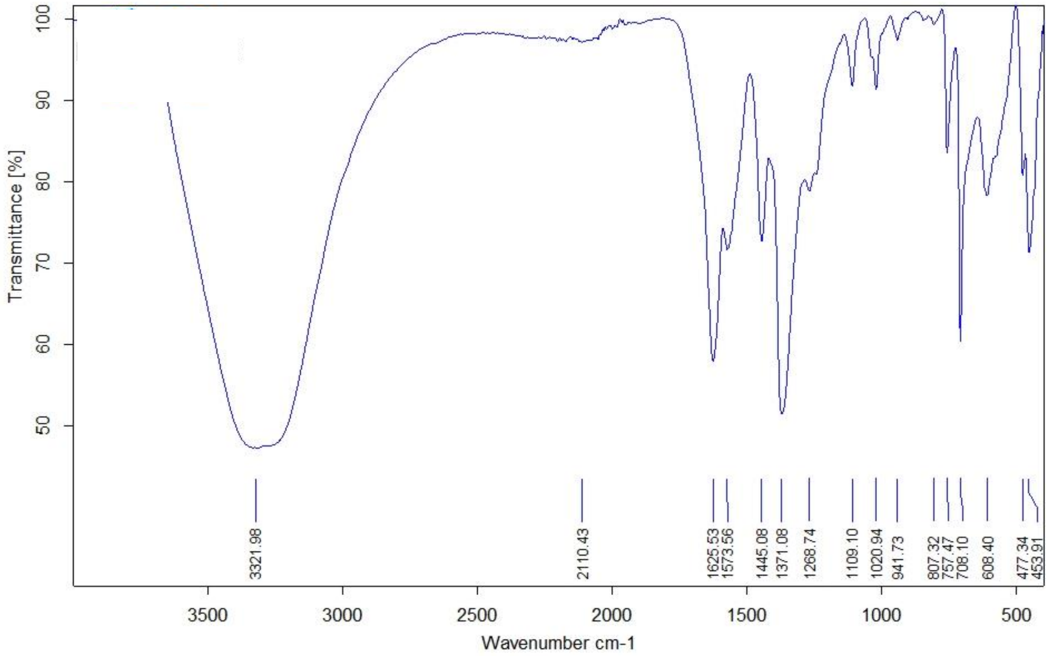

Figure S69. FTIR spectrum of activated mechanochemically synthetized MIL-100(Fe):KEX 1:1.

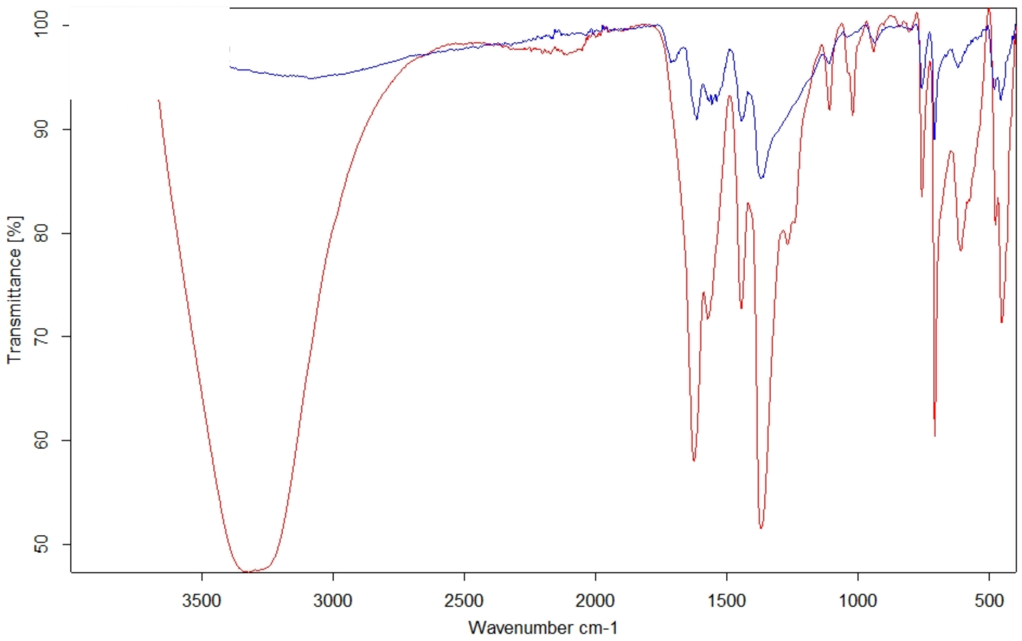

Figure S70. FTIR spectrum of activated mechanochemically synthetized MIL-100(Fe) (blue) and same MOF with KEX 1:1 (red).

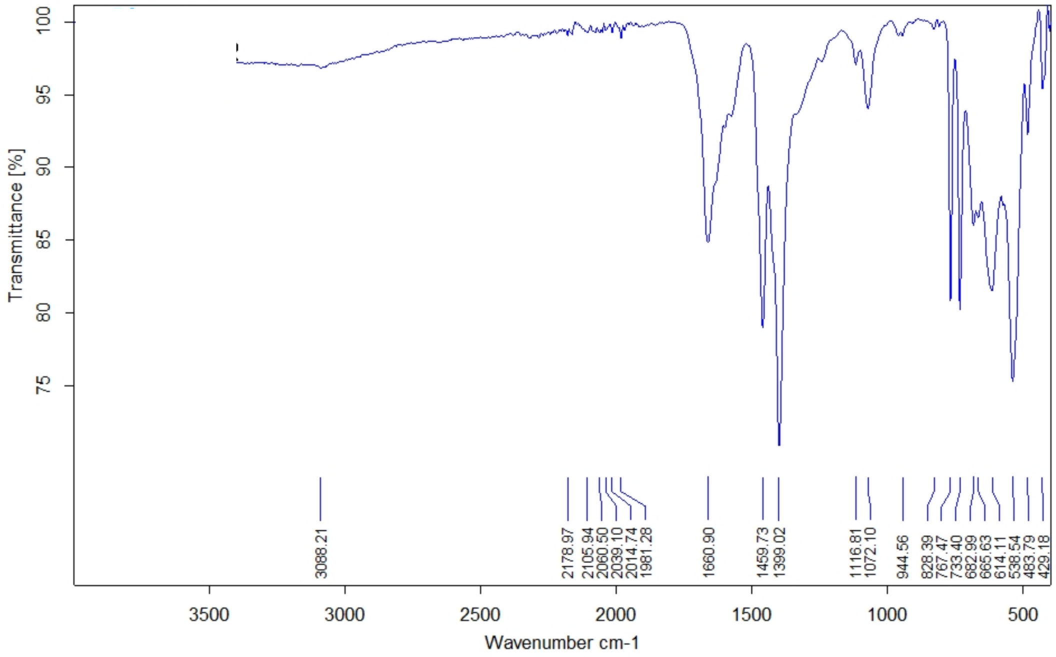

Figure S71. FTIR spectrum of activated 1:1 MIL-100(Al):MIL-96(Al).

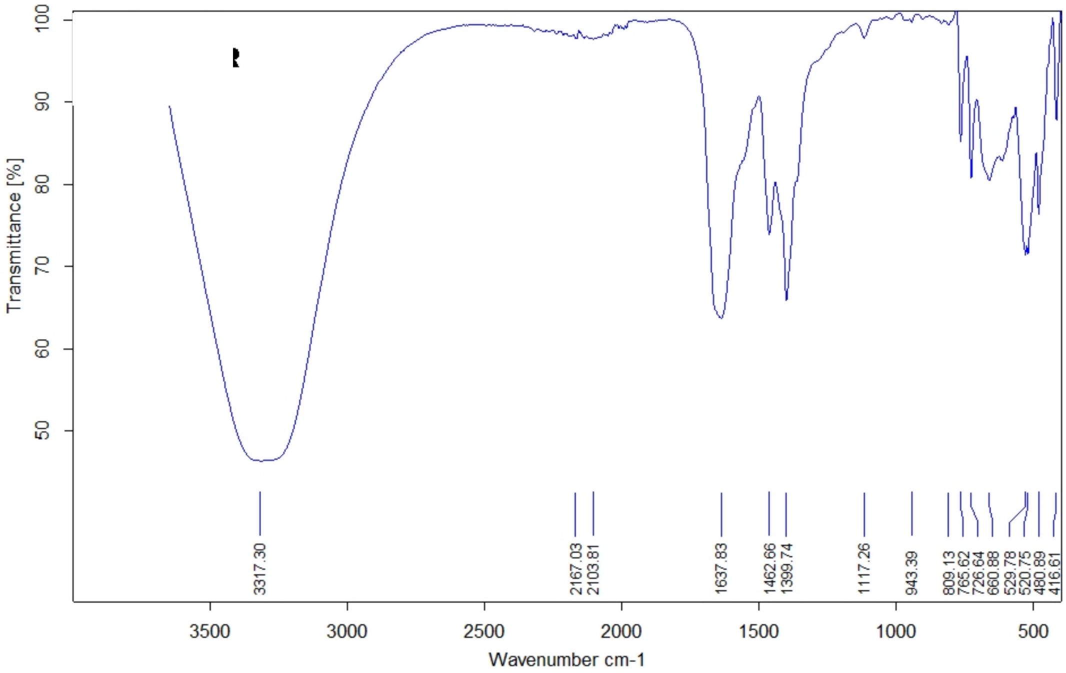

Figure S72. FTIR spectrum of activated 1:1 MIL-100(Al):MIL-96(Al) with KEX (1:1).

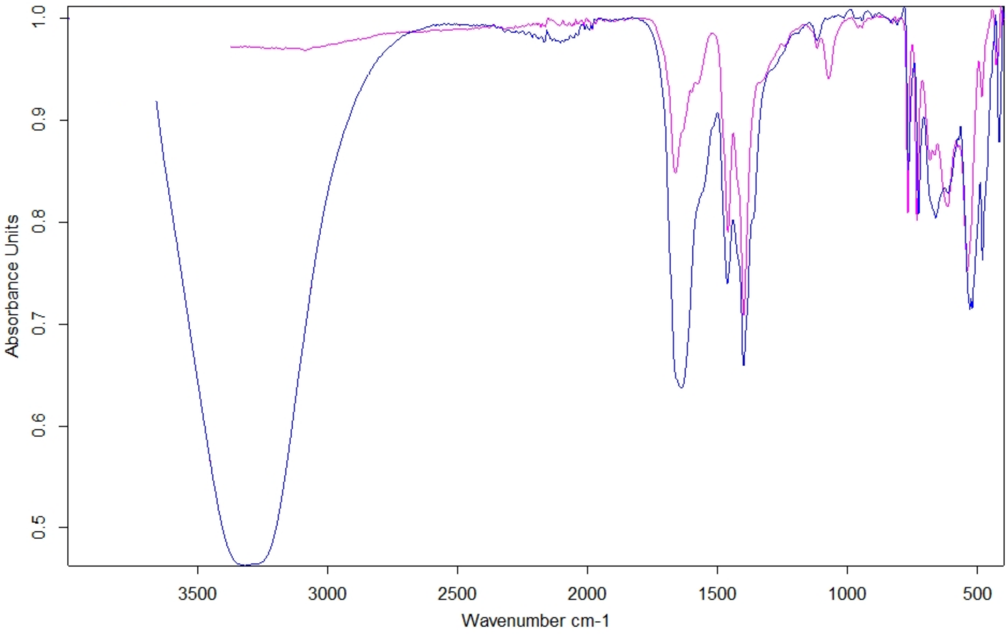

Figure S73. FTIR spectrum of activated 1:1 MIL-100(Al):MIL-96(Al) (violet) and 1:1 MIL-100(Al):MIL-96(Al) KEX 1:1 (blue).

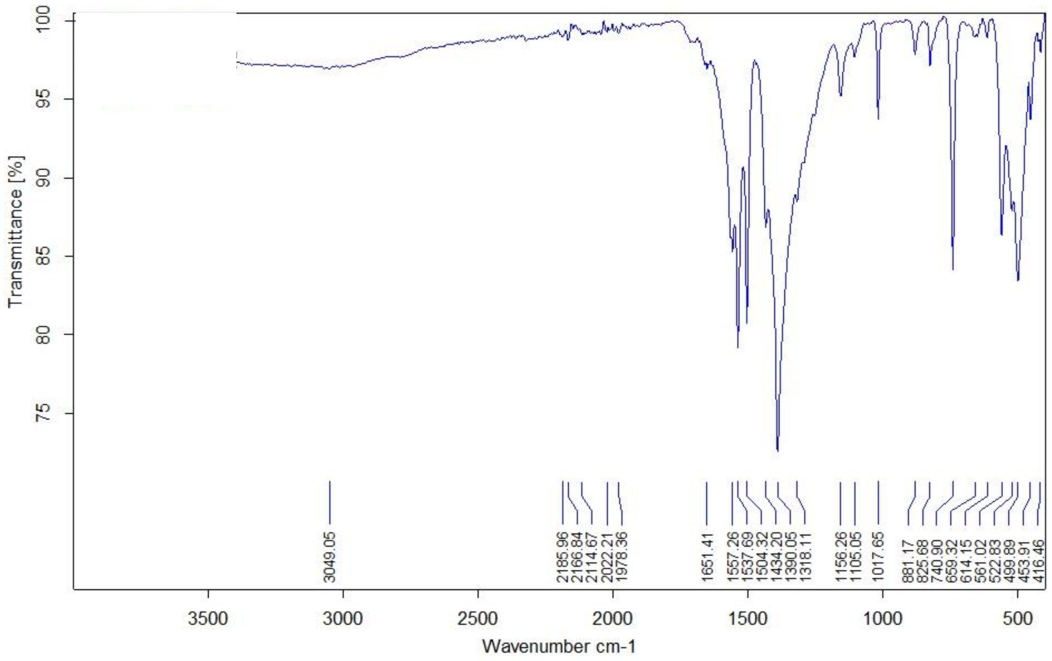

Figure S74. FTIR spectrum of activated UiO-66.

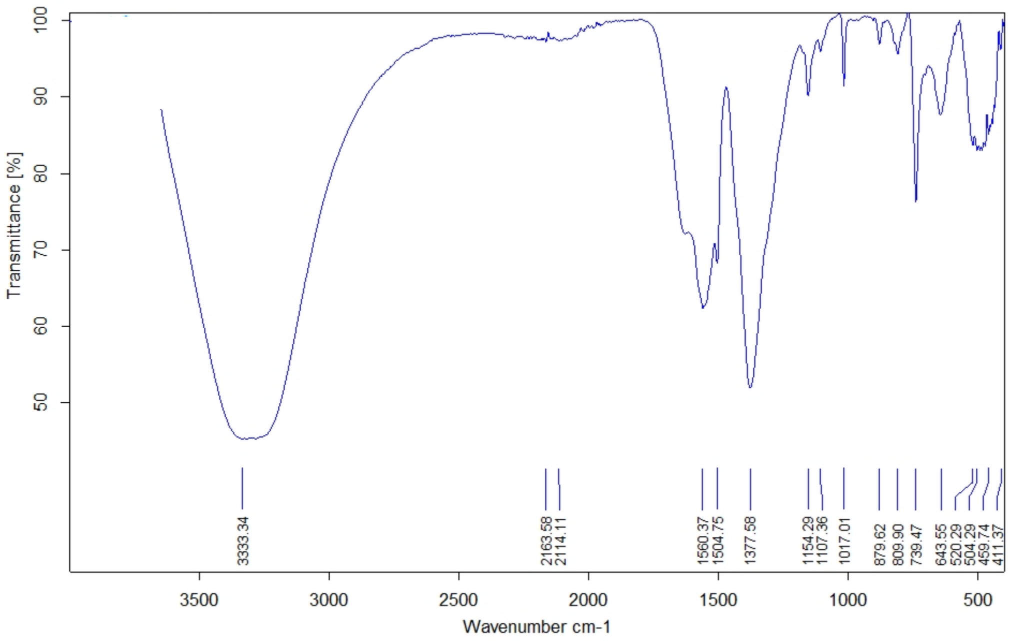

Figure S75. FTIR spectrum of activated UiO-66:KEX 1:1.

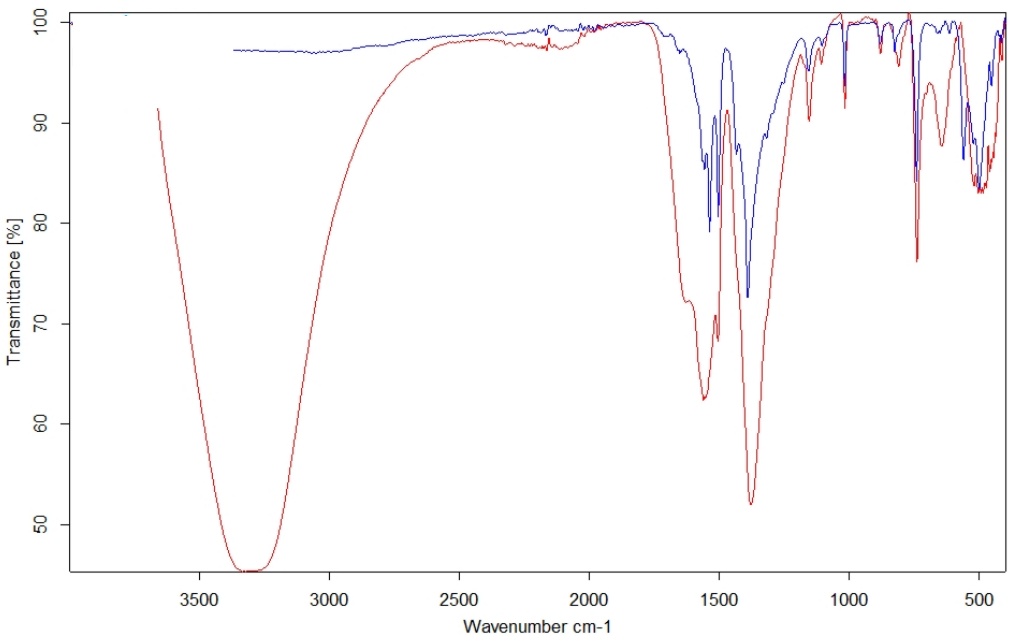

Figure S76. FTIR spectrum of activated UiO-66 (blue) and activated UiO-66:KEX 1:1 (red).

References

[1] Biemmi, E.; Christian, S.; Stock, N. & Bein, T., High-throughput screening of synthesis parameters in the formation of the metal-organic frameworks MOF-5 and HKUST-1, Micropor. Mesopor. Mat. **2009**, *117*, 111-117.

[2] Hwang, Y. K.; Hong, D. Y.; Chang, J. S.; Jhung, S. H.; Seo, Y. K.; Kim, J.; Vimont, A.; Daturi, M.; Serre, C. & Férey, G., Amine Grafting on Coordinatively Unsaturated Metal Centers of MOFs: Consequences for Catalysis and Metal Encapsulation, Angewandte Chemie International Edition, **2008**, *47*(22), 4144–4148.

[3] Montoro, C.; García, E.; Calero, S.; Pérez-Fernández, M. A.; López, A. L.; Barea, E. & Navarro, J. A. R., Functionalisation of MOF open metal sites with pendant amines for CO_2 capture, Journal of Materials Chemistry, **2012**, *22*(20), 10155–10158.

[4] Mahmoodi, N. M.; Abdi, J.; Oveisi M.; Asli M. A. & Vossoughi, M., Metal-organic framework (MIL-100 (Fe)): Synthesis, detailed photocatalytic dye degradation ability in colored textile wastewater and recycling, Materials Research Bulletin, **2018**, *100*, 357-366.

[5] Han, L; Qi, H.; Zhang, D.; Ye, G.; Zhou, W.; Hou, C.; Xu, W. & Sun, Y., A facile and green synthesis of MIL-100(Fe) with high-yield and its catalytic performance, New J. Chem. **2017**, *41*, 13504-13509.

[6] Splith, T.; Pantatosaki, E.; Kolokathis, P. D.; Fröhlich, D.; Zhang, K.; Füldner, G.; Chmelik, C.; Jiang, J.; Henninger, S. K.; Stallmach, F. & Papadopoulos, G. K., J. Phys. Chem. C. **2017**, *121*(33), 18065-18074.

[7] Qiu, J.; Feng, Y.; Zhang, X.; Jia, M. & Yao, J., Acid-promoted synthesis of UiO-66 for highly selective adsorption of anionic dyes: Adsorption performance and mechanisms, J. Coll. Interf. Sci. **2017**, *499*, 151-158.

[8] Degen, T.; Sadki, M.; Bron, E.; König, U.; Nénert, G., The HighScore suite, Powder Diffraction 29 S2**, 2014**, S13-S18.

[9] Groom, C. G.; Bruno, I. J.; Lightfoot, M. P., and Ward, S. C., The Cambridge Structural Database, Acta Cryst. **2016**, B72, 171–179.

[10] Chui, S. S.-Y.; Lo, S. M.-F.; Charmant, J. P. H.; Orpen, A. G. And Williams, I. D., A Chemically Functionalizable Nanoporous Material $[Cu_3(TMA)_2(H_2O)_3]_n$, Science, **1999**, 283(*5405*), 1148–1150.

[11] Ahmed, A.; Hodgson, N.; Barrow, M.; Clowes, R.; Robertson, C. M.; Steiner, A.; McKeown, P.; Bradshaw, D.; Myersa, P. and Zhang, H., Macroporous metal–organic framework microparticles with improved liquid phase separation, J. Mater. Chem. A, **2014**, 2, 9085.

[12] Horcajada, P.; Surblé, S.; Serre, C.; Hong, D.-Y.; Seo, Y.-K.; Chang, J.-S.; Grenéche, J.-M.; Margiolakid, I. and Férey, G., Synthesis and catalytic properties of MIL-100(Fe), an iron(III) carboxylate with large pores, Chem. Commun., **2007**, 2820–2822.

[13] Férey, G.; Serre, C.; Mellot-Draznieks, C.; Millange, F.; Surblé, S.; Dutour, J. and Margiolaki, I., A Hybrid Solid with Giant Pores Prepared by a Combination of Targeted Chemistry, Simulation, and Powder Diffraction, Angew. Chem. Int. Ed. **2004**, 43, 6296 –6301.

[14] Benzaqui, M.; Pillai, R. S.; Sabetghadam, A.; Benoit, V.; Normand, P.; Marrot, J.; Menguy, N.; Montero, D.; Shepard, W.; Tissot, A.; Martineau-Corcos, C.; Sicard,C.; Mihaylov, M.; Carn, F.; Beurroies, I.; Llewellyn, P.L.; De Weireld, G.; Hadjiivanov, M.; Gascon, J.; Kapteijn, F.; Maurin, G.; Steunou, N. and Serre, C. Revisiting the Aluminum Trimesate-Based MOF (MIL-96): From Structure Determination to the Processing of Mixed Matrix Membranes for CO_2 Capture, Chem. Mater. **2017**, 29, 10326–10338.

[15] Perfecto-Irigaray, M.; Beobide, G.; Castillo, O.; da Silva, I.; García-Lojo, D.; Luque, A.; Mendia, A. and Pérez-Yáñez, S., $[Zr_6O_4(OH)_4(\text{benzene-1,4-dicarboxylato})_6]$n: a hexagonal polymorph of UiO-66, Chem. Commun. **2019**, *55*, 5954–5957.
